# Synthesis of Cyclopropene‐Modified Fatty Acids Allows Single‐Cell Quantification of Uptake by Immune Cells

**DOI:** 10.1002/anie.202525040

**Published:** 2026-05-28

**Authors:** Luuk Reinalda, Graham A. Heieis, Laura Bogue Edgerton, Kristine Bertheussen, Diana Torres‐García, Marouane el Boujadayni, Kas Steuten, Jeroen M. Punt, Wouter P. F. Driever, Connor Corrigan, Xinyuan Wang, Bart Everts, David Finlay, Mario van der Stelt, Sander I. van Kasteren

**Affiliations:** ^1^ Division of Chemical Biology and Immunology Leiden Institute of Chemistry and the Institute for Chemical Immunology Leiden University Leiden the Netherlands; ^2^ Department of Parasitology Leiden University Medical Center Leiden the Netherlands; ^3^ Department of Molecular Physiology Leiden University Leiden the Netherlands; ^4^ School of Biochemistry and Immunology Trinity Biomedical Sciences Institute Trinity College Dublin Dublin Ireland

**Keywords:** bioorthogonal chemistry, immunometabolism, inverse electron‐demand Diels–Alder reaction, T cell activation

## Abstract

Immune cell activity is strongly influenced by the nutrients present during activation. The effects of specific fatty acids (FAs) can be particularly complex, with them exerting diverse and sometimes opposing effects on immune cells. These functional differences are thought to stem from structural differences between the FAs, leading to altered cellular handling. However, chemical tools to directly probe these aspects remain limited. Here, we report the design and synthesis of saturated, unsaturated, and polyunsaturated cyclopropenyl fatty acids, each incorporating a minimal one‐carbon cyclopropene moiety as a bioorthogonal click handle. This motif enables rapid and live‐cell‐compatible labeling via the inverse electron‐demand Diels–Alder reaction, providing a versatile platform to trace FA behavior in biological systems. Application of these synthetic cyclopropenyl FAs in primary immune cell mixtures reveals distinct uptake patterns between different cell types, with polyunsaturated analogues showing strong uptake across all immune cell types. Complementary metabolic and proteomic analyses suggest that this uptake results in biological differences between high/low uptake immune populations, highlighting the utility of cyclopropenyl FA probes for dissecting lipid uptake in the context of immune cell biology.

## Graphical abstract

Cyclopropene fatty acids have been made that allow the study of fatty acid uptake in complex mixtures of immune cells with single cell resolution and combined with metabolic and proteomic analysis so that for the first time nutrient uptake can be correlated to the phenotype of single immune cell.

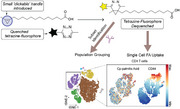

## 1 Introduction

Fatty acids (FAs) play essential roles in every mammalian cell type [1, 2]. They serve as structural building blocks of membranes [3], as fuel to highly energy demanding cells, such as activated immune cells and cancer cells [4], and as signaling mediators, particularly in immunological processes [5, 6, 7, 8]. The identification of the (patho)physiological roles of FAs in immune activation and the shaping and resolution of the immune response is leading to the discovery of new therapeutic targets [9, 10, 11].

The FAs used by immune cells can be synthesized de novo by the enzymes amino acetyl carboxylase and fatty acid synthase [12]. However, the uptake of exogenous FAs is also a key factor in fueling their metabolic and signaling demands. Specific exogenous lipids can therefore shape the immune response [13, 14]. For example, exogenous oleic acid is an essential FA for T‐cell activation [15]. Without uptake of this FA, T‐cell activation is severely hampered. It can also have more specific effects, with high uptake skewing the developing T‐cells toward specific subtypes [16, 17]. The saturated fatty acids palmitic and stearic acid, despite minor structural differences, can in turn be toxic to activating T‐cells [18]. This can—in part at least—be rescued by other FAs [19]. Linoleic, palmitoleic, and arachidonic acid show more complex behaviors. Their exogenous uptake has been shown to potentiate T‐cell activation [20], but in other studies to suppress it. These effects could be concentration‐dependent [6, 7, 8, 9, 10, 11, 12, 13, 14, 15, 16, 20, 21, 22].

These contradicting biological findings highlight the need to study the uptake of individual FAs in immune cells at the single cell level. The effects of each FA on the target cell is dependent on both the nature and amount of the specific FA that specific cell has taken up. Previous studies have made use of fluorophore labelled fatty acid analogues (e.g., BODIPY‐C12/16, TopFluor oleic acid) [23, 24]. They mimic long chain fatty acids by including an apolar fluorophore in the hydrophobic tail of the fatty acid [25]. The approach is live cell compatible, but the large pendant aromatic fluorophore, which is itself targeted to lipid droplets, disrupts the native structure and biology of the fatty acid. The fluorophore likely having much more of an impact on FA structure than the chain length and/or the presence/absence of double bonds, limiting their suitability for studying uptake of lipids with subtle structural differences [26].

**FIGURE 1:**
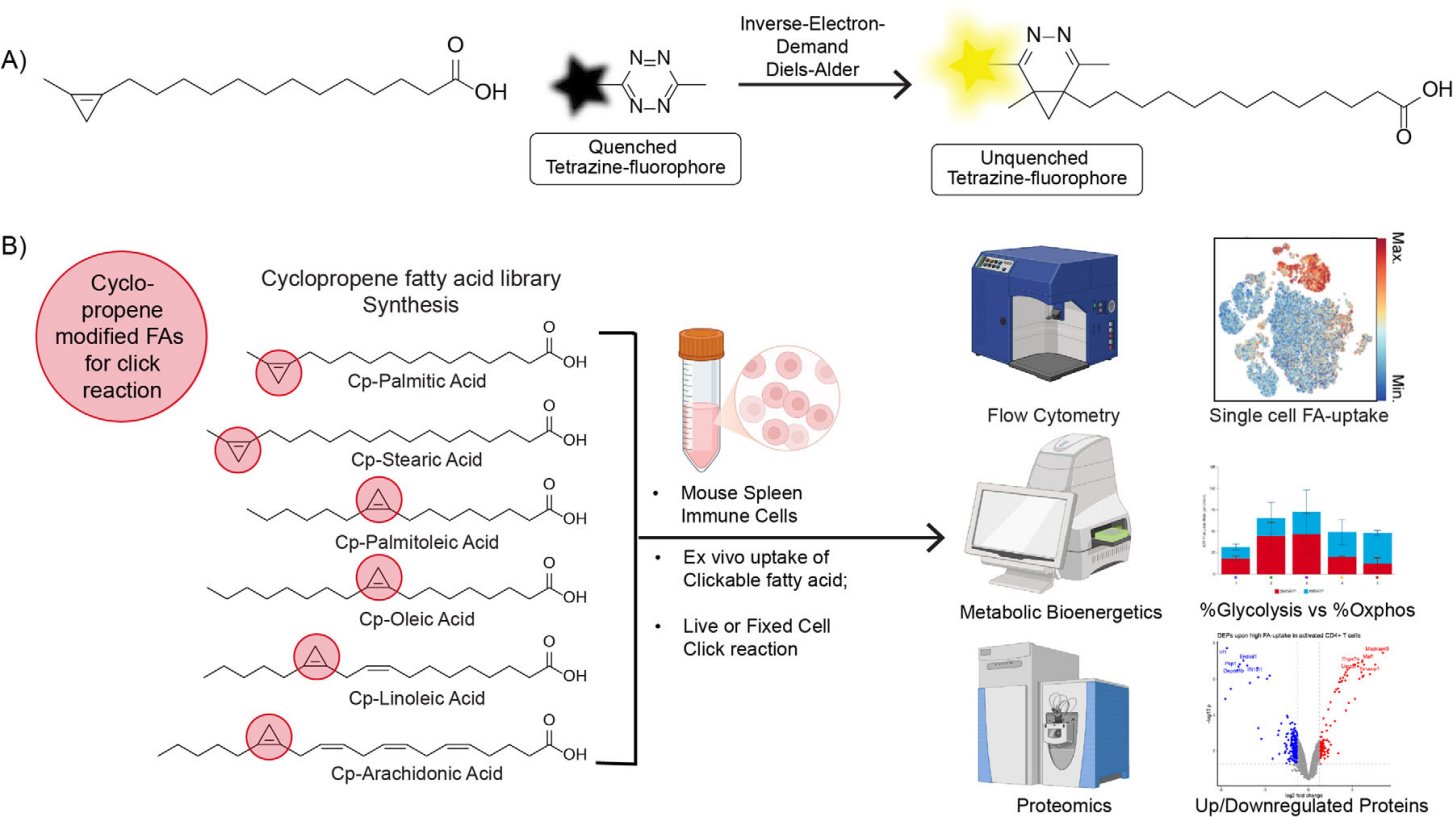
Overview of the approach and synthetic targets. (A) The inverse‐electron‐demand Diels–Alder reaction is a mild live‐cell compatible click reaction between strained alkenes, such as cyclopropenes and electron‐poor tetrazines. (B) The library of cyclopropene‐modified fatty acid synthesized and evaluated in this paper. The fatty acids were incubated with mixtures of primary immune cells and analyzed for single cell fatty acid uptake by flow cytometry, subjected to sorting and metabolic analysis and proteomics.

Bioorthogonal click chemistry is one strategy to overcome these limitations [27, 28]. Instead of introducing the large pendant fluorophore before the biological experiment, fatty acid analogues modified with small click‐reactive groups, such as alkynes and azides, have been used to study uptake. The fluorophore is then introduced after the biological reactions using a bioorthogonal ligation reaction, such as the strain‐promoted/copper‐catalyzed azide‐alkyne cycloadditions (SPAAC/CuAAC) [29, 30]. These small chemical functionalities allow for fluorophore labeling after completion of the biological time courses, while keeping structural fidelity to natural FA [31, 32, 33]. Alkyne analogues of saturated fatty acid (SFA) palmitic acid, and monounsaturated fatty acid (MUFA) oleic acid have, for example, been used to mark differences in the uptake of exogenous FAs in differently polarized bone marrow derived macrophages [34]. They were also employed to study the CD36‐mediated trans‐endothelial cells transport [35], and more recently to quantify the subcellular localization of different lipids after uptake, when combining alkynes with photo‐crosslinking [36]. An alkyne analogue of arachidonic acid was also reported and used to reveal the critical role of this polyunsaturated fatty acid (PUFA) in megakaryocyte differentiation [37]. An alkyne‐containing analogue of the ω‐3 PUFA α‐linolenic acid was used to unravel *Staphylococcus aureus* capacity to escape antimicrobial FAs [38]. The CuAAC is, therefore, a very powerful approach for FA and lipid labeling in fixed cells. Live‐cell compatibility is limited due to the cytotoxicity of the required copper catalyst toward immune cells. Azide‐modified lipids can be visualized using live‐cell compatible strain promoted azide‐alkyne cycloadditions [30, 39]. A recent example is the use of azido‐fatty acids with organelle‐targeted cyclooctynes to study subcellular localization of fatty acids in cell lines [40]. The cyclooctyne fluorophores used to detect azides do have some limitations. They react with competitive rates with free intracellular thiols such as glutathione [41]. This is a particular problem, because free‐thiol levels in immune cells are high and can change upon activation [42]. This would render the background variable. We and others have; therefore, focused on another live cell‐compatible reaction: the inverse‐electron‐demand Diels–Alder click reaction (IEDDA, Figure 1A).

The IEDDA is a live‐cell compatible bioorthogonal click reaction between strained alkenes and tetrazines [43]. The first applications of this reaction showed its favorable properties for live cell studies [44, 45]. The reaction is fast and does not require a catalyst. In addition, the tetrazines used in this reaction quench pendant fluorophores. This property is lost upon reaction with the alkene, leading to fluorescence turn‐on upon ligation [44, 45, 46]. The reaction rate is similar to that of the CuAAC [38], without the need for a toxic catalyst. This has led to a flurry of activity for using cyclopropenes (Cps) and other IEDDA‐reactive alkenes in chemical biology. Alkene‐modified amino acid analogues have been applied to protein labeling [47, 48, 49, 50], nucleotide analogues to label RNA/DNA [51, 52], and carbohydrate analogues for glycan imaging [53, 54, 55, 56]. One example of note is from the Schepartz group, who modified a ceramide headgroup with a *trans‐*cyclooctene and used it to image the Golgi apparatus [57].

One downside is that many of the strained alkenes used in the reaction are very large, which would alter the FA structure significantly [46]. One group stands out in this context though: 1,2‐ and 1,3, and 3,3,‐cyclopropenes. These are the smallest strained alkenes that can react in the IEDDA reaction (Figure 1A) [54, 58, 59, 60]. A few examples of cyclopropenes for studying lipid uptake have been reported [61, 62]. For example, Devaraj and coworkers attached a 1,3‐cyclopropene tag to the polar head of phospholipids and incorporated those in membranes of a human breast cancer cell line, which was visualized by confocal microscopy [59]. However, most methods rely on the incorporation of the Cp (or other IEDDA reactive group) into the headgroup of a lipid. This approach is not compatible with the study of FAs as the carboxylic acid is a key recognition motif in biology. Our group has recently reported the use of the naturally occurring cyclopropene containing FA sterculic acid, an analogue of oleic acid (OA) bearing an extra methylene bridging the double bond, as a tool for imaging lipid uptake [63, 64]. This lipid was shown to be taken up in cell lines and was incorporated in membranes of these immortalized cells. It could be used for fixed and live‐cell imaging, making it a powerful reagent that can be used as analogue for OA [63]. However, no other naturally occurring cyclopropene FAs exist that can be used analogously to this oleic acid analogue for other FAs.

**FIGURE 2:**
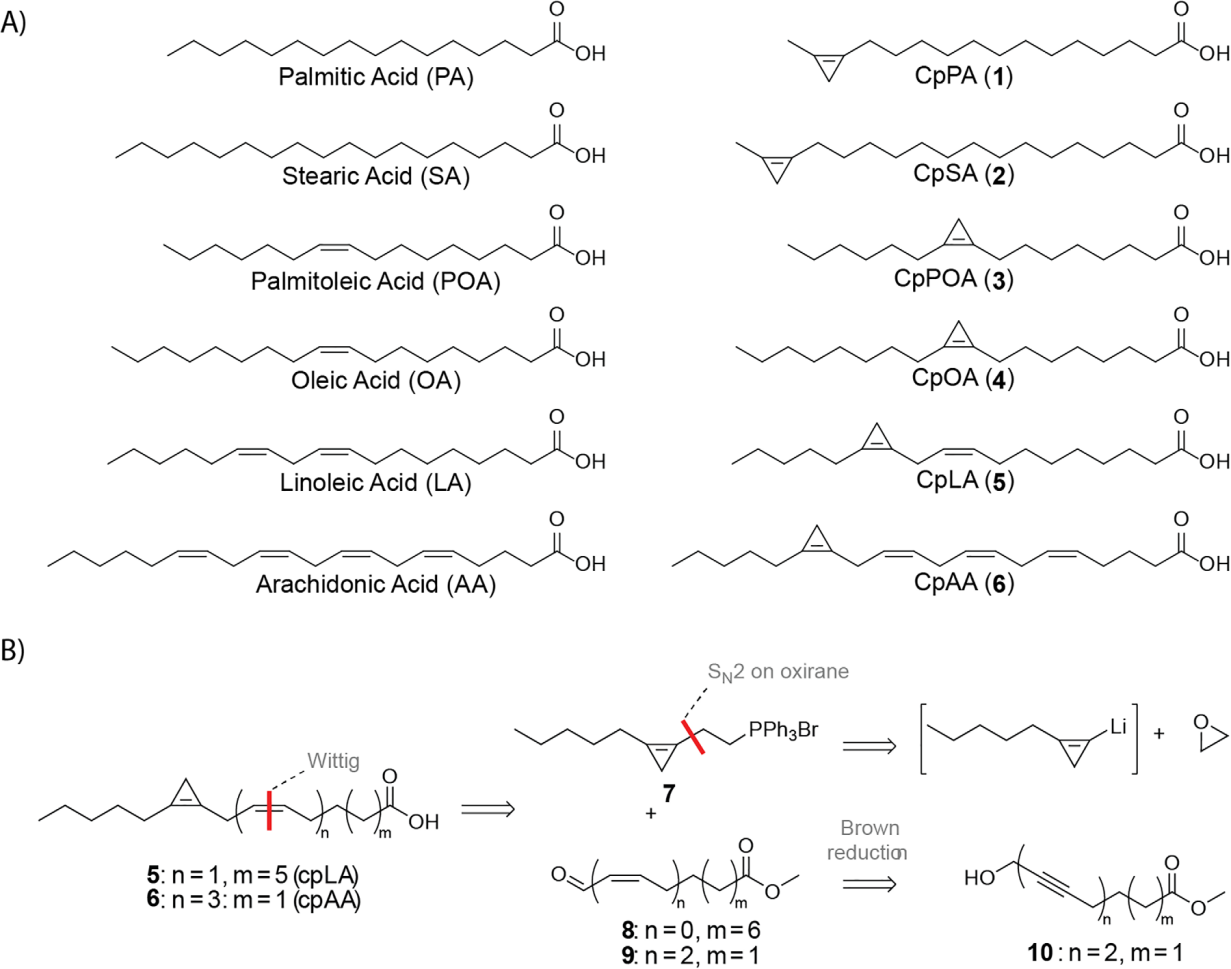
(A) (Left side) Key fatty acids in T‐cell metabolism and their cyclopropene containing counterparts (**1–6**) made in this manuscript. (B) Retrosynthetic analysis for PUFAs linoleic acid **5** and arachidonic acid **6**.

To address this lack of IEDDA‐reactive Cp‐containing lipids, we here describe the synthesis of sterculic acid and five new synthetic cyclopropene fatty acid analogues (CpFAs) and use these to study FA‐uptake by primary immune cells in mixed immune populations; with a focus on T‐cells. The aim was to quantify the relative uptake of different FAs for specific immune cell populations, but to also quantify the relative uptake between different cell types (Figures 1B and 2A). All six CpFAs showed reacted with tetrazine fluorophores in fixed and live cells and could thus be used for this purpose. Making use of the live‐cell compatibility of the reaction in combination with live cell sorting and metabolic analysis, we also showed that high/low OA‐uptake unstimulated T‐cells rely on oxidative phosphorylation and glycolysis differentially, whereas activated cells do not. Combination of the uptake assay with cell sorting and proteomics showed high uptake naïve T‐cells have higher mTOR activity, whereas the difference in uptake in activated T‐cells correlates with changes in levels of downstream FA processing enzymes, supporting the hypothesis that uptake is driven by the rate of intracellular FA removal.

## 2 Results and Discussions

### 2.1 Design and Synthesis of Cyclopropene Fatty Acids

The main aim of this manuscript was to design and synthesize analogues of six key fatty acids important in immunology with minimal structural modification, so that the differences in their uptake could be compared (Figure 1B). For the saturated fatty acids, we envisaged that fatty acids modified with an ω‐terminal Cp‐group would best mimic the saturated FAs palmitic and stearic acid, leading to the chosen design of **1** and **2**. For any lipid bearing a single double bound, we envisaged a formal replacement of the double bond with the 1,2‐cyclopropene group. Cp‐palmitoleic acid **3** was thus designed following nature's design of sterculic acid **4**. For the polyunsaturated FAs, we chose to substitute the ω‐6 double bonds of linoleic acid and arachidonic acid to yield designs **5** and **6**, to not disrupt the central unsaturation pattern of the parent fatty acid. We also chose to keep the linear chain length identical to that of the parent lipid, leading to a nominal size increase of one carbon in the Cp‐analogues.

The polyunsaturated CpFAs were the most challenging synthetically. Retrosynthetic analysis (Figure 2) had us disconnect across the double bond adjacent to the Cp‐group, which can be formed using a Wittig reaction. This approach identified triphenylphosphonium bromide building block **7** as a key intermediate to be accessed by an S_N_2‐reaction between a cyclopropyl‐lithium species and oxirane [65]. For the synthesis of the aldehyde building block **9**, required for the synthesis of CpAA **6**, a Brown reduction of the bis‐alkyne building block **10** to bis‐*cis*‐alkene **21** was proposed.

**SCHEME 1:**
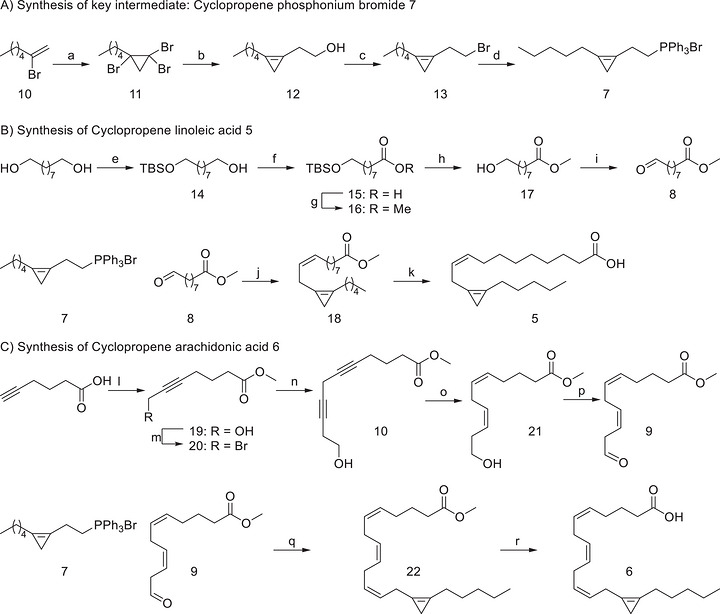
Synthesis of PUFAs linoleic acid (5) and arachidonic acid (6). (A) Synthesis of key intermediate cyclopropene phosphonium bromide **7**. Reagents and conditions: (a) BBr_3_, DCM, −78°C to rt, 3 h, 52% yield. (b) *n*‐Buli, BF_3_·OEt_2_, oxirane, THF, −78°C to 0°C, 2 h, 49% yield. (c) NBS, PPh_3_, DCM, 0°C to rt, 2 h, 59% yield. (d) PPh_3_, MeCN, 82°C, 18 h, 82% yield. (B) Synthesis of cyclopropene linoleic acid **5**. Reagents and conditions: (e) TBSCl, imidazole, DCM, rt, 3 h, 64% yield. (f) TEMPO/BAIB, 2:1 MeCN/water, rt, 3 h, 92% yield. (g) MeI, K_2_CO_3_, DMF, rt, 18 h, 86% yield. (h) TBAF, DCM, 0°C to rt, 5 h, 93% yield. (i) DMP, DCM, 0°C to rt, 1 h, 78% yield. (j) NaHMDS, THF, −78°C to 0°C, 2.5 h, 44% yield. (k) NaOH (aq.), 1:1 THF/EtOH, 70°C, 2 h, 73% yield. (C) Synthesis of cyclopropene arachidonic acid **6**. Reagents and conditions: (l) EtMgBr, PFA, THF, 0°C–75°C, 18 h, then: SOCl_2_, MeOH, 0°C to rt, 1 h, 51% yield. (m) NBS, PPh_3_, DCM, 0°C to rt, 1 h, 82% yield. (n) CuI, NaI, K_2_CO_3_, but‐3‐yn‐1‐ol, DMF, rt, 18 h, 72% yield. (o) NaBH_4_, Ni(OAc)_2_·4 H_2_O, ethylenediamine, H_2_ (g), MeOH, rt, 2 h, 27% yield. (p) DMP, DCM, 0°C to rt, 1 h, 37% yield. (q) NaHMDS, THF, −78°C to 0°C, 2.5 h, 54% yield. (r) NaOH (aq.), 1:1 THF/EtOH, 70°C, 2 h, quant. yield.

The synthesis of polyunsaturated CpFAs **5** and **6** (Scheme 1) was achieved via a phosphonium bromide was positioned on the same fragment as the cyclopropene [66]. Incidentally, the inverse approach, where the aldehyde was introduced on the Cp‐containing fragment, did not work due to instability of the aldehyde (Scheme S2). Alcohol **12** was synthesized by ring‐opening of ethylene oxide, catalyzed by BF_3_·OEt in presence of the lithiated cyclopropene nucleophile from **11**. Appel‐like conditions using *N*‐bromosuccinimide on our 1,2‐substituted substrate afforded stable cyclopropene bromide **13** in 59% yield. Direct alkylation with dihaloalkanes did not work (Scheme S1). An overnight substitution reaction with triphenylphosphine under reflux in acetonitrile yielded the target cyclopropene phosphonium bromide **7** in 23% yield over three steps. Aldehyde **8** was synthesized in five steps from a diol by protecting the alcohol on one end with a silyl to yield compound **14**, which was oxidized and protected to yield methyl ester **15**. The silyl ether protecting group was removed and the alcohol **16** oxidized affording aldehyde **8**. The phosphorus ylide of **7** was formed in situ using NaHMDS and reacted with aldehyde **8** to yield the cyclopropene linoleic methyl ester **18**. Subsequent saponification yielded **5**, to the best of our knowledge the first example of the synthesis of a cyclopropene‐containing PUFA analogue. The synthesis of the polyunsaturated arachidonic acid‐analogue **6** was again achieved using a late stage Wittig reaction to form the C─C bond between aldehyde **9** and phosphonium bromide **7**, but instead uses the bis‐alkyne **10** as key intermediate (Scheme 1C) that could later be reduced stereoselectively to the bis‐*cis*‐alkene [67, 68, 69], as efforts to perform the Wittig using the bis‐alkene had failed (Scheme S3).

Alkynylation of paraformaldehyde with 5‐hexynoic acid using an excess of EtMgBr followed by esterification yielded methyl ester **19**, which was converted into the bromide **20** with an Appel‐like reaction. Then, a copper(I) mediated cross‐coupling with but‐3‐yn‐1‐ol afforded skipped diyne **10**. A selective reduction of the alkynes to alkenes was attempted using poisoned P‐2 nickel, as Lindlar's catalyst yielded inseparable *E*/*Z* mixtures [70, 71]. HPLC‐purification was required to separate diene **21** from its by‐products. A Wittig reaction between aldehyde **9** and phosphonium bromide **7** yielded cyclopropene arachidoyl methyl ester **22** that could be saponified to yield **6** (Scheme 1C).

**SCHEME 2:**
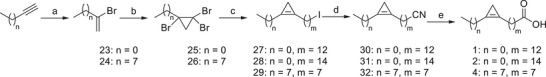
Synthesis of SFAs and MUFA 1, 2, and 4. Reagents and conditions: (a) BBr_3_, DCM, −78°C to rt, 3 h, 78% yield. (b) NaOH, CTAB, CHBr_3_, DCM, 0°C to rt, 18 h, 47%–57% yield. (c) *n*‐BuLi, diiodoalkane, THF, −78°C to 0°C, 4 h, 27%–56% yield. (d) NaCN, DMSO, 90°C, 1 h, 68%–89% yield. (e) NaOH, EtOH/water, reflux, 18 h, 37%–63% yield.

Various routes toward the synthesis of sterculic acid have been reported [72, 73, 74, 75]. Due to the structural similarity of Cp‐palmitoleic acid **3** to sterculic acid, we first opted for the most‐used synthetic route which involves a rhodium catalyzed [2+1] cycloaddition to form the cyclopropene moiety (Scheme S4). Nevertheless, this route was deemed unpractical due to experimental complexity and poor reproducibility [76]. Saturated CpFAs **1** and **2** and MUFA **4** were therefore synthesized using a tribromocyclopropane **23** as a cyclopropene precursor (Scheme 2) [77]. Treatment of **26** and **27** with two equivalents of *n*‐butyllithium (*n*‐BuLi) yielded a lithiated cyclopropene nucleophile via a double lithium‐halogen exchange and an elimination that could be reacted with a diiodoalkane to form the iodocyclopropenes [78, 79]. In the first step, bromination of the terminal alkyne with BBr_3_ yielded bromoalkene **24**. A dibromocarbene‐species was formed from bromoform by NaOH in a phase transfer catalytic system, which was reacted with the readily available alkene **23** and compound **24** to yield tribromocyclopropanes **25** and **26**. The reaction of the lithiated cyclopropene nucleophile with an excess of diiodoalkane afforded iodocyclopropenes **27–29**. These were converted to nitriles **30–32** by reacting them with sodium cyanide followed by basic hydrolysis to give the cyclopropene fatty acids **1**, **2**, and **4** in 2.5%–11.5% yield over five steps. Although the yield remained low, the experimental simplicity of the individual steps and higher reproducibility made this the route of choice for the synthesis of compounds **1** and **2**.

### 2.1 Optimizing CpFA Uptake in Mouse Immune Cells

With these six cyclopropene fatty acid analogues of immunologically relevant FAs [80, 81], we next assessed the suitability of these analogues to study fatty acid uptake in immune cells. We opted to optimize the reaction in primary immune cells, rather than cell lines, as the cancerous nature of cell lines heavily impacts their nutrient uptake behavior compared to non‐cancerous cells [82]. In addition, we have found primary cells to be more sensitive to chemical modification than tumor‐derived cell lines. All optimization experiments were performed in primary splenocytes. The spleen is the immune organ of the blood. As such, the white and red pulp contain all major immune cell groups in various states of activation. In addition, the protocol for obtaining them is relatively straight‐forward, fast, and yields high cell numbers. This allowed us to perform multiple experiments in parallel on the same cell population. Splenocytes are obtained by mechanical homogenization of mouse spleens followed by lysis of the red blood cells [83]. The resulting population consists of dendritic cells, macrophages, T‐cells and B‐cells, NK‐cells, neutrophils, iNKT‐cells, and other immune cells in various states of activation. These cells can readily be separated using staining with fluorescent antibodies, followed by flow cytometric identification (see Table S1 for antibodies used). We reasoned that combining antibody‐based identification of different cells with the IEDDA‐quantification of fatty acid uptake, would allow for the facile quantification of uptake of **1–6** at the single cell level and provide information of how the uptake of nutrients varies between cell types, and between cells within a given immune cell population [84].

**FIGURE 3:**
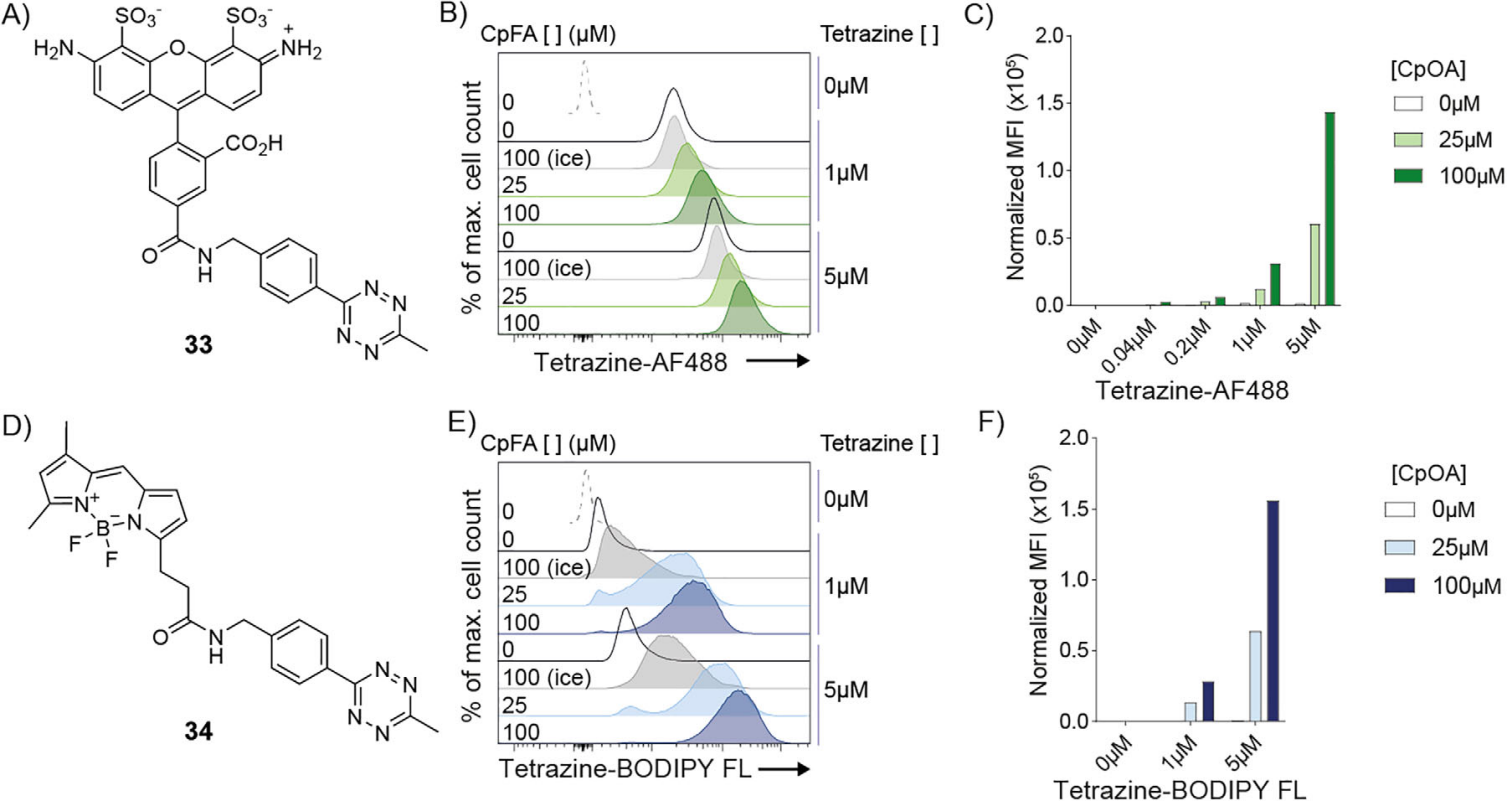
Optimization of ex‐vivo uptake of sterculic acid 4 in mouse splenocytes. Splenocytes were incubated with sterculic acid (0, 25, or 100 µM) for 30 min prior to fixation and subsequent click‐reaction with either AF488‐tz **33** (0.04, 0.2, 1, or 5 µM) or BODIPY‐tetrazine (0, 1, or 5 µM). (A and D) Chemical structures of AF488‐tz **33** and tetrazine‐BODIPY‐FL **34**. (B) Histograms of signal after uptake. Green: 37°C, grey: cold control. (C) Bar chart of the data shown in B. (E) Signal resulting from reaction between **4** after uptake and tetrazine‐BODIPY‐FL **34**. Dark/light blue: 37°C, grey: cold control. (F) Bar chart of data shown in E. All subsequent IEDDA reactions were performed at room temperature. *N* = 1, *n* = 3.

To make the Cp‐FA‐IEDDA uptake protocol compatible with the flow cytometry approach, we first optimized the length of the CpFA pulse using CpOA 4 (Figure S1). Zooming in on our cells of interest, CD4 and CD8 positive T‐cells, pulse lengths >30 min gave a robust signal over background. We deemed these timepoints sufficiently short for the purpose of this study, which was to study early uptake and differences in uptake rates between cells in mixed immune populations. In addition, these timepoints were deemed early enough that protein expression in high/low uptake cells was still unchanged. With these aims in mind, the splenocytes were therefore fixed after the pulse in all the following experiments—despite the live cell compatibility of the IEDDA—as we wanted to prevent any lipid processing and loss of FA during the (long) antibody staining and flow cytometry protocols. This meant that we could also use charged non‐cell permeable fluorophores in the reaction. We, therefore, next assessed which fluorophore gave the best signal‐to‐noise, while retaining compatibility with our antibody‐fluorophore panel. A preliminary experiment in which tetrazine‐modified AF488 (33, AF488‐Tz) or tetrazine‐BODIPY‐FL 34 were compared showed active uptake at 37°C compared to cells that were incubated on ice as a negative controls (Figures 3A,B and S2A). The background of the labeling reaction with AF488‐Tz in the ice‐controls was very high. We postulated this to be due to the absence of quenching properties of the tetrazine to the AF488‐family of dyes upon reaction with these CpFAs [63]. Background and signal went up in parallel with an increases in concentration of 33 (Figure 3B,C). This was not observed for tetrazine‐BODIPY‐FL 34 (Figure 3D–F) [63, 85]. This turn‐on fluorophore is quenched by multiple different mechanisms (FRET, through‐bond energy transfer, photoinduced electron transfer, and others (reviewed in Ref. [86]) and works very well with both live and fixed cells. Using this method, the background of the fluorophore alone was similar to unstained splenocytes (Figure 3D–F) and a FA‐concentration of 25 µM and a concentration of 34 of 1 µM gave a robust uptake signal in all major immune cell populations found in the splenocyte mixture (Figure S2B) and this lipid concentration falls in the physiological concentration range of all six parent lipids [87]. The improved signal‐to‐noise ratio can be quantified as the calculated stain‐index, making the BODIPY‐FL‐Tz 34 highly suitable for flow‐cytometry based assays (Figure S2C). Interestingly, we observed that not all splenocytes became positive when incubated with 25 µM CpOA, suggesting that at this physiological concentration, the competition for nutrient leaves some cells missing out on uptake.

As one of the main aims of this work was to compare the uptake of different fatty acids by immune cells, one factor to consider in these assays is the potential difference in dequenching upon reaction of BODIPY‐FL with each of the CpFAs. We assessed this by performing an in vitro fluorescence turn‐on assay where the six CpFAs (25 µM) were incubated with **34** (1 µM) in PBS for 30 min and the fluorescent signal (*λ*_ex_ = 447 nm, *λ*_em_ = 530 nm) measured (Table S2) to create an adjusted value (relative to linoleic acid). Applying the adjusted values to the intensities of pulsed splenocytes did not alter the overall pattern of uptake across CpFAs (Figure S2E). To assess whether the CpFAs are suitable analogues for their parent FAs, we also carried out a competition assay where CpFAs (25 µM) were simultaneously pulsed with 25, 50, or 100 µM of the parent FA and the reduction in uptake quantified (Figure S2F). Competition of palmitic acid and arachidonic acid were complete (i.e., only 20% of signal remained in presence of 100 µM—a 4‐fold excess of parent lipid). Uptake inhibition of stearic acid, palmitoleic acid, oleic acid and linoleic acid was not complete, with 40%–70% of signal remaining in presence of 100 µM of the parent lipid. These levels of competition are in line with those also observed for alkyne lipids [88], and suggest that either the 1 or 2 atom groups still somewhat affect the biology, or that the uptake mechanisms of these FAs, or that the uptake capacity for these FAs is very high and therefore uninhibited by the high concentration of competing lipid.

### 2.3 CpFA Uptake Properties of Different Mouse Immune Cell Populations

**FIGURE 4:**
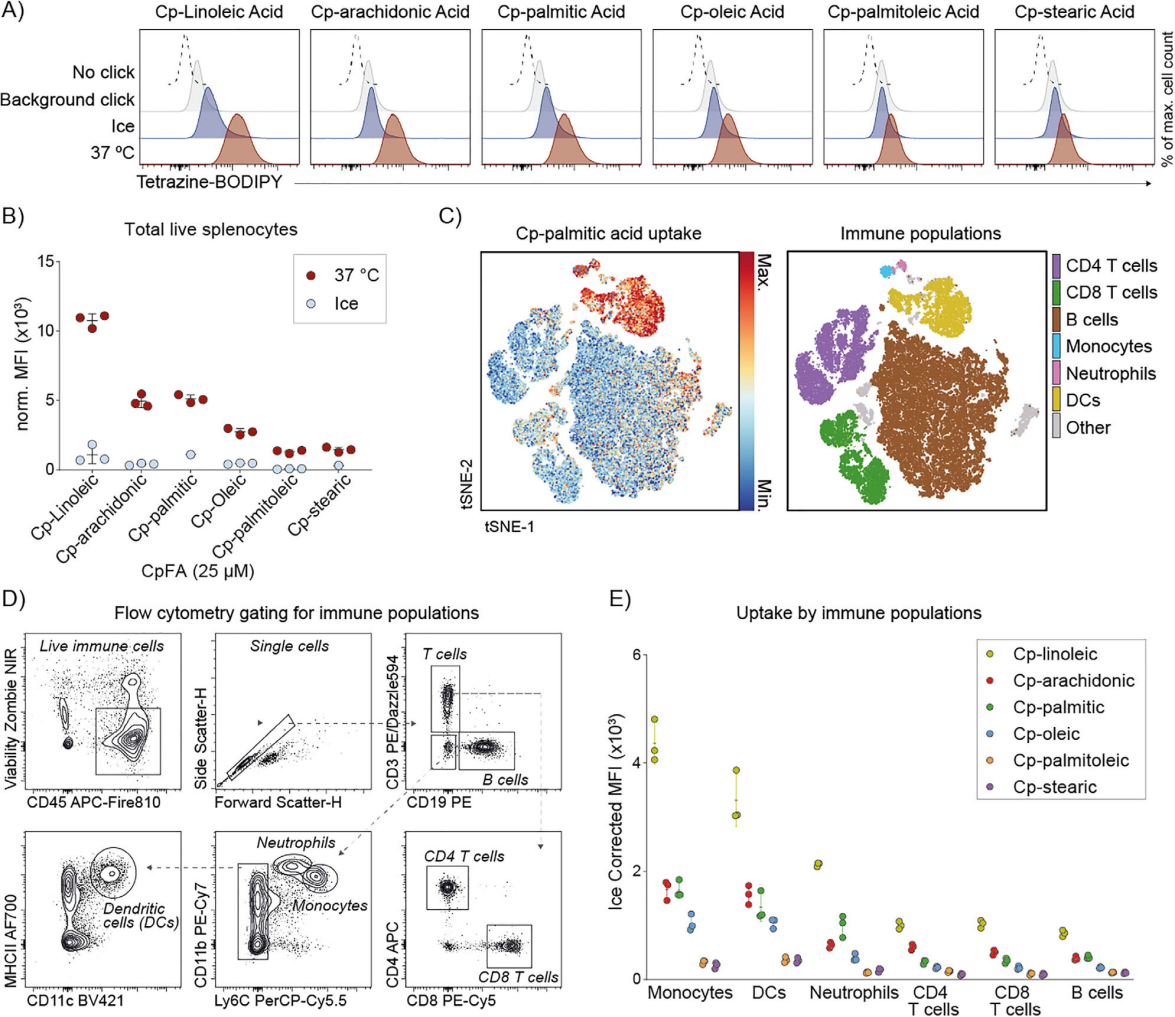
Uptake of CpFA library 1–6 in splenocytes. (A) Representative histograms of the uptake of six CpFAs (37°C) including autofluorescence (no click), non‐specific binding of **34** (background click) and passive uptake (ice). (B) Change in normalized MFI of CpFA (25 µM) uptake with cold control included. (C) Dimensionality reduction using tSNE was performed on splenocytes for Cp‐palmitic acid uptake. Right panel shows splenocytes population including CD19^+^/CD3 B cells, CD11b^+^/Ly6C^hi^ monocytes, Ly6C^hi^ neutrophils, CD3^+^/CD19 CD4^+^ T‐cells, CD3^+^/CD19 CD8^+^ T‐cells, and MHCII^+^/CD11c^+^ dendritic cells. Bottom panel shows the corresponding Cp‐palmitic acid uptake. (D) Corresponding manual gating strategy. (E) Change in for passive uptake corrected MFI of CpFA uptake in different immune cell subsets. Data shown in this figure represents uptake performed in splenocytes harvested from individual mice (*N* = 3) with no technical replicates (*n* = 1).

Having optimized the protocol and validated our CpFAs as analogues for their native counterparts, we aimed to determine how FA were differently taken up across immune populations from the mouse spleen. For this, we applied the optimized protocol to the study of the uptake of all six CpFAs. All lipids gave signal over background at 37°C that was significantly reduced when cells were incubated on ice (Figure 4A,B). None of the CpFAs were toxic to immune cells, as assessed by measuring cell viability in the presence of the CpFAs using a live exclusion dye prior to fixation that is detectable by flow cytometry (Figure S2D). To assess which immune cells take up the various FAs, we performed an unsupervised dimensional reduction to visualize the correlation between immune cell type and FA‐uptake, using palmitic acid as an example (Figure 4C). The resulting *t*‐distributed Stochastic Neighbor Embedding (tSNE) plot indicated particularly high uptake in cells from the myeloid lineage, including monocytes and dendritic cells, whereas lymphoid cells, such as T and B cells, demonstrated comparatively low uptake. We confirmed these findings by manually gating and determining the detection of CpFA for each major immune subset (Figure 4D,E). Notably, all immune cells—despite their different functions and biology—showed a preference for the PUFAs, Cp‐linoleic and Cp‐arachidonic acid, followed by the SFA Cp‐palmitic acid, potentially hinting at shared transporter use between the different cell types analogous to the reliance on the GLUT1 transporter for glucose uptake across immune cell types. Therefore, our library of novel CpFAs has the potential to reveal specific needs for particular fatty acid species across diverse primary immune cell populations.

**FIGURE 5:**
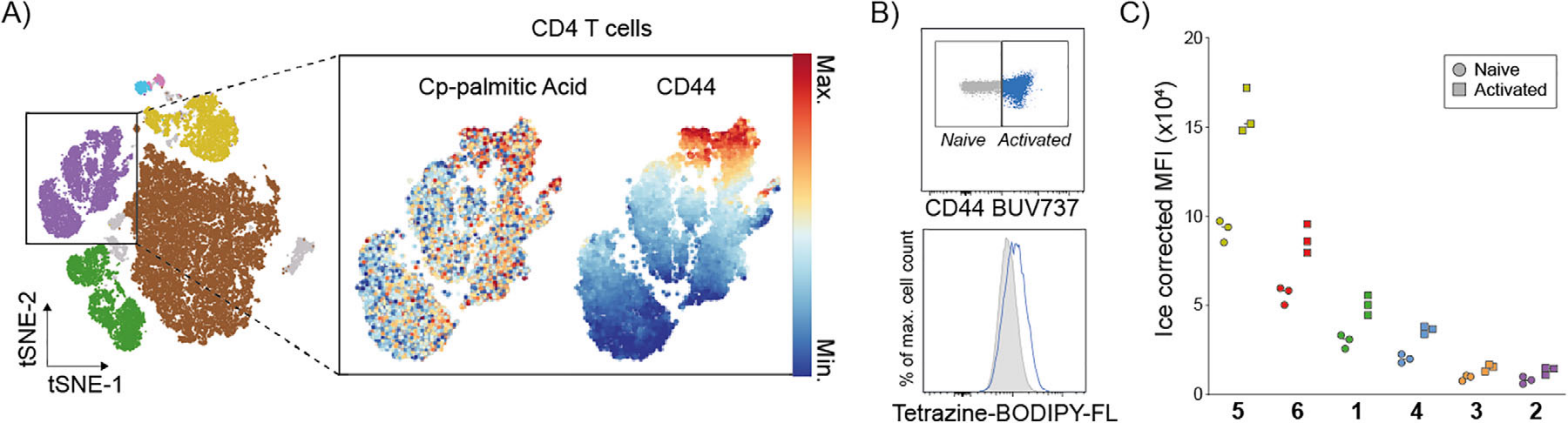
(A) Dimensionality reduction using tSNE on CD4^+^ T‐cells expressing activation marker CD44 for Cp‐palmitic acid uptake. (B) Top panel shows the manual gating strategy for naïve CD44^−^ T‐cells and activated CD44^+^ T‐cells, bottom panel the representative histogram. (C) Difference in corrected MFI of CpFA uptake between naïve and activated T‐cells. Data shown in this figure represents uptake performed in splenocytes harvested from individual mice (*N* = 3) with no technical replicates (*n* = 1).

FA uptake in immune cells is highly regulated during immune cell activation, and can differ within the same cell type depending on their functional state. CD4^+^ T‐cells, for instance, rapidly increase their need for environmental FA when stimulated by the matched antigen to their specific T‐cell receptor, allowing them to divide and produce effector molecules. Expression of the T‐cell activation marker CD44 appeared to correlate with fatty acid uptake (Figure 5A,B). Indeed, all analogues showed an increase in uptake in activated compared to naïve CD4^+^ T‐cells; however, this was less evident for palmitoleic and stearic acid (Figure 5C).

Having seen these differences between activated and naïve T‐cells between the populations, we next wanted to explore whether these tools could be used to determine phenotypic differences within a population. We focused on the naïve and activated T‐cell populations and on the fatty acid oleic acid, due to its established role in T‐cell activation. For this, we opted to study the uptake biology of Cp‐OA **4** in a robust model system of activation, namely the ex vivo activation of T‐cells using α‐CD3/CD28‐coated plastic plates, which provide a mimic of the two key T‐cell activation signals that is known to robustly activate all T‐cells in a mixed splenocyte population [89]. We then incubated the activated and non‐activated T‐cell‐populations with CpFA **4** for 30 min. Rather than a fix‐and‐click protocol used earlier, we here opted to use a live cell IEDDA‐protocol, as we wanted to have the cells alive for respiratory analysis. After washing, the live cells were incubated with fluorophore **34** for one hour and separated into high‐fluorescence and low‐fluorescence subpopulations of T‐cells on the fluorescence‐assisted cell‐sorter (FACS; for gating strategy, see Figure S3). The same difference in FA‐uptake was observed in the live cell assay compared to the fixed cell assay, albeit with a reduced signal‐to‐noise ratio.

**FIGURE 6:**
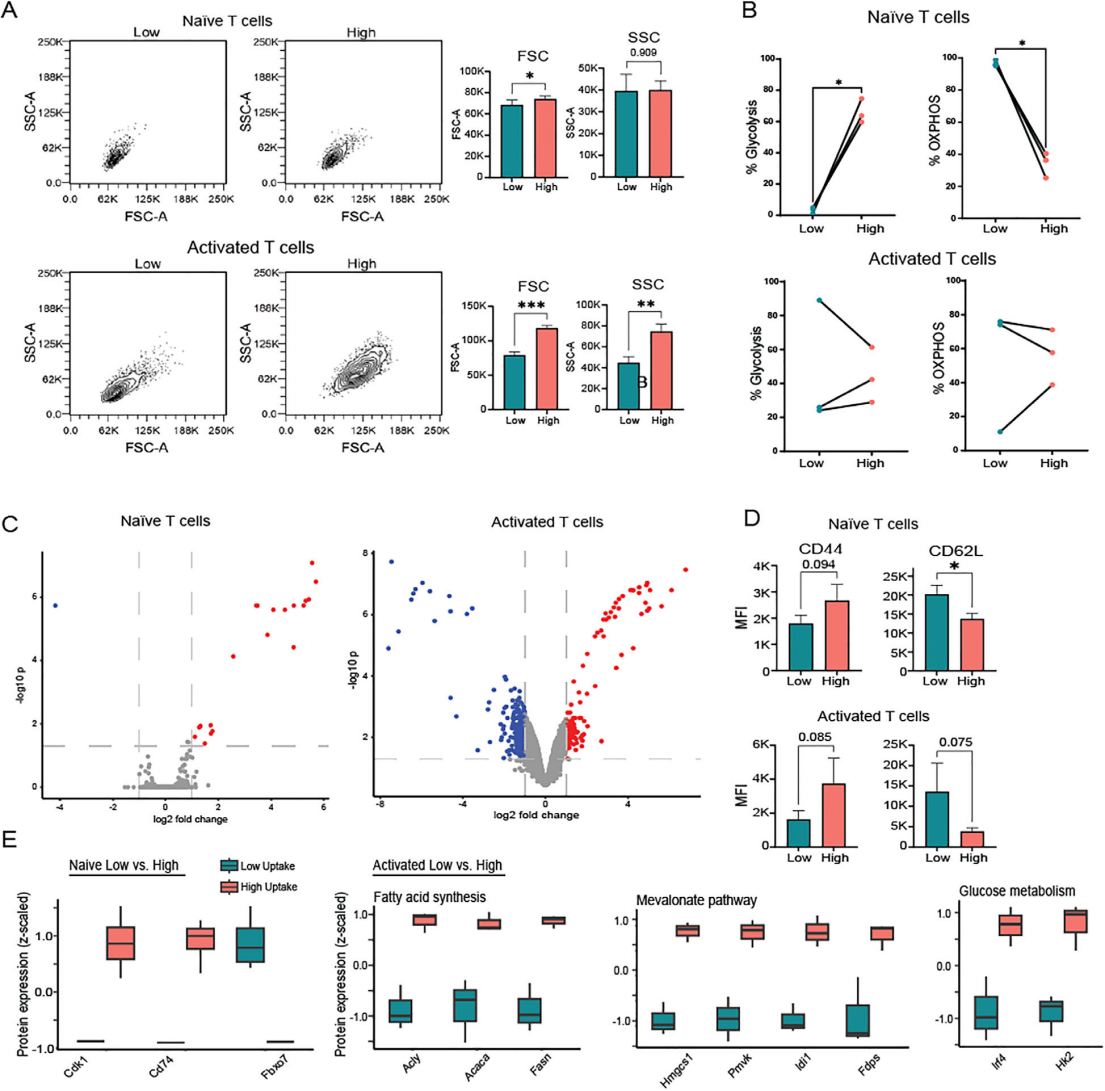
(A) Forward and side scatter plots of high‐ and low‐FA uptake (top and bottom quartiles) of naïve and activated with combined *N* = 3 in the histograms; (B) metabolic flux analysis of the same high/low naïve/activated T‐cells (*N* = 3) with three technical replicates (*n* = 3); (C) proteomic analysis of the same 4 sets of T‐cells (*N* = 5; only proteins found in all samples were included); (D) analysis of activation marker of high/low FA‐uptake subpopulations; (E) quantification of expression levels of key metabolic proteins in the subsets.

After sorting the highest and lowest quartiles from the naïve and active T‐cell populations, we subjected these cells to live‐cell extracellular flux measurement (Figure S3) [90]. These experiments demonstrated that unstimulated low‐FA‐uptake cells rely largely on Oxphos (97%), while the unstimulated high‐uptake cells showing a clear preference for glycolysis (66%). This likely reflects this population consisting of naïve and terminally activated non‐naïve T‐cells. In activated T‐cells there was no significant difference in metabolic reliance between the high/low uptake populations (Figure 6A,B) [91, 92]. Back‐gating the high and low uptake sub‐populations onto the forward and side scatter profiles also showed another effect of increased FA‐uptake: the high uptake activated T‐cells were significantly larger than those that had taken up less FA and were significantly higher for the activation marker CD44 and lower for CD62L (Figure 6A,B). Despite the difference in activation status between the low/high uptake naïve cells, we did not observe significant differences in these early activation markers in the naïve high/low populations. Nor was the size difference large between the two, suggesting that the OA‐high population is of a memory phenotype, as these cells are known to rely on FA‐uptake to fuel their metabolism (Figure 6D) [93, 94].

It was previously shown that high glycolysis in activated T‐cells correlated with increased fatty acid oxidation in an mTOR dependent manner, but it was unknown whether the source of fatty acid was de novo synthesized or taken up from the exogenous environment [94, 95]. In order to assess what pathways underpinned the difference in CpOA‐uptake in these cells, we performed proteomic analysis by mass spectrometry on all four subpopulations (Figure 6C,E). Quantification of the expressed proteomes of the high/low sub‐populations in the naïve populations showed the upregulation of 22 proteins, and downregulation of 1 protein, of which 2 upregulated and the downregulated all related to mTOR activation, supporting the notion that this nutrient sensing mechanism drives changes in uptake [95]. The proteomics of the high/low subpopulations of the activated T‐cells showed bigger changes, despite the lack of differences in respiration. One hundred and fifty‐four proteins were significantly up‐ and 204 were downregulated (Figure 6C), with proteins from the FA biosynthesis, mevalonate and glucose metabolism all significantly altered (Figure 6E), suggesting an interplay between FA uptake and biosynthesis.

## 3 Conclusion

We, here, report the use of a cyclopropene long chain fatty acid analogue library, covering the three main types of saturation, to investigate fatty acid uptake of individual immune cells in complex cell populations. An optimized synthetic methodology was applied to synthesize SFA and MUFA analogues and we developed new synthetic methodology to synthesize the first ever reported cyclopropene PUFA analogues. An initial uptake experiment revealed a high background was detected when using AF488‐Tz due to non‐specific binding. Hence, the quenched tetrazine‐BODIPY‐FL **34** was employed resulting in a much better signal‐to‐noise. The uptake of palmitic acid was visualized in a tSNE plot revealing a higher FA uptake for cells from the myeloid, for instance monocytes and dendritic cells, compared to lymphoid cells like T and B cells. Within all different immune cell types in the spleen a similar pattern was observed where immune cells have a preference for PUFAs, followed by MUFA oleic acid and SFA palmitic acid. We have also shown showed that different cell states (i.e., activated or naïve T‐cells) lead to a quantitative change in exogenous FA uptake in an FA‐specific manner. Interestingly, the uptake of the polyunsaturated FAs, linoleic and arachidonic acid was higher than of all fatty acids in all cell types (although differing widely between cell types). This lends support to the hypothesis of shared uptake mechanisms/transporters between immune cell types, although delineating these patterns requires further investigation.

There are, of course, limitations and caveats to the approach. First of all, the present study only focusses on the uptake of the CpFAs and not their downstream processing and incorporation into other lipid species, lipoproteins and lipid droplets in the cell. Also, the bond angle and flexibility of the Cp compared to the parent alkenes makes the probes still different from the unsaturated fatty acid parent molecules [96]. As for the saturated fatty acid mimics. The competition experiments with the parent nutrients abate these worries. In all, we think CpFAs with the IEDDA is a powerful platform to study relationships between nutrient uptake and immune cell function, as the modifications are tiny and the reaction conditions so mild that the approach can be combined with live cell isolation protocols, proteomics, and respiratory analysis. CpFAs can thus serve as chemically tractable reporters that allow the single cell comparative uptake studies of early FAs in complex immune populations. The fact that the approach can be performed without genetic manipulation of the immune cells under investigation, can be performed on apparatus readily available in most biology labs that can be performed easily by non‐chemists, combined with the ever‐increasing focus on the role of fatty acids (and other nutrients) in cell fate and function, has us believe that the approach could prove of great interest for all experiments in which fatty acid uptake plays a role.

## Conflicts of Interest

The authors declare no conflicts of interest.

## Supporting information



Supporting Figure S1, Tables S1 and S2, and Schemes S1–S4 and experimental information are associated with this manuscript. The authors have cited additional references within the Supporting Information [97, 98, 99, 100, 101, 102, 103, 104, 105, 106, 107, 108, 109, 110, 111, 112, 113]. **Supporting File 1**: anie72785‐sup‐0001‐SuppMat.pdf.

## Data Availability

The data that support the findings of this study are available from the corresponding author upon reasonable request.
