# Supplementary material for: Synthesis of Cyclopropene‐Modified Fatty Acids Allows Single‐Cell Quantification of Uptake by Immune Cells
Source: Angew Chem Int Ed Engl. 2026 May 28;65(31):e25040. doi: 10.1002/anie.202525040 (PMC13411236; doi:10.1002/anie.202525040)

# Synthesis of Cyclopropene-Modified Fatty Acids Allows Single-Cell Quantification of Uptake by Immune Cells


Luuk Reinalda[a], Graham A Heieis[b]#, Laura Bogue Edgerton[a]#,Kristine Bertheussen[a], Diana Torres-García[a], Marouane el Boujadayni[a], Kas Steuten[a], Jeroen M. Punt[c], Wouter P. F. Driever[c], Connor Corrigan[d], Xinyuan Wang[d], Bart Everts[b], David Finlay[d], Mario van der Stelt[c]*, Sander I. van Kasteren[a]*

[a] Dr. L Reinalda, Dr. L.I. Bogue, M. el Boujadayni, Dr. K. Bertheussen, Dr. D. Torres-García, Prof. Dr. S.I. van Kasteren
Division of Chemical Biology and Immunology, Leiden Institute of Chemistry and The Institute for Chemical Immunology,
Leiden University
Leiden, The Netherlands
E-mail: s.i.van.kasteren@chem.leidenuniv.nl, m.van.der.stelt@chem.leidenuniv.nl

[b] Dr. G.A. Heieis, Dr. B. Everts
Department of Parasitology
Leiden University Medical Center
Leiden, The Netherlands

[c] J.M. Punt, W.P.F. Driever, M. van der Stelt
Department of Molecular Physiology
Leiden University
Leiden, The Netherlands

[d] C. Corrigan, X. Wang, Dr. D. Finlay
School of Biochemistry and Immunology, Trinity Biomedical Sciences Institute
Trinity College Dublin
Dublin, Ireland

# These authors have contributed equally.


**Ethical statement**

All mouse experiments were done under license AVD1060020198832 issued after ethical approval by the Centrale Commissie Dierproeven (Den Haag, NL). Spleens were isolated from naïve C57BL/6 mice bred under specific pathogen free (SPF) conditions.

## Table of Contents

Supplementary Schemes and Figures ..... 3
Scheme S1. ..... 3
Scheme S2. ..... 3
Scheme S3. ..... 3
Scheme S4. ..... 4
Figure S1. ..... 4
Figure S2. ..... 5
Figure S3. ..... 6
Supplementary Methods Immunology ..... 7
Isolation of splenocytes ..... 7
Preparation of fatty acid stocks ..... 7
Uptake assay ..... 7
Cell staining and click-reaction for flow cytometry ..... 7
Fluorescence assay ..... 7
Table S1. ..... 8
Table S2. ..... 8
CD3/28 stimulation experiments ..... 8
Flow Cytometry and Sorting. ..... 8
Metabolic Flux Analysis- Seahorse analysis. ..... 9
Proteomics -Sample preparation ..... 9
Data Analysis- Proteomics processing ..... 9
Supplementary Methods Chemistry ..... 10
General information ..... 10
General procedures ..... 10
Synthetic procedures ..... 12
References ..... 25
NMR spectra ..... 26

## Supplementary Schemes and Figures

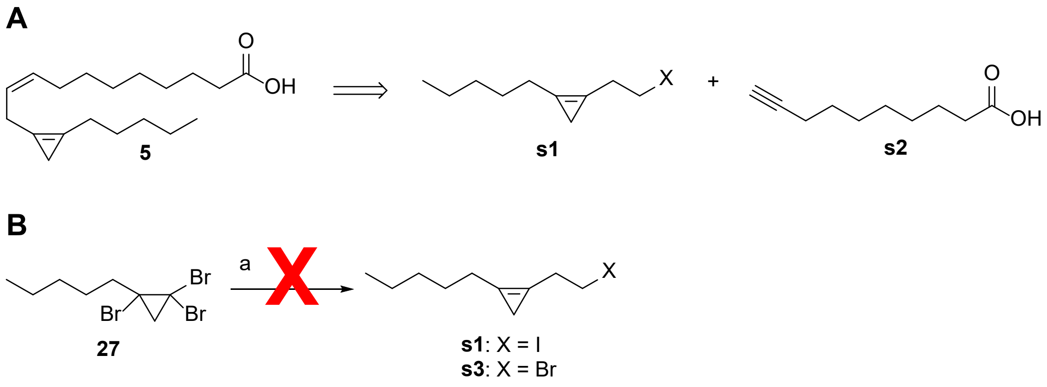


**Scheme S1. Synthetic strategy for PUFA synthesis**. A) Retrosynthetic analysis of compound **5**. B) Attempted route to form cyclopropene halides **s1** or **s3**. Reagents and conditions: a) *n*-Buli, THF, 1,2-diiodoethane or 1-bromo-2-iodoethane, -78 °C to 0 °C, 4 h.

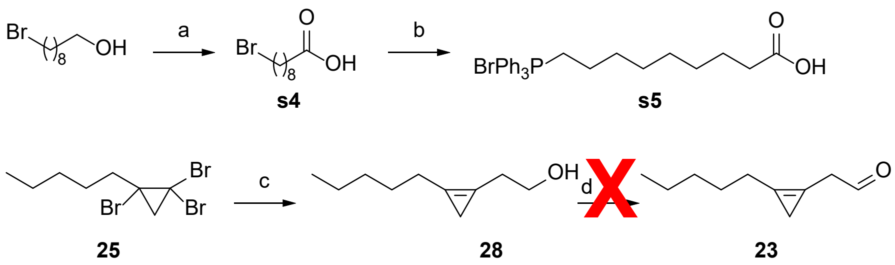


**Scheme S2. Synthetic route for phosphonium bromide s5 and attempted route towards cyclopropene aldehyde 23**. Reagents and conditions: a) TEMPO, BAIB, 2:1 MeCN / water, rt, 4h, 94% yield. b) $PPh_3$, MeCN, reflux, 18 h, quant. yield. c) *n*-Buli, $BF_3{\cdot}OEt_2$, oxirane, THF, -78 °C to 0 °C, 2 h, 49% yield. d) DMP, DCM, 0 °C to rt, 2 h. OR DMSO, $C_2O_2Cl_2$, DCM, $Et_3N$, -78 °C to rt, 2 h. OR DMSO, $Ac_2O$, DCM, rt, 2 h.

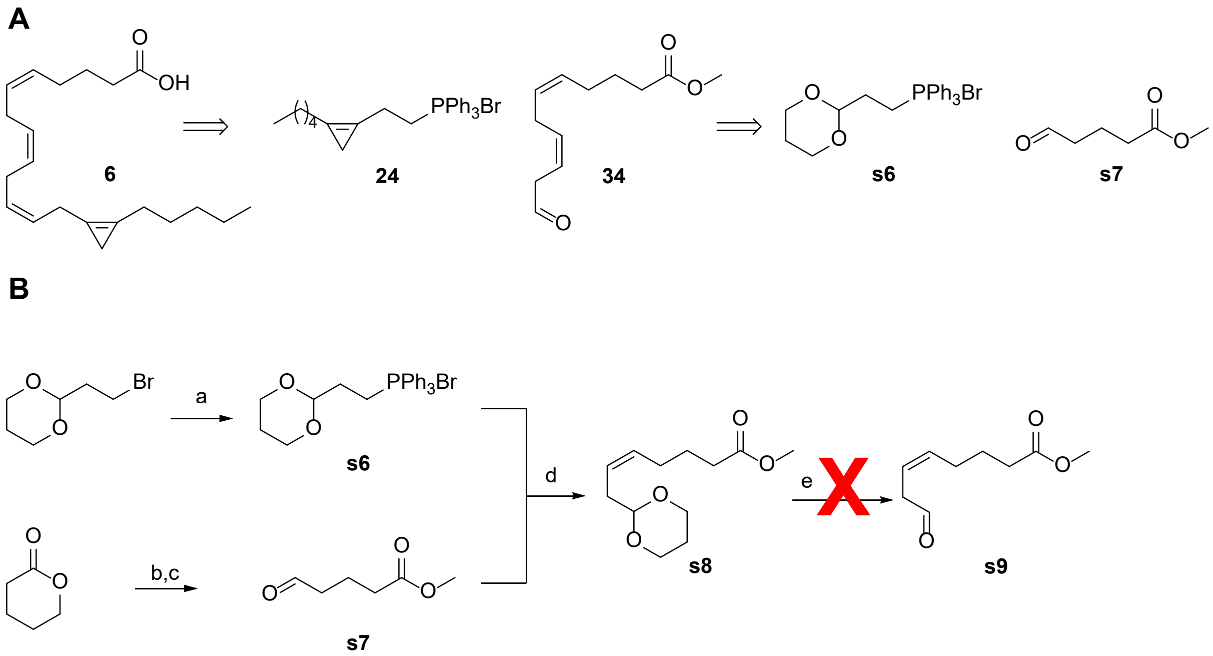


**Scheme S3. Synthetic strategy for PUFA with 3 or more double bonds**. A) Retrosynthetic analysis of fatty acid **6**. B) Reagents and conditions: a) $PPh_3$, MeCN, 82 °C, 18 h, 15% yield. b) $H_2SO_4$, MeOH, reflux, 18 h, 72% yield. c) DMP, DCM, 0 °C to rt, 2 h, 40% yield. d) NaHMDS, THF, -78 °C to rt, 2 h, 65% yield (95% Z-config.). e) HCl (aq.), THF, rt, 20 min OR AcOH, water, 85 °C, 4 h

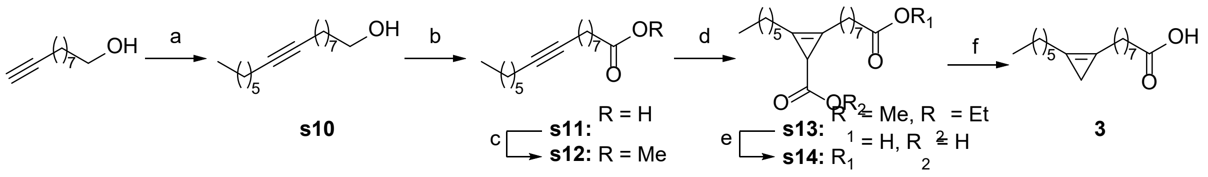


**Scheme S4. Synthesis route for cpPOA 3.** Reagents and conditions: a) *n*-BuLi, 1-bromohexane, NaI, 4:1 THF / HMPA, -78 °C to rt, 18 h, 44% yield. b) TEMPO, BAIB, 2:1 MeCN / water, 0 °C to rt, 2.5 h, 84% yield. c) $SOCl_2$, MeOH, 0 °C, 2.5 h, 88% yield. d) EDA, $Rh_2(OAc)_4$, DCM, 0 °C, 3.5 h, 76% yield. e) KOH, MeOH, reflux, 2.5 h, 97% yield. f) $SOCl_2$, $Et_2O$, 0 °C, 1 h, then $ZnCl_2$, DCM, rt, 3 h, then $NaBH_4$, NaOH, MeOH, -30 °C to rt, 1 h, 10% yield.

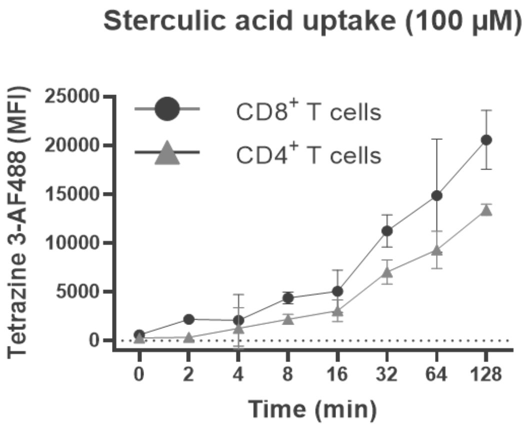


**Figure S1. Timecourse of uptake of sterculic acid by CD4 and CD8 T cells** A) Median fluorescent intensity of Tetrazine-AF488 (5 µM) following click reaction with 100 µM cpFA.

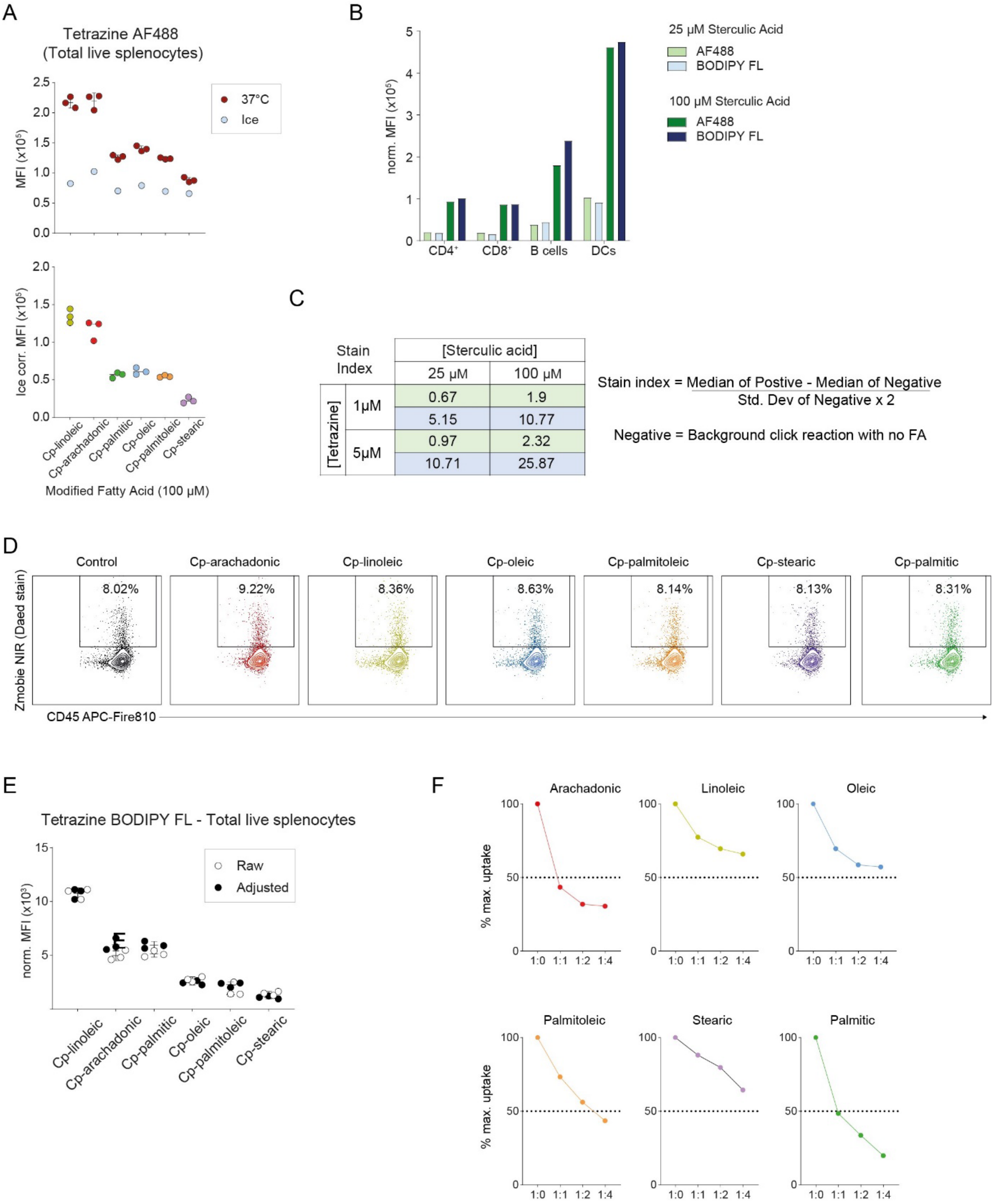


**Figure S2. Optimisation of Cp-FA uptake and click reaction in splenocytes.** A) Median fluorescent intensity of Tetrazine-AF488 (5 µM) following click reaction with 100 µM cpFA pulsed splenocytes (top) and normalized values after subtracting the MFI for splenocytes pulsed on ice (bottom). B) Ice-subtracted MFI of immune populations from the spleen, using 1 µM of either tetrazine-AF488 or tetrazine-BODIPY FL. C) The estimated stain index for tetrazine-AF488 (green) or tetrazine-BODIPY FL (blue), calculated as shown. D) Frequency of dead cells within the CD45+ single cell population, determined by amine dye exclusion and flow cytometry, following cpFA pulse (25 µM). E) Raw and adjusted data for each cpFA according to relative fluorescent intensity in a cell-free assay, set relative to linoleic acid. F) Competition assay showing the relative cpFA uptake with increasing concentration of the corresponding native FA.

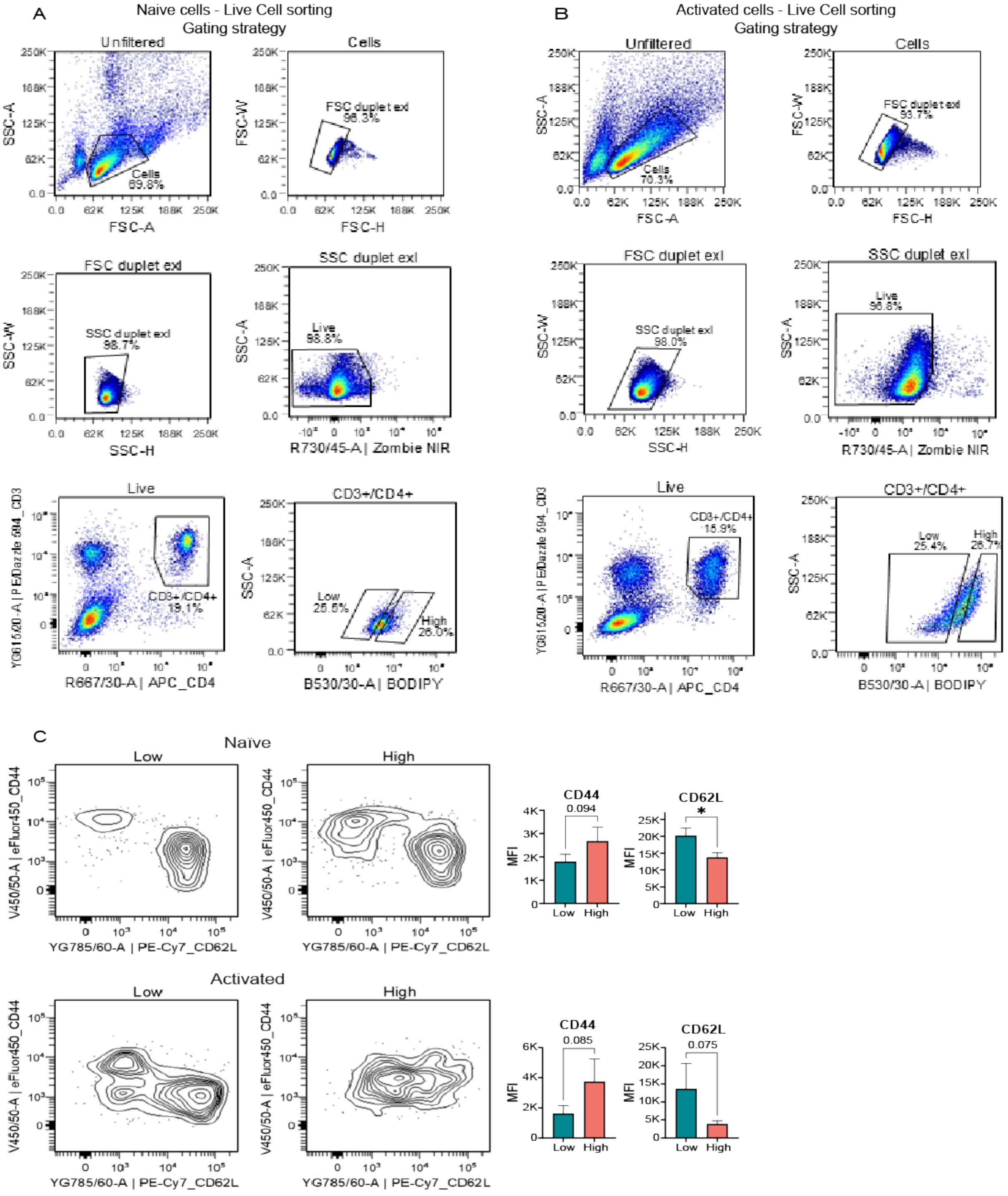


**Figure S3. Gating strategy for live cell IEDDA and subsequent fluorescence assisted cell sorting.** A) Gating strategy for naïve T-cells after live cell IEDDA; B) Gating strategy for activated T-cells after live cell IEDDA; C) staining and quantification for activation markers CD44 and CD62L. Representative samples of a single biological replicate shown. All experiments and quantifications were performed on at least N = 3.

## Supplementary Methods Immunology

### Isolation of splenocytes

All mouse experiments were done under license AVD1060020198832 issued after ethical approval by the Centrale Commissie Dierproeven (Den Haag, NL). Spleens were isolated from naïve C57BL/6 mice bred under specific pathogen free (SPF) conditions. To obtain a single cell suspension, spleens were minced with scissors in a 2 mL Eppendorf and shaken at 37 °C, 200 rpm for 30 minutes in 1 mg/mL of Collagenase IV reconstituted in RPMI medium. Tissue digestion was stopped by adding 1 mL of cold PBS containing 0.5% BSA and 2 mM EDTA and placed on ice. The remaining homogenate was crushed and washed through a 40 µM strainer into a 50 mL tube, spun at 400g for 5 minutes and resuspended in 3 mL of ACK lysis buffer to remove red blood cells. After 5 minutes in lysis solution, 7 mL of cold PBS was added and cells were spun, washed through a second 40 µM strainer and counted for plating using a hemocytometer.

### Preparation of fatty acid stocks

Solid cpFA stocks were first constituted in DMSO to make a 50 mM solution. Two 10x Step-wise dilutions were then done in RPMI medium containing 0.5% fatty acid free BSA to make a 5 mM and subsequent 0.5 mM working stock. The 0.5 mM stock was then diluted to the appropriate 1x concentration to be added to isolated splenocytes.

### Uptake assay

Working FA stocks were first pre-warmed in a 37 °C water bath. 1-2 x $10^6$ splenocytes were plated in a 96-well V-bottom plate and kept on ice. Plated cells were washed twice in 200 µL of cold PBS. After washing, 100 µL of pre-diluted FA stocks (in the appropriate ratio with native FA for the competition experiment) were added directly to the splenocytes, which were immediately re-suspended by gentle pipetting. Resuspended cells were either placed in a 37 °C 5% $CO_2$ incubator or left on ice for 30 minutes, followed by addition of 100 µL cold PBS (0.5% BSA, 2mM EDTA) and spun at 400g for 5 minutes. Cells were washed an additional 2 times with cold PBS and left on ice for cell staining.

### Cell staining and click-reaction for flow cytometry

Following FA uptake, splenocytes were stained in 50 µl of Zombie NIR viability dye diluted 500x and anti-XCR1 antibody in PBS for 15 minutes at room temperature. Cells were then washed 2x with 150 µL of cold PBS (0.5% BSA, 2mM EDTA), fixed with 2% methanol-free formaldehyde for 10 minutes at room temperature, and washed twice with PBS. Cells were subsequently permeabilized by incubation in 50 µL of PBS containing 0.1% saponin and 1% BSA for 15 minutes. Permeabilized cells were washed with PBS and the tetrazine-cyclopropene click reaction was performed by diluting the indicated tetrazine-fluorophore in PBS and incubating in 50 µL at room temperature for 1 hour in the dark. Cells were washed 2 times in PBS and either left in the fridge until staining could be performed or immediately stained with 50 µL of antibody cocktail for the remaining surface markers for 30 minutes, washed twice with PBS, and resuspended in 150 µL PBS for acquisition. A full list of antibodies used can be found in Supplementary Table S1. Fully stained samples were acquired on a Cytek 5-laser Aurora and unmixed using SpectroFlo v3. Analysis was done using FlowJo v10, GraphPad Prism and OMIQ.ai.

### Fluorescence assay

cpFA stocks were diluted to 50 µM in a similar way to the uptake assay. In a black 96-well flat-bottom plate, 100 µL of tetrazine-BODIPY FL (2 µM) was added to 100 µL of cpFA stock (50 µM) and left to react at room temperature for 1 hour. The plate was scanned for fluorescence on a CLARIOstar plate reader (BMG LABTECH) with excitation/emission at 477-14/530- 40 and dichroic filter 497 showing

the turn-on ratio between the sample and negative control (1 µM tetrazine-BODIPY FL), as an average of the three samples.

**Table S1.** Flow cytometry reagents.

| Marker | Conjugate | Dilution | Supplier | Cat. # |
|---|---|---|---|---|
| CD45 | APC-Fire810 | 1000x | BioLegend | 103174 |
| CD3 | PE/Dazzle594 | 1000x | BioLegend | 100246 |
| CD4 | APC | 1000x | ThermoFisher | 17-0042-83 |
| CD44 | BUV737 | 1000x | BD Biosciences | 612799 |
| CD8 | PE-Cy5 | 1000x | ThermoFisher | 15-0081-82 |
| CD19 | PE | 200x | ThermoFisher | 12-0191-82 |
| MHCII | AF700 | 400x | ThermoFisher | 56-5321-82 |
| CD11c | BV421 | 400x | BioLegend | 117330 |
| XCR1 | BV650 | 400x | BioLegend | 148220 |
| CD172a | BUV805 | 200x | BD Biosciences | 741997 |
| CD11b | PE-Cy7 | 10000x | ThermoFisher | 25-0112-82 |
| Ly6C | PerCP-Cy5.5 | 1000x | BioLegend | 128028 |

**Table S2.** Turn-on Ratio of cpFAs in fluorescence assay with tetrazine-BODIPY FL.

| cpFA | Turn-on Ratio | Relative ToR |
|---|---|---|
| Cp-palmitic acid | 4.9 | 0.56 |
| Cp-stearic acid | 1.8 | 1.58 |
| Cp-palmitoleic acid | 5.3 | 0.52 |
| Cp-oleic acid | 2.2 | 1.29 |
| Cp-linoleic acid | 2.8 | 1 |
| Cp-arachidonic acid | 3.2 | 0.88 |

**CD3/28 stimulation experiments**

Spleens were collected from naïve C57BL/6 mice, mechanically dissociated through a 70 µm cell strainer using the flat end of a syringe plunger, and washed with single-cell suspension buffer (SCSB; PBS containing 2% FCS and 1 mM EDTA) until no visible tissue fragments remained. Cells were pelleted (300 rcf, 10 min, 4 °C), resuspended in 2 mL ACK lysis buffer (Thermo Fisher Scientific) per spleen for erythrocyte removal (5 min), diluted with cold PBS (40 mL), and pelleted (500 rcf, 5 min, 4 °C). The pellet was filtered through a 40 µm cell strainer and resuspended in splenocyte medium [RPMI 1640 (Capricorn Scientific) supplemented with 10% FCS, 2 mM GlutaMAX, 100 IU $mL^{-1}$ penicillin, 50 µg $mL^{-1}$ streptomycin, and 50 µM 2-mercaptoethanol]. Cell number was determined by hemocytometer.

For stimulation, Nunc™-treated 6-well plates were coated with Ultra-LEAF purified anti-mouse CD3ε (BioLegend, 5 µg $mL^{-1}$, 2 h, 37 °C), washed, and seeded with $1.5 \times 10^7$ splenocytes per well in 5 mL splenocyte medium containing anti-CD28 (3 µg $mL^{-1}$) and recombinant murine IL-2 (PeproTech, 10 U $mL^{-1}$). Unstimulated controls received IL-2 only. Cultures were incubated overnight (37 °C, 5% $CO_2$), harvested by scraping, pelleted (300 rcf, 10 min), and resuspended in fresh medium prior to downstream use.

**Flow Cytometry and Sorting- Fluorescent labeling and sorting of naïve and activated splenocytes.**

Splenocytes from three spleens (~4–5 × $10^8$ cells) were preincubated with sterculic acid (100 µM, 15 min, 37 °C), washed with medium and PBS, then labeled with fluorophore 7 (1 µM, 35 min, ice). Cells

were washed twice with PBS and stained for viability [Zombie NIR (BioLegend, 1:500) and DNase I (Thermo Fisher Scientific, 30 U $mL^{-1}$) in HBSS, 15 min, RT]. Antibodies were added as follows: PE/Dazzle 594 anti-CD3 (BioLegend; naïve: 1:800, activated: 1:200), APC anti-CD4 (1:400), eFluor450 anti-CD44 (1:200), PE-Cy7 anti-CD62L (1:1000); incubation: 25 min, RT. After washing with FACS buffer (PBS + 0.2% BSA, 2 mM EDTA), samples were sorted on a BD FACS Aria III using the gating strategies in Figures S3A (naïve) and B (activated). Biological triplicates were performed.

**Metabolic Flux Analysis- Seahorse analysis**

Sorted high- and low-uptake cells (1–2 × $10^6$) were equilibrated in XF assay medium + 5 mM D-(+)-glucose (37 °C, 1 h, non-$CO_2$). The Seahorse XFe24 analyzer (Agilent Technologies) measured basal OCR and ECAR, followed by sequential injection of oligomycin A (1.8 µM) and antimycin A (2 µM; both Sigma-Aldrich). Data was normalized to cell number, analyzed by repeated-measures ANOVA, and visualized as bar and scatter plots using Agilent software.

**Proteomics -Sample preparation**

Sorted cells (2 × $10^5$ per population) were pelleted (300 rcf, 5 min, 4 °C), flash-frozen, and stored at −80 °C. Pellets were lysed [PBS + 250 mM sucrose, 5 mM EDTA, cOmplete™ protease inhibitor (Roche)], sonicated (Qsonica Q700, 4 × 10 s, 10% amplitude, 0 °C), and quantified (~4 µg protein by Qubit; Invitrogen). Proteins were acetone-precipitated (−80 °C, 1 h + overnight), reduced (5 mM DTT, 15 min, 65 °C), alkylated (10 mM IAA, 30 min, RT, dark), quenched (7 mM DTT), diluted (<1 M urea), and trypsin-digested (Promega, 0.2 µg, overnight, 37 °C). Peptides were desalted (Empore™ C18 StageTips), dried (SpeedVac), and reconstituted in 97:3:0.05 $H_2O/CH_3CN$/TFA.

LC–MS/MS. Peptides were separated on a PepSep C18 column (25 cm × 75 µm, 1.5 µm; Bruker) using a nanoElute 2 system (Bruker Daltonics; 2–95% B gradient, 300 nL $min^{-1}$; A: 0.1% FA, B: 0.1% FA/99.9% $CH_3CN$) coupled to a timsTOF HT (DIA-PASEF mode; m/z 300–1300, $1/K_0$ 0.7–1.35 Vs $cm^{-2}$). Calibration used Agilent ESI Tuning Mix and sodium formate.

**Data Analysis- Proteomics processing**

DIA-NN v1.8.1 analyzed raw files against the UniProt mouse proteome (UP000000589; 17 March 2024). R (v4.3.x) processed report.unique_genes_matrix.tsv files: missing values were imputed (≤1 per group averaged; all-missing set to trace level). Limma v3.58.1 identified significant proteins (≥2-fold change, adj. $p < 0.05$; empirical Bayes). Pathways were enriched with clusterProfiler v4.8.3.

## Supplementary Methods Chemistry

### General information

All commercially available reagents and solvents were used as received unless stated otherwise. Anhydrous solvents were prepared by storage over activated 3 Å (only for MeOH) or 4 Å molecular sieves. Reaction progress was determined with thin layer chromatography (TLC) (Sigma, TLC Silica gel 60 F254) via UV visualisation (λ = 254 nm) and potassium permanganate stain (6 g $KMnO_4$ and 40 g, $K_2CO_3$ in 600 mL water). All column chromatography purifications were performed using solvents as received and silica gel (Macherey-Nagel, Kieselgel 60 M, 0.04 – 0.63 mm). Analytical LC-MS analysis was performed on a Finnigan Surveyor HPLC system (detection at 200-600 nm) with an analytical C18 column (Gemini, 50 x 4.6 mm, 3 µm particle size, Phenomenex) coupled to a Thermo LCQ Fleet Ion mass spectrometer (ESI+). Solvent system: A: water (gradient, 10-90%), B: $CH_3CN$ (gradient, 90-10%), C: (constant, 10%) 1% TFA (aq). Recorded data was interpreted and analysed using Xcalibur software. $^{1}H$ and $^{13}C$ NMR spectra were recorded using a Brüker AV-400 (400/101 MHz) or AV-500 (500/126 MHz). Chemical shift values are reported in ppm (δ) relative to the residual solvent signal of $CDCl_3$ (δ = 7.26 ppm for $^{1}H$ and δ = 77.16 ppm for $^{13}C$ NMR). Multiplicities are given as singlet (s), doublet (d), triplet (t), quartet (q), pentet (p), doublet of doublets (dd), broad singlet (br s) and multiplet (m). Recorded data was interpreted and analysed using MestReNova software.

### General procedure A: Oxidation of alcohol to carboxylic acid

(Diacetoxyiodo)benzene (3 eq) and TEMPO (0.3 eq) were added to a solution of the alcohol (1. eq) in 2:1 MeCN / water (0.2 M) at 0 °C and stirred for 3 h at rt. The solution was quenched with sat. aq. $Na_2S_2O_3$, diluted with water and extracted with EtOAc (3x). The combined organic layers were washed with brine, dried over $MgSO_4$ and concentrated under reduced pressure. The crude product was purified by silica gel column chromatography to afford the carboxylic acid.

### General procedure B: Hydrobromination of alkyne

In a slightly modified procedure from the literature[1], terminal alkyne (1 eq) was added dropwise to a diluted solution of $BBr_3$ (0.5 M in anh. DCM) (0.5 eq) at -78 °C under $N_2$ atmosphere and the reaction was slowly warmed to rt and stirred for 3 hours. The solution was cooled to 0 °C and acetic acid (30 eq) was added and stirred for another 1 h. The solution was neutralized carefully with sat. aq. $NaHCO_3$ and extracted with DCM (3x). The combined organic layers were washed with brine, dried over $MgSO_4$ and concentrated under reduced pressure (900 mbar). The crude product was purified by silica gel column chromatography (pentane) to afford 2-bromoalkene.

### General procedure C: Cyclopropanation of 2-bromoalkene

In a slightly modified procedure from the literature[2], a sat. aq. NaOH solution (5 eq) was added dropwise to a solution of 2-bromo alkene (1 eq) and cetyltrimethylammonium bromide (0.1 eq) in bromoform (3 eq) and DCM (1.0 M) at 0 °C and stirred for 18 h. The solution was diluted with water and extracted with DCM (3x). The combined organic layers were washed with brine, dried over $MgSO_4$ and concentrated under reduced pressure. The crude product was purified by silica gel column chromatography (pentane) to afford the tribromocyclopropane.

**General procedure D: Substiution of tribromocyclopropane on diiodide**

In a slightly modified procedure from the literature[3], a solution of *n*-butyllithium (2.2 eq) was added dropwise to a solution of tribromocyclopropane (1 eq) in anhydrous THF (0.25 M) at -78 °C under $N_2$ atmosphere and was slowly warmed to 0 °C and stirred for 1 hour. A solution of diiodoalkane (2 eq) in anhydrous THF (1.0 M) was added and the reaction was stirred for 3 h at rt. The reaction was quenched with water, extracted with 1:1 $Et_2O$ / pentane (3x). The combined organic layers were washed with brine, dried over $MgSO_4$ and concentrated under reduced pressure. The crude product was purified by silica gel column chromatography (pentane) to afford the iodocyclopropene.

**General procedure E: Substitution of nitrile on iodocycloprpene**

In a slightly modified procedure from the literature[1], NaCN (2 eq) was added in one portion to a solution of iodocyclopropene (1 eq) in anhydrous DMSO (0.2 M) under $N_2$ atmosphere and stirred at 90 °C for 1 h. The reaction was diluted with water and extracted with $Et_2O$ (3x). The combined organic layers were washed with brine, dried over $MgSO_4$ and concentrated under reduced pressure. The crude product was purified by silica gel column chromatography ($Et_2O$/pentane 1:9) to afford the nitrilecyclopropene.

**General procedure F: Hydrolysis of nitrilecyclopropene**

In a slightly modified procedure from the literature[1], a sat. aq. NaOH solution (10 eq) was added to a solution of nitrilecyclopropene (1 eq) in 2:1 EtOH / water (0.2 M) and refluxed overnight. The reaction was acidified with aq. 1 M HCl and extracted with $Et_2O$ (3x). The combined organic layers were washed with brine, dried over $MgSO_4$ and concentrated under reduced pressure. The crude product was purified by silica gel column chromatography ($Et_2O$/pentane 1:9, 1% AcOH) to afford the cyclopropene fatty acid.

**General procedure G: Synthesis of phosphonium bromides**

In a slightly modified procedure from the literature[4], triphenylphosphine (1.1 eq) was added to a solution of alkylbromide (1 eq) in anh. MeCN at rt under $N_2$ atmosphere and the reaction mixture was stirred for 18 h at 100 °C. The solvents were removed under reduced pressure and the crude product was purified by silica gel column chromatography (DCM/MeOH 99:1 to 19:1) to afford the phosphonium bromide.

**General procedure H: Hydrolysis of cyclopropene fatty acid methyl ester**

An aq. 2 M NaOH solution (5 eq) was added to a solution of protected cyclopropene fatty acid methyl ester (1 eq) in 1:1 THF / EtOH (0.2 M) and stirred at 70 °C for 2 h. The reaction mixture was acidified with aq. 1 M HCl and extracted with $Et_2O$ (3x). The combined organic layers were washed with brine, dried over $MgSO_4$ and concentrated under reduced pressure. The crude product was purified by silica gel column chromatography ($Et_2O$/pentane 1:9, 1% AcOH) to afford the cyclopropene fatty acid.

**Synthetic procedures**

**2-bromohept-1-ene (10)**

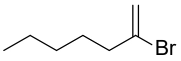


General procedure B was used with hept-1-yne (7.33 g, 76.22 mmol, 1.0 eq) as starting material to afford the title compound **10** (7.05 g, 39.81 mmol, 52% yield) as a colorless liquid. $^1$H NMR (400 MHz, $CDCl_3$) δ 5.55 (dt, *J* = 1.6, 1.2 Hz, 1H), 5.38 (d, *J* = 1.6 Hz, 1H), 2.41 (td, *J* = 7.4, 1.2 Hz, 2H), 1.65 – 1.48 (m, 2H), 1.39 – 1.15 (m, 4H), 0.90 (t, *J* = 7.0 Hz, 3H). Spectral data was in accordance with the literature.[9]

**1,1,2-tribromo-2-pentylcyclopropane (11)**

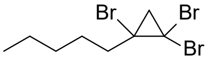


General procedure C was used with compound **10** (7.05 g, 39.81 mmol, 1.0 eq) as starting material to afford the title compound **11** (7.88 g, 22.57 mmol, 57% yield) as a colorless liquid. $^1$H NMR (400 MHz, $CDCl_3$) δ 2.13 – 1.99 (m, 2H), 1.98 (dd, *J* = 9.1, 0.8 Hz, 1H), 1.85 (d, *J* = 9.2 Hz, 1H), 1.82 – 1.58 (m, 2H), 1.48 – 1.29 (m, 4H), 1.10 – 0.84 (m, 3H). Spectral data was in accordance with the literature.[10]

**2-(2-pentylcycloprop-1-en-1-yl)ethan-1-ol (12)**

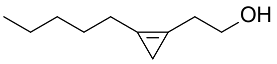


A solution of *n*-Buli (1.6 M in hexanes, 7.80 mL, 12.48 mmol, 2.2 eq) was added dropwise to a solution of compound **11** (1.98 g, 5.67 mmol, 1 eq) at -78 °C under $N_2$ atmosphere and stirred for 30 min. A solution of oxirane (2.5 – 3.3 M in THF, 4.54 mL, 11.35 mmol, 2 eq) and BF3·OEt (7.8 ml, 12.48 mmol, 2.2 eq) were added carefully and the reaction mixture was stirred for 15 min at -78 °C and for 1 h at 0 °C. The reaction was quenched with sat. aq. $NH_4Cl$ and extracted with $Et_2O$ (3x). The combined organic layers were washed with brine, dried over $MgSO_4$ and concentrated under reduced pressure. The crude product was purified by silica gel column chromatography ($Et_2O$ / pentane 1:9 to 1:4) to afford compound **12** (428 mg, 2.77 mmol, 49% yield) as a light yellow oil. **$^1$H NMR** (400 MHz, $CDCl_3$) δ 3.84 (t, *J* = 6.3 Hz, 2H), 2.68 (tt, *J* = 6.2, 1.6 Hz, 2H), 2.41 (tt, *J* = 7.3, 1.6 Hz, 2H), 1.61 – 1.52 (m, 2H), 1.50 (br s, 1H), 1.41 – 1.23 (m, 4H), 0.95 – 0.86 (m, 3H), 0.84 (s, 2H).

**1-(2-bromoethyl)-2-pentylcycloprop-1-ene (13)**

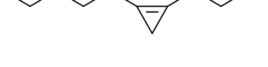


N-bromosuccinimide (506 mg, 3.28 mmol, 1 eq) and triphenylphosphine (1.03 mg, 3.94 mmol, 1.2 eq) were added in one portion to a solution of compound **12** (700 mg, 3.94 mmol, 1.2 eq) in anh. DCM at 0 °C under $N_2$ atmosphere and stirred at rt for 1 h. The reaction mixture was quenched with sat. aq. $NaHCO_3$ and extracted with DCM (3x). The combined organic layers were washed with brine, dried over $MgSO_4$ and concentrated under reduced pressure. The crude product was purified by silica gel column chromatography (pentane) to afford compound **13** (417 mg, 1.92 mmol, 59% yield) as a light yellow oil. **$^1$H NMR** (400 MHz, $CDCl_3$) δ 3.54 (t, *J* = 7.0 Hz, 2H), 2.99 (tt, *J* = 7.1, 1.6 Hz, 2H), 2.42 (tt, *J* =

7.3, 1.5 Hz, 2H), 1.62 – 1.51 (m, 2H), 1.39 – 1.21 (m, 4H), 0.93 – 0.87 (m, 3H), 0.86 (s, 2H). **$^{13}$C NMR** (101 MHz, $CDCl_3$) δ 112.9, 106.7, 31.7, 30.2, 30.2, 27.0, 26.2, 22.6, 14.2, 7.6.

**(2-(2-pentylcycloprop-1-en-1-yl)ethyl)triphenylphosphonium bromide (7)**

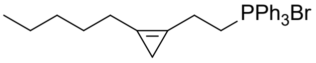


General procedure G was used with compound **13** (49 mg, 0.23 mmol, 1.0 eq) as starting material to afford the title compound **7** (89 mg, 0.19 mmol, 82% yield) as a light yellow oil. **$^{1}$H NMR** (400 MHz, $CDCl_3$) δ 7.99 – 7.64 (m, 15H), 3.96 (dt, *J* = 12.1, 7.0 Hz, 2H), 3.00 (dtt, *J* = 17.4, 7.1, 1.5 Hz, 2H), 2.23 (tt, *J* = 7.3, 1.6 Hz, 2H), 1.47 – 1.35 (m, 2H), 1.35 – 1.14 (m, 4H), 0.85 (t, *J* = 7.0 Hz, 3H), 0.60 (s, 2H). **$^{13}$C NMR (**101 MHz, $CDCl_3$) δ 135.3, 135.2, 133.6, 133.5, 130.6, 130.5, 118.2, 117.3, 113.5, 105.7, 105.6, 31.4, 26.7, 25.5, 22.3, 21.5, 21.0, 19.5, 19.5, 14.0, 7.9.

**9-((tert-butyldimethylsilyl)oxy)nonan-1-ol (14)**

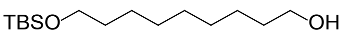


Imidazole (510 mg, 7.49 mmol, 1.2 eq) and TBSCl (940 mg, 6.24 mmol, 1 eq) were added to a solution of nonane-1,9-diol (2.0 g, 12.48 mmol, 2 eq) in anh. DCM at rt under $N_2$ atmosphere and stirred for 3 h. The reaction mixture was diluted with water and extracted with DCM (3x). The combined organic layers were washed with brine, dried over $MgSO_4$ and concentrated under reduced pressure. The crude product was purified by silica gel column chromatography ($Et_2O$ / pentane 1:4 to 1:2) to afford compound **14** (1.10 g, 4.0 mmol, 64% yield) as a clear oil. **$^{1}$H NMR** (400 MHz, $CDCl_3$) δ 3.64 (td, *J* = 6.7, 5.2 Hz, 2H), 3.59 (t, *J* = 6.6 Hz, 2H), 1.64 – 1.44 (m, 4H), 1.40 – 1.27 (m, 10H), 0.89 (s, 9H), 0.04 (s, 6H). $^{1}$H NMR (400 MHz, $CDCl_3$) δ 3.63 (t, *J* = 6.6 Hz, 2H), 3.59 (t, *J* = 6.6 Hz, 2H), 1.61 – 1.44 (m, 4H), 1.37 – 1.22 (m, 10H), 0.89 (s, 9H), 0.04 (s, 6H). **$^{13}$C NMR** (101 MHz, $CDCl_3$) δ 63.5, 63.2, 33.0, 32.9, 29.7, 29.5, 26.1, 25.9, 25.9, 18.5, -5.1. Spectral data was in accordance with the literature.[11]

**9-((tert-butyldimethylsilyl)oxy)nonanoic acid (15)**

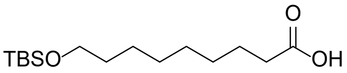


General procedure A was used with compound **14** (1.16 g, 4.02 mmol, 1.0 eq) as starting material, the crude product was purified by silica gel column chromatography ($Et_2O$ / pentane 1:4, 1% AcOH) to afford compound **15** (1.07 g, 3.70 mmol, 92% yield) as a light yellow oil. **$^{1}$H NMR** (400 MHz, $CDCl_3$) δ 3.59 (t, *J* = 6.6 Hz, 2H), 2.34 (t, *J* = 7.5 Hz, 2H), 1.63 (p, *J* = 7.5 Hz, 2H), 1.54 – 1.45 (m, 2H), 1.38 – 1.24 (m, 8H), 0.89 (s, 9H), 0.04 (s, 6H). **$^{13}$C NMR** (101 MHz, $CDCl_3$) δ 180.3, 63.4, 34.2, 33.0, 29.4, 29.1, 26.1, 25.9, 24.8, 18.5, -5.1. Spectral data was in accordance with the literature.[11]

**methyl 9-((tert-butyldimethylsilyl)oxy)nonanoate (16)**

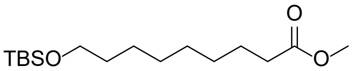

Methyl iodide (0.43 mL, 6.93 mmol, 2 eq) and $K_2CO_3$ (1.44 g, 10.4 mmol, 3 eq) were added to a solution of compound **15** (1.0 g, 3.47 mmol, 1 eq) in anh. DMF at rt under $N_2$ atmosphere and stirred for 18 h. The reaction mixture was diluted (10x) with sat. aq. $NaHCO_3$ and extracted with $Et_2O$ (3x). The combined organic layers were washed with brine, dried over $MgSO_4$ and solvents removed under reduced pressure. The crude product was purified by silica gel column chromatography ($Et_2O$ / pentane 1:9) to afford compound **16** (898 mg, 2.97 mmol, 86% yield). **$^1$H NMR** (400 MHz, $CDCl_3$) δ 3.66 (s, 3H), 3.59 (t, *J* = 6.6 Hz, 2H), 2.30 (t, *J* = 7.6 Hz, 2H), 1.70 – 1.56 (m, 2H), 1.55 – 1.43 (m, 2H), 1.35 – 1.23 (m, 8H), 0.89 (s, 9H), 0.04 (s, 6H). **$^{13}$C NMR** (101 MHz, $CDCl_3$) δ 174.5, 63.4, 51.6, 34.2, 33.0, 29.4, 29.2, 26.1, 25.9, 25.1, 18.5, -5.1. Spectral data was in accordance with the literature.[12]

**methyl 9-hydroxynonanoate (17)**

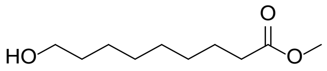


A solution of TBAF (1.0 M in THF, 8.73 mL, 8.73 mmol, 3 eq) was added dropwise to a solution of compound **16** (880 mg, 2.91 mmol, 1 eq) in anh. THF (0.3 M) at 0 °C under $N_2$ atmosphere and stirred for 5 h at rt. The reaction mixture was diluted with aq. 1 M HCl and extracted with DCM (3x). The combined organic layers were washed with brine, dried over $MgSO_4$ and solvents removed under reduced pressure. The crude product was purified by silica gel column chromatography (EtOAc/pentane 1:4 to 1:1) to afford compound **17** (512 mg, 2.72 mmol, 93% yield) **$^1$H NMR** (400 MHz, $CDCl_3$) δ 3.65 (s, 3H), 3.61 (t, *J* = 6.6 Hz, 2H), 2.28 (t, *J* = 7.5 Hz, 2H), 1.68 – 1.47 (m, 4H), 1.38 – 1.23 (m, 8H). **$^{13}$C NMR** (101 MHz, $CDCl_3$) δ 174.5, 63.1, 51.6, 34.2, 32.8, 29.3, 29.1, 25.8, 25.0. Spectral data was in accordance with the literature. [13]

**methyl 9-oxononanoate (8)**

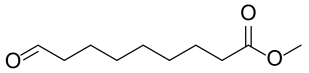


Dess-Martin periodinane (1.41 g, 3.32 mmol, 1.3 eq) was added to a solution of compound **17** (481 mg, 2.55 mmol, 1 eq) in anh. DCM (0.2 M) at 0 °C under $N_2$ atmosphere and stirred for 1 h at rt. The reaction mixture was quenched with sat. aq. $NaHCO_3$ and extracted with $Et_2O$ (3x). The combined organic layers were washed with brine, dried over $MgSO_4$ and solvents removed under reduced pressure. The crude product was purified by silica gel column chromatography ($Et_2O$ / pentane 1:4) to afford compound **8** (372 mg, 2.0 mmol, 78% yield) as a light yellow oil. **$^1$H NMR** (400 MHz, $CDCl_3$) δ 9.75 (t, *J* = 1.6, 1H), 3.65 (s, 3H), 2.41 (td, *J* = 7.3, 1.8 Hz, 2H), 2.29 (t, *J* = 7.5 Hz, 2H), 1.70 – 1.52 (m, 4H), 1.39 – 1.22 (m, 6H). **$^{13}$C NMR** (101 MHz, $CDCl_3$) δ 202.9, 174.3, 51.6, 43.9, 34.1, 29.1, 29.0, 29.0, 24.9, 22.1. Spectral data was in accordance with the literature.[13]

**methyl (Z)-11-(2-pentylcycloprop-1-en-1-yl)undec-9-enoate (18)**

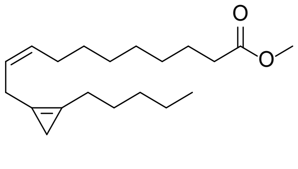


A solution of NaHMDS (1 M, 0.27 mL, 0.27 mmol, 1 eq) was added dropwise to a solution of compound **7** (128 mg, 0.12 mmol, 1 eq) in anh. THF (0.25 M) at -78 °C under $N_2$ atmopshere and stirred for 1 h. A

solution of compound **8** (60 mg, 0.32 mmol, 1.2 eq) in anh. THF (0.5 M) was added dropwise to the orange brown suspension and stirred for 1.5 h. The reaction mixture was quenched with sat. aq. $NH_4Cl$ and extracted with $Et_2O$ (3x). The combined organic layers were washed with brine, dried over $MgSO_4$ and solvents removed under reduced pressure. The crude product was purified by silica gel column chromatography ($Et_2O$ / pentane 1:99 to 1:49) to afford compound **18** (36 mg, 0.12 mmol, 44% yield) as a clear colorless oil. **$^1$H NMR** (500 MHz, $CDCl_3$) δ 5.55 – 5.41 (m, 2H), 3.66 (s, 3H), 3.12 (d, *J* = 6.3 Hz, 2H), 2.37 (tt, *J* = 7.3, 1.6 Hz, 2H), 2.29 (t, *J* = 7.6 Hz, 2H), 2.05 (q, *J* = 7.1, 6.7 Hz, 2H), 1.66 – 1.56 (m, 2H), 1.57 – 1.49 (m, 2H), 1.39 – 1.24 (m, 12H), 0.89 (t, *J* = 6.9 Hz, 3H), 0.81 (s, 2H). **$^{13}$C NMR** (126 MHz, $CDCl_3$) δ 174.4, 131.3, 125.3, 110.1, 108.1, 51.6, 34.2, 31.7, 29.6, 29.3, 29.2, 29.2, 27.3, 27.3, 26.1, 25.1, 24.8, 22.6, 14.2, 7.8.

**(Z)-11-(2-pentylcycloprop-1-en-1-yl)undec-9-enoic acid (5)**

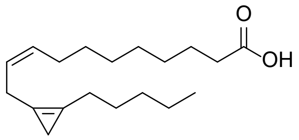


General procedure H was used with compound **18** (36 mg, 0.12 mmol, 1.0 eq) as starting material to afford the title compound **5** (25 mg, 85 µmol, 73% yield) as a light yellow oil. **$^1$H NMR** (400 MHz, $CDCl_3$) δ 11.02 (br s, 1H), 5.66 – 5.31 (m, 2H), 3.13 (d, *J* = 6.4 Hz, 2H), 2.43 – 2.29 (m, 4H), 2.12 – 2.00 (m, 2H), 1.70 – 1.49 (m, 4H), 1.43 – 1.23 (m, 12H), 0.92 – 0.87 (m, 3H), 0.81 (s, 2H). **$^{13}$C NMR** (101 MHz, $CDCl_3$) δ 180.4, 131.3, 125.3, 110.1, 108.1, 34.2, 31.8, 29.6, 29.3, 29.2, 29.2, 27.3, 27.3, 26.1, 24.8, 24.8, 22.6, 14.2, 7.8.

**methyl 7-hydroxyhept-5-ynoate (19)**

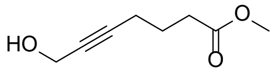


In a slightly modified procedure from the literature[14], a solution of EtMgBr (3.0 M in $Et_2O$, 45.5 mL, 136.5 mmol, 3 eq) was added to a solution of hex-5-ynoic acid (5.10 g, 45.5 mmol, 1 eq) in anh. THF (0.2 M) at 0 °C under $N_2$ atmosphere and stirred for 1 h slowly warming to rt. Then, paraformaldehyde (2.73 g, 91.0 mmol, 2 eq) was added portionwise to the grey suspension and the reaction mixture was stirred at 75 °C for 18 h. The reaction mixture was quenched with aq. 1 M HCl and extracted with DCM (3x). The combined organic layers were washed with brine, dried over $MgSO_4$ and solvents removed under reduced pressure. Thionyl chloride (6.60 mL, 91.0 mmol, 2 eq) was added carefully to a solution of the crude product in anh. MeOH (0.2 M) at 0 °C under $N_2$ atmosphere and stirred for 1 h at rt. The reaction mixture was concentrated under reduced pressure and the crude product was purified by silica gel column chromatography ($Et_2O$ / pentane 1:2) to afford compound **19** (3.60 g, 23.1 mmol, 51% yield) as a clear oil. **$^1$H NMR** (400 MHz, $CDCl_3$) δ 4.23 (t, *J* = 2.2 Hz, 2H), 3.67 (s, 3H), 2.43 (t, *J* = 7.4 Hz, 2H), 2.28 (tt, *J* = 6.9, 2.2 Hz, 2H), 1.82 (p, *J* = 7.1 Hz, 2H). **$^{13}$C NMR** (101 MHz, $CDCl_3$) δ 173.8, 85.1, 79.4, 51.8, 51.4, 32.9, 23.8, 18.3. Spectral data was in accordance with the literature.[15]

**methyl 7-bromohept-5-ynoate (20)**

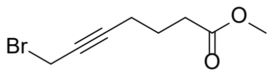

N-bromosuccinimide (1.16 g, 6.53 mmol, 1.2 eq) and triphenylphosphine (1.71 g, 6.53 mmol, 1.2 eq) were added to a solution of compound **19** (855 mg, 5.44 mmol, 1 eq) in anh. DCM (0.2 M) at 0 °C under $N_2$ atmosphere and stirred for 1 h at rt. The reaction mixture was quenched with sat. aq. $NaHCO_3$ and extracted with DCM (3x). The combined organic layers were washed with brine, dried over $MgSO_4$ and solvents removed under reduced pressure. The crude product was purified by silica gel column chromatography ($Et_2O$ / pentane 1:19) to afford compound **20** (980 mg, 4.47 mmol, 82% yield) as a light yellow oil. **$^1$H NMR** (400 MHz, $CDCl_3$) δ 3.90 (t, *J* = 2.4 Hz, 2H), 3.67 (s, 3H), 2.42 (t, *J* = 7.4 Hz, 2H), 2.31 (tt, *J* = 6.9, 2.4 Hz, 2H), 1.82 (p, *J* = 7.1 Hz, 2H). **$^{13}$C NMR** (101 MHz, $CDCl_3$) δ 173.6, 86.8, 76.3, 51.7, 32.8, 23.6, 18.5, 15.5. Spectral data was in accordance with the literature.[15]

**methyl 11-hydroxyundeca-5,8-diynoate (10)**

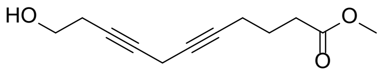


Copper(I)iodide (447 mg, 2.92 mmol, 2 eq), Sodium iodide (438 mg, 2.92 mmol, 2 eq) and $K_2CO_3$ (404 mg, 2.92 mmol, 2 eq) were added to a solution of but-3-yn-1-ol (0.22 mL, 2.92 mmol, 2 eq) in anh. DMF (0.2 M) at rt under $N_2$ atmosphere and stirred for 30 min at rt. Then, a solution of compound **20** (320 mg, 1.46 mmol, 1 eq) in anh. DMF (1.0 M) was added dropwise to the reaction mixture and stirred for 18 h. The suspension was diluted with sat. aq. $NH_4Cl$ and extracted with $Et_2O$ (3x). The combined organic layers were washed with sat. aq. $Na_2S_2O_3$ and brine, dried over $MgSO_4$ and solvents removed under reduced pressure. The crude product was purified by silica gel column chromatography ($Et_2O$ / pentane 1:1) to afford compound **10** (220 mg, 1.06 mmol, 72% yield) as a light yellow oil. **$^1$H NMR** (400 MHz, $CDCl_3$) δ 3.67 (t, *J* = 6.3 Hz, 2H), 3.65 (s, 3H), 3.10 (p, *J* = 2.4 Hz, 2H), 2.46 – 2.36 (m, 4H), 2.21 (tt, *J* = 6.9, 2.4 Hz, 2H), 2.17 – 2.04 (m, 1H), 1.79 (p, *J* = 7.1 Hz, 2H). **$^{13}$C NMR** (101 MHz, $CDCl_3$) δ 173.8, 79.5, 77.1, 76.6, 75.2, 61.2, 51.7, 33.0, 23.9, 23.2, 18.3, 9.8. Spectral data was in accordance with the literature.[15]

**methyl (5Z,8Z)-11-hydroxyundeca-5,8-dienoate (21)**

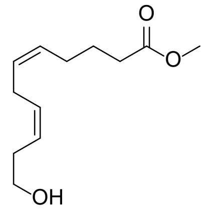


In a slightly modified procedure from the literature[16], a solution of $NaBH_4$ (111 mg, 2.94 mmol, 2 eq) in degassed anh. MeOH (1.5 M) was added to a solution of Nickel(II) acetate hydrate (658 mg, 2.64 mmol, 1.8 eq) in MeOH (1.5 M) at rt under $N_2$ atmosphere, the solution turned dark black immediately and was purged with $H_2$ for 5 min. Ethylenediamine (0.18 ml, 2.64 mmol, 1.8 eq) and a solution of compound **10** (306 mg, 1.47 mmol, 1 eq) in MeOH (1.5 M) were added and the reaction mixture was stirred under positive $H_2$ pressure for 2 h at rt and monitored using LCMS. The suspension was diluted with $Et_2O$, filtered over celite, washed with brine and dried over $MgSO_4$. The crude product was purified by silica gel column chromatography (Et2O / pentane 1:1) to afford a mixture of under / over reduced or even cis / trans products (200 mg, 0.94 mmol, 64 % yield). This was purified by HPLC (Solvent system: A: water (gradient, 10-90%), B: $CH_3CN$ (gradient, 90-10%), C: (constant, 10%) 1% TFA (aq.) to afford compound **21** (84 mg, 0.40 mmol, 27% yield) as a clear colorless oil. **$^1$H NMR** (400 MHz, $CDCl_3$) δ 5.52 – 5.25 (m, 4H), 3.62 (s, 3H), 3.59 (t, *J* = 6.6 Hz, 2H), 2.81 – 2.72 (m, 2H), 2.36 – 2.24 (m, 4H), 2.13 (s, 1H), 2.10 – 2.01 (m, 2H), 1.65 (p, *J* = 7.5 Hz, 2H). **$^{13}$C NMR** (101 MHz, $CDCl_3$) δ 174.3, 130.8,

129.0, 128.8, 125.7, 62.2, 51.6, 33.4, 30.9, 26.6, 25.8, 24.7. Spectral data was in accordance with the literature.[17]

**methyl (5Z,8Z)-11-oxoundeca-5,8-dienoate (9)**

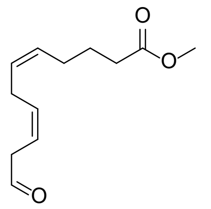

Dess-Martin periodinane (201 mg, 0.47 mmol, 1.2 eq) was added to a solution of compound **21** (84 mg, mmol, 1 eq) in anh. DCM (0.2 M) at 0 °C under $N_2$ atmosphere and stirred for 1 h at rt. The reaction mixture was quenched with sat. aq. $NaHCO_3$ and extracted with $Et_2O$ (3x). The combined organic layers were washed with brine, dried over $MgSO_4$ and solvents removed under reduced pressure. The crude product was purified by silica gel column chromatography ($Et_2O$ / pentane 1:2) to afford compound **22** (31 mg, 0.15 mmol, 37% yield) as a clear oil. **$^1$H NMR** (400 MHz, $CDCl_3$) δ 9.66 (t, *J* = 1.9 Hz, 1H), 5.72 – 5.49 (m, 2H), 5.46 – 5.30 (m, 2H), 3.65 (s, 3H), 3.26 – 3.16 (m, 2H), 2.80 – 2.73 (m, 2H), 2.30 (t, *J* = 7.4 Hz, 2H), 2.13 – 2.03 (m, 2H), 1.74 – 1.62 (m, 2H). **$^{13}$C NMR** (101 MHz, $CDCl_3$) δ 199.5, 174.1, 133.3, 129.7, 127.9, 118.7, 51.6, 42.6, 33.5, 26.7, 26.0, 24.8. Spectral data was in accordance with the literature.[18]

**methyl (5Z,8Z,11Z)-13-(2-pentylcycloprop-1-en-1-yl)trideca-5,8,11-trienoate (22)**

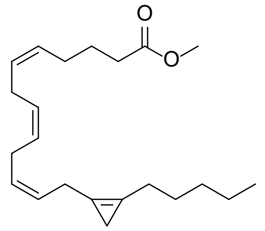

A solution of NaHMDS (1 M, 0.29 mL, 0.29 mmol, 2 eq) was added dropwise to a solution of compound **7** (140 mg, 0.29 mmol, 2 eq) in anh. THF (0.25 M) at -78 °C under $N_2$ atmopshere and stirred for 1 h. A solution of compound **9** (31 mg, 0.15 mmol, 1 eq) in anh. THF (0.5 M) was added dropwise to the orange brown suspension and stirred for 1.5 h. The reaction mixture was quenched with sat. aq. $NH_4Cl$ and extracted with $Et_2O$ (3x). The combined organic layers were washed with brine, dried over $MgSO_4$ and solvents removed under reduced pressure. The crude product was purified by silica gel column chromatography ($Et_2O$ / pentane 1:99 to 1:49) to afford compound **22** (26 mg, 78 µmol, 54% yield) as a clear colorless oil. **$^1$H NMR** (500 MHz, $CDCl_3$) δ 5.60 – 5.40 (m, 2H), 5.42 – 5.29 (m, 4H), 3.66 (s, 3H), 3.17 (d, *J* = 7.0 Hz, 2H), 2.85 (t, *J* = 6.5 Hz, 2H), 2.80 (t, *J* = 5.8 Hz, 2H), 2.38 (t, *J* = 7.3 Hz, 2H), 2.32 (t, *J* = 7.5 Hz, 2H), 2.11 (q, *J* = 7.0 Hz, 2H), 1.70 (p, *J* = 7.5 Hz, 2H), 1.54 (p, *J* = 7.0 Hz, 2H), 1.37 – 1.26 (m, 4H), 0.89 (t, *J* = 6.7 Hz, 3H), 0.82 (s, 2H). **$^{13}$C NMR** (126 MHz, $CDCl_3$) δ 174.2, 129.2, 129.1, 129.0, 128.4, 128.1, 125.8, 110.4, 107.8, 51.6, 33.6, 31.7, 27.2, 26.7, 26.0, 25.8, 25.7, 24.9, 24.8, 22.6, 14.2, 7.8.

**(5Z,8Z,11Z)-13-(2-pentylcycloprop-1-en-1-yl)trideca-5,8,11-trienoic acid (6)**

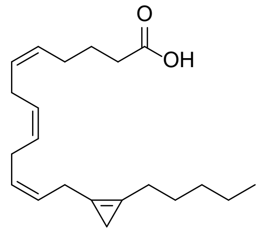

General procedure H was used with compound **22** (26 mg, 78 µmol, 1.0 eq) as starting material to afford the title compound **6** (25 mg, 78 µmol, quantitative yield) as a clear colorless oil. **$^1$H NMR** (500 MHz, $CDCl_3$) δ 11.24 (br s, 1H), 5.60 – 5.41 (m, 2H), 5.44 – 5.30 (m, 4H), 3.17 (d, *J* = 7.0 Hz, 2H), 2.85 (t, *J* = 6.6 Hz, 2H), 2.80 (t, *J* = 6.1 Hz, 2H), 2.38 (q, *J* = 8.2 Hz, 4H), 2.13 (q, *J* = 7.1 Hz, 2H), 1.72 (p, *J* = 7.5 Hz, 2H), 1.55 (p, *J* = 7.2 Hz, 2H), 1.37 – 1.25 (m, 4H), 0.89 (t, *J* = 6.5 Hz, 3H), 0.83 (s, 2H). **$^{13}$C NMR** (126 MHz, $CDCl_3$) δ 180.0, 129.2, 128.9, 128.4, 128.2, 125.8, 110.4, 107.9, 33.5, 31.8, 27.3, 26.6, 26.1, 25.8, 25.8, 24.8, 24.6, 22.6, 14.2, 7.9.

**8-(2-hexylcycloprop-1-en-1-yl)octanoic acid (3)**

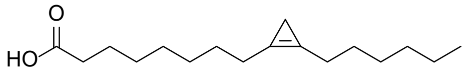

In a slightly modified procedure from the literature[7], thionylchloride (0.48 mL, 6.62 mmol, 8.1 eq) was added to a solution of compound **s14** (254 mg, 0.819 mmol, 1.0 eq) in anhydrous $Et_2O$ (0.2 M) under $N_2$ atmosphere at 0 °C and stirred for 1 h. The reaction mixture was concentrated under reduced pressure and a solution of the crude acid chloride in anhydrous DCM (0.2 M) was added to anhydrous $ZnCl_2$ (221 mg, 1.622 mmol, 2.0 eq) under $N_2$ atmosphere. The suspension was stirred for 1 h and periodically flushed with $N_2$ gas, after which additional anhydrous $ZnCl_2$ (271 mg, 1.988 mmol, 2.4 eq) was added and stirred for another 2 hours. Then, methanol (0.035 mL, 0.87 mmol, 1.06 eq) was added dropwise and stirred for 30 min. The reaction mixture was added to a solution of $NaBH_4$ (167 mg, 4.41 mmol, 5.4 eq) and NaOH (74 mg, 1.85 mmol, 2.25 eq) in anhydrous meOH (0.1 M) at -30 °C and stirred for 1 h gradually warming to rt. The reaction was quenched with water, acidified with aq. 1 M HCl and extracted with DCM (3x). The combined organic layers were washed with water, sat. aq. $NaHCO_3$ and brine, dried over $MgSO_4$ and concentrated under reduced pressure. The crude product was purified by silica gel column chromatography ($Et_2O$ / pentane 1:9) to afford compound **3** (21 mg, 0.079 mmol, 10% yield). **$^1$H NMR** (400 MHz, $CDCl_3$) δ 2.37 (t, *J* = 7.2Hz, 4H), 2.35 (t, *J* = 7.68Hz, 2H), 1.71 – 1.58 (m, 2H), 1.59 – 1.46 (m, 4H), 1.42 – 1.15 (m, 12H), 0.88 (t, *J* = 6.8 Hz, 3H), 0.76 (s, 2H). **$^{13}$C NMR** (101 MHz, $CDCl_3$) δ 179.7, 109.5, 109.2, 34.0, 31.7, 29.2, 29.1, 29.0, 29.0, 27.4, 27.3, 26.1, 26.0, 24.7, 22.6, 14.1, 7.4.

**1,1,2-tribromo-2-methylcyclopropane (25)**

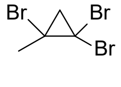

General procedure C was used with 2-bromoprop-1-ene **23** (4.50 g, 37.20 mmol, 1.0 eq) as starting material to afford the title compound **25** (5.10 g, 17.42 mmol, 47% yield) as a colorless liquid. **$^1$H NMR** (300 MHz, $CDCl_3$) δ 2.08 (s, 3H), 1.98 (d, *J* = 9.2 Hz, 1H), 1.84 (d, *J* = 9.2 Hz, 1H). **$^{13}$C NMR** (75 MHz, $CDCl_3$) δ 39.7, 38.6, 33.3, 30.0. Spectral data was in accordance with the literature.[8]

**1-(12-iodododecyl)-2-methylcycloprop-1-ene (27)**

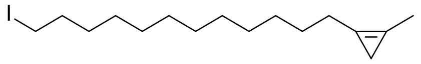


General procedure D was used with compound **25** (410 mg, 1.40 mmol, 1.0 eq) and 1,12-diiodododecane (1.48 g, 3.50 mmol, 2.5 eq) as starting materials to afford the title compound **27** (134 mg, 0.38 mmol, 27 % yield). **$^{1}$H NMR** (300 MHz, $CDCl_3$) δ 3.18 (t, *J* = 7.1 Hz, 2H), 2.36 (tq, *J* = 7.3, 1.7 Hz, 2H), 2.03 (t, *J* = 1.6 Hz, 3H), 1.82 (p, *J* = 7.1 Hz, 2H), 1.58 – 1.48 (m, 2H), 1.43 – 1.24 (m, 16H), 0.77 (s, 2H). **$^{13}$C NMR** (75 MHz, $CDCl_3$) δ 110.2, 105.3, 33.7, 30.7, 29.7, 29.7, 29.6, 29.5, 28.7, 27.5, 26.1, 11.7, 8.3, 7.5.

**13-(2-methylcycloprop-1-en-1-yl)tridecanenitrile (30)**

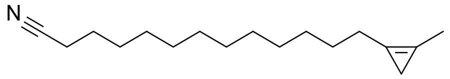


General procedure E was used with compound **27** (134 mg, 0.38 mmol, 1.0 eq) as starting material to afford the title compound **30** (76 mg, 0.31 mmol, 80 % yield). **$^{1}$H NMR** (300 MHz, $CDCl_3$) δ 2.40 – 2.32 (m, 2H), 2.33 (t, *J* = 7.1 Hz, 2H), 2.03 (t, *J* = 1.6 Hz, 3H), 1.73 – 1.57 (m, 2H), 1.59 – 1.38 (m, 4H), 1.37 – 1.18 (m, 14H), 0.77 (s, 2H). **$^{13}$C NMR** (75 MHz, $CDCl_3$) δ 120.0, 110.2, 105.3, 29.7, 29.6, 29.5, 29.5, 29.4, 28.9, 28.8, 27.5, 26.1, 25.5, 17.3, 11.7, 8.2.

**13-(2-methylcycloprop-1-en-1-yl)tridecanoic acid (1)**

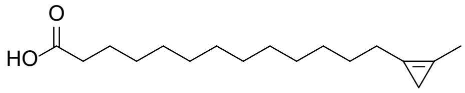


General procedure F was used with compound **30** (76 mg, 0.31 mmol, 1.0 eq) as starting material to afford the title compound **1** (30 mg, 0.11 mmol, 37% yield). **$^{1}$H NMR** (300 MHz, $CDCl_3$) δ 2.41 – 2.29 (m, 4H), 2.03 (t, *J* = 1.5 Hz 3H), 1.63 (p, *J* = 7.4 Hz, 2H), 1.59 – 1.46 (m, 2H), 1.38 – 1.16 (m, 16H), 0.77 (s, 2H). $^{13}$C NMR (75 MHz, $CDCl_3$) δ 179.8, 110.1, 105.2, 34.0, 29.6, 29.6, 29.6, 29.4 29.4, 29.4, 29.3, 29.1, 27.4, 26.0, 24.7, 11.5, 8.1.

**1-(14-iodotetradecyl)-2-methylcycloprop-1-ene (28)**

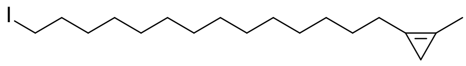


General procedure D was used with compound **25** (488 mg, 1.67 mmol, 1.0 eq) and 1,14-diiodotetradecane (1.58 g, 3.34 mmol, 2.2 eq) as starting materials to afford the title compound **28** (280 mg, 0.74 mmol, 45 % yield). **$^{1}$H NMR** (300 MHz, $CDCl_3$) δ 3.18 (t, *J* = 7.1 Hz, 2H), 2.48 – 2.28 (m, 2H), 2.03 (t, *J* = 1.6 Hz, 3H), 1.82 (p, *J* = 7.1 Hz, 2H), 1.60 – 1.47 (m, 2H), 1.43 – 1.17 (m, 20H), 0.77 (s, 2H). **$^{13}$C NMR** (75 MHz, $CDCl_3$) δ 110.2, 105.3, 33.7, 30.7, 29.8, 29.8, 29.7, 29.6, 29.5, 28.7, 27.5, 26.1, 11.7, 8.3, 7.3.

**15-(2-methylcycloprop-1-en-1-yl)pentadecanenitrile (31)**

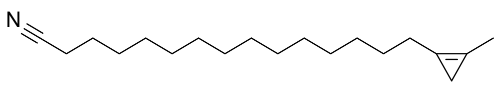

General procedure E was used with compound **28** (280 mg, 0.74 mmol, 1.0 eq) as starting material to afford the title compound **31** (140 mg, 0.51 mmol, 68 % yield). **$^1$H NMR** (300 MHz, $CDCl_3$) δ 2.49 – 2.28 (m, 4H), 2.03 (t, *J* = 1.6 Hz 3H), 1.75 – 1.40 (m, 6H), 1.39 – 1.18 (m, 18H), 0.79 (s, 2H). **$^{13}$C NMR** (75 MHz, $CDCl_3$) δ 120.0, 110.2, 105.3, 29.8, 29.7, 29.6, 29.6, 29.5, 29.4, 28.9, 28.8, 27.5, 26.1, 25.5, 17.3, 11.7, 8.2.

**15-(2-methylcycloprop-1-en-1-yl)pentadecanoic acid (2)**

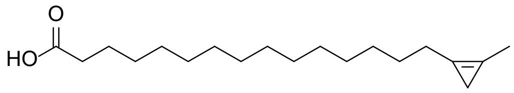


General procedure F was used with compound **31** (140 mg, 0.51 mmol, 1.0 eq) as starting material to afford the title compound **2** (89 mg, 0.30 mmol, 60% yield). **$^1$H NMR** (300 MHz, $CDCl_3$) δ 10.38 (br s, 1H), 2.43 – 2.29 (m, 4H), 2.03 (t, *J* = 1.6 Hz, 3H), 1.63 (p, *J* = 7.4 Hz, 2H), 1.59 – 1.46 (m, 2H), 1.38 – 1.17 (m, 20H), 0.77 (s, 2H). **$^{13}$C NMR** (75 MHz, $CDCl_3$) δ 180.6, 110.2, 105.3, 34.3, 29.8, 29.8, 29.8, 29.8, 29.7, 29.6, 29.6, 29.6, 29.4, 29.2, 27.5, 26.1, 24.8, 11.7, 8.3.

**2-bromodec-1-ene (24)**

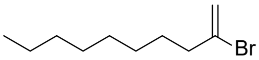


General procedure B was used with dec-1-yne (0.90 mL, 5.0 mmol, 1.0 eq) as starting material to afford the title compound **24** (854 mg, 3.90 mmol, 78% yield) as a colorless liquid. **$^1$H NMR** (400 MHz, $CDCl_3$): δ 5.55 (dt, *J* = 1.6 Hz, 1.2 Hz, 1H), 5.38 (d, *J* = 1.6 Hz, 1H), 2.49 – 2.34 (dt, *J* = 2.8, 1.2, 3H), 1.65 – 1.49 (m, 3H), 0.95 – 0.82 (t, *J* = 7.0 Hz, 3H). **$^{13}$C NMR** (101 MHz, $CDCl_3$): δ 135.1, 116.3, 41.6, 32.0, 29.4, 29.4, 28.6, 28.1, 22.8, 14.3. Spectral data was in accordance with the literature.[8]

**1,1,2-tribromo-2-octylcyclopropane (26)**

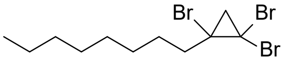


General procedure C was used with compound **24** (3.22 g, 14.7 mmol, 1.0 eq) as starting material to afford the title compound **26** (2.69 g, 6.88 mmol, 47% yield) as a colorless liquid. **$^1$H NMR** (400 MHz, $CDCl_3$): δ 2.11-1.93 (m, 2H) 1.97 – 1.93 (d, *J* = 9.2 Hz 1H), 1.82 (d, *J* = 9.2 Hz, 1H), 1.79 – 1.53 (m, 1H), 1.43 – 1.16 (m, 14H), 0.95 – 0.85 (t, *J* = 7.0 Hz , 3H). **$^{13}$C NMR** (101 MHz, $CDCl_3$): δ 46.0, 41.9, 38.2, 33.3, 32.0, 29.6, 29.4, 29.1, 27.8, 22.8, 14.3. Spectral data was in accordance with the literature.[3]

**1-(7-iodoheptyl)-2-octylcycloprop-1-ene (29)**

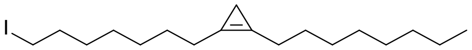


General procedure D was used with compound **26** (3.91 g, 10.0 mmol, 1.0 eq) and 1,7-diiodoheptane (8.10 g, 23.0 mmol, 2.3 eq) as starting materials to afford the title compound **29** (2.10 g, 5.59 mmol, 56 % yield). **$^1$H NMR** (400 MHz, $CDCl_3$): δ 3.18 (t, *J* = 7.0 Hz, 2H), 2.41 – 2.33 (m, 4H), 1.82 (p, *J* = 7.1 Hz, 2H), 0.92-0.85 (m, 3H), 0.77 (s, 2H). ). **$^{13}$C NMR** (101 MHz, $CDCl_3$): δ 109.6, 109.3, 33.7, 32.0, 30.6, 29.6, 29.4, 29.3, 28.5, 27.5, 27.4, 26.2, 26.1, 22.8, 14.3, 7.5, 7.3.

**8-(2-octylcycloprop-1-en-1-yl)octanenitrile (32)**

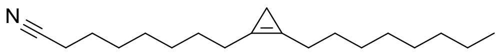

General procedure E was used with compound **29** (2.10 g, 5.59 mmol, 1.0 eq) as starting material to afford the title compound **32** (1.39 g, 5.00 mmol, 89 % yield) as a clear oil. $^1$**H NMR** (300 MHz, $CDCl_3$): δ 0.92 – 0.85 (m, 3H), 0.76 (s, 2H). $^{13}$**C NMR** (75 MHz, $CDCl_3$): δ 119.9, 109.7, 109.2, 32.0, 29.5, 29.4, 29.2, 28.8, 28.7, 27.5, 27.4, 26.2, 26.0, 25.5, 22.8, 17.3, 14.3, 7.5.

**8-(2-octylcycloprop-1-en-1-yl)octanoic acid (4)**

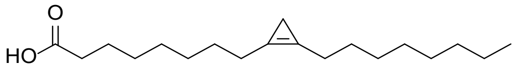

General procedure F was used with compound **32** (1.35 g, 4.92 mmol, 1.0 eq) as starting material to afford the title compound **4** (909 mg, 3.09 mmol, 63% yield) as a light yellow oil. $^1$**H NMR** (300 MHz, $CDCl_3$): δ 2.24-2.28 (m, 6H) 0.93 – 0.83 (m, 3H), 0.77 (s, 2H). $^{13}$**C NMR** (75 MHz, $CDCl_3$): δ 180.3, 109.6, 109.4, 34.2, 32.0, 29.6, 29.4, 29.3, 29.2, 27.5, 27.5, 26.2, 26.1, 24.8, 22.8, 14.3, 7.5.

**9-bromononanoic acid (s4)**

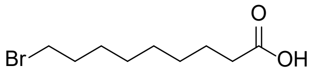

General procedure A was used with compound 9-bromononan-1-ol (3.05 g, 13.67 mmol, 1.0 eq) as starting material, the crude product was purified by silica gel column chromatography ($Et_2O$ / pentane 1:19, 1% AcOH) to afford compound **s4** (3.09 g, 13.0 mmol, 95% yield) as a yellow oil. $^1$**H NMR** (500 MHz, $CDCl_3$) δ 11.64 (s, 1H), 3.39 (t, *J* = 6.8 Hz, 2H), 2.34 (t, *J* = 7.5 Hz, 2H), 1.89 – 1.79 (m, 2H), 1.67 – 1.58 (m, 2H), 1.46 – 1.38 (m, 2H), 1.37 – 1.27 (m, 6H).$^{13}$**C NMR** (126 MHz, $CDCl_3$) δ 180.6, 34.2, 34.1, 32.9, 29.1, 29.0, 28.7, 28.2, 24.7**.** Spectral data was in accordance with the literature.[19]

**(8-carboxyoctyl)triphenylphosphonium bromide (s5)**

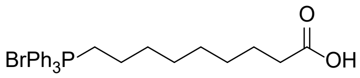

General procedure G was used with compound **s4** (3.09 g, 13.0 mmol, 1.0 eq) as starting material, the crude product was purified by silica gel column chromatography (DCM / MeOH 99:1 to 1:19, 1% AcOH) to afford compound **s5** (6.45 g, 12.9 mmol, quant. yield) as a yellow oil. $^1$**H NMR** (500 MHz, $CDCl_3$) δ 7.80 – 7.72 (m, 9H), 7.70 – 7.65 (m, 6H), 3.62 – 3.50 (m, 2H), 2.28 (t, *J* = 7.4 Hz, 2H), 1.56 (dd, *J* = 10.4, 6.2 Hz, 4H), 1.48 (q, *J* = 7.3 Hz, 2H), 1.26 – 1.11 (m, 6H). $^{13}$**C NMR** (126 MHz, $CDCl_3$) δ 177.2, 135.2, 135.2, 133.7, 133.6, 130.7, 130.6, 118.6, 117. 9, 34.5, 30.2, 30.0, 28.5, 28.5, 24.6, 22.9, 22.5, 22.5. Spectral data was in accordance with the literature.[20]

**(2-(1,3-dioxan-2-yl)ethyl)triphenylphosphonium bromide (s6)**

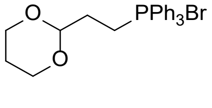

General procedure A was used with 2-(2-bromoethyl)-1,3-dioxane (1.0 mL, 7.34 mmol, 1.0 eq) as starting material to afford compound **s6** (500 mg, 1.09 mmol, 15% yield) as a yellow oil. **$^1$H NMR** (300 MHz, $CDCl_3$) δ 7.74 – 7.39 (m, 15H), 4.79 (t, *J* = 4.7 Hz, 1H), 3.83 – 3.73 (m, 2H), 3.60 (td, *J* = 12.2, 2.4 Hz, 2H), 3.54 – 3.38 (m, 2H), 1.86 – 1.74 (m, 1H), 1.73 – 1.59 (m, 2H), 1.16 – 1.05 (m, 1H). **$^{13}$C NMR** (75 MHz, $CDCl_3$) δ 134.8, 134.8, 133.1, 133.0, 130.3, 130.1, 118.0, 116.8, 99.0, 98.8, 66.3, 27.5, 27.5, 25.1, 17.6, 16.9.

**methyl 5-oxopentanoate (s7)**

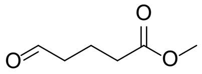

Triethylamine (22.6 mL, 122 mmol, 5 eq) was added too a solution of δ-valerolactone (3.0 mL, 32.4 mmol, 1 eq) in MeOH (0.5 M) at rt and the solution was stirred for 1 h. The solvents were removed and coevaporated with toluene to afford the crude alcohol. Dess-Martin periodinane (16.3 g, 38.4 mmol, 1.2 eq) was added to a solution of the crude alcohol (5.13 g, 38.9 mmol, 1 eq) in anh. DCM at 0 °C under $N_2$ atmosphere and stirred at rt for 2 h. The reaction mixture was quenched with sat. aq. $NaHCO_3$ and extracted with $Et_2O$ (3x). The combined organic layers were washed with brine, dried over $MgSO_4$ and solvents removed under reduced pressure. The crude product was purified by silica gel column chromatography ($Et_2O$ / pentane 1:4) to afford compound **s7** (1.96 g, 15.0 mmol, 47% yield) as a light yellow oil. **$^1$H NMR** (400 MHz, $CDCl_3$) δ 9.75 (t, *J* = 1.2 Hz, 1H), 3.65 (d, *J* = 0.9 Hz, 3H), 2.51 (tt, *J* = 7.2, 1.1 Hz, 2H), 2.36 (t, *J* = 7.3 Hz, 2H), 2.06 – 1.84 (m, 2H). **$^{13}$C NMR** (101 MHz, $CDCl_3$) δ 201.6, 173.5, 51.7, 43.0, 33.0, 17.4. Spectral data was in accordance with the literature.[21]

**methyl (Z)-7-(1,3-dioxan-2-yl)hept-5-enoate (s8)**

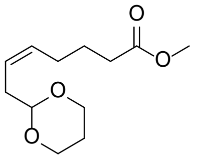

A solution of NaHMDS (1 M, 11.5 mL, 11.5 mmol, 1.3 eq) was added dropwise to a solution of compound **s6** (5.25 g, 11.5 mmol, 1.3 eq) in anh. THF (0.25 M) at -78 °C under $N_2$ atmopshere, slowly warmed to rt and stirred for 1 h. The reaction was cooled back to -78 °C and a solution of compound **s7** (1.15 g, 8.83 mmol, 1 eq) in anh. THF (0.5 M) was added dropwise to the orange suspension and stirred for 1.5 h. The reaction mixture was quenched with sat. aq. $NH_4Cl$ and extracted with $Et_2O$ (3x). The combined organic layers were washed with brine, dried over $MgSO_4$ and solvents removed under reduced pressure. The crude product was purified by silica gel column chromatography (acetone / pentane 1:49 to 1:19) to afford compound **s8** (1.31 g, 5.75 mmol, 65% yield) as a clear colorless oil. **$^1$H NMR** (300 MHz, $CDCl_3$) δ 5.58 – 5.33 (m, 2H), 4.51 (t, *J* = 5.2 Hz, 1H), 4.18 – 3.99 (m, 2H), 3.80 – 3.69 (m, 2H), 3.64 (s, 3H), 2.39 – 2.21 (m, 4H), 2.16 – 1.94 (m, 3H), 1.69 (dt, *J* = 8.3, 7.1 Hz, 2H), 1.32 (ddt, *J* = 13.5, 2.6, 1.3 Hz, 1H). **$^{13}$C NMR** (75 MHz, $CDCl_3$) δ 174.1, 131.3, 124.3, 101.9, 67.0, 51.5, 33.5, 26.8, 25.8, 24.8. Spectral data was in accordance with the literature.[22]

**hexadec-9-yn-1-ol (s10)**

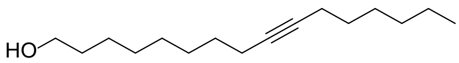

In a slightly modified procedure from the literature[5], *n*-BuLi (2.5 M in hexanes, 8.80 mL, 22 mmol, 2.2 eq) was added dropwise to a solution of dec-9-yn-1-ol (1.54 g, 10.0 mmol, 1 eq) in 4:1 THF / HMPA

(0.1 M) at -78 °C under $N_2$ atmosphere and the reaction mixture was stirred for 1 h gradually warming to rt. The formed suspension was cooled back to -78 °C, 1-bromohexane (1.35 ml, 9.6 mmol, 0.96 eq) and NaI (75 mg, 0.50 mmol, 0.05 eq) were added dropwise and the reaction was stirred for 18 h slowly warming to rt. The solution was quenched with water and extracted with 1:1 diethyl ether / pentane (3x). The combined organic layers were washed with brine, dried over $MgSO_4$ and concentrated under reduced pressure. The crude product was purified by silica gel column chromatography ($Et_2O$ / pentane 1:19 to 1:9) to afford compound **s10** (1.04 g, 4.38 mmol, 43.8 % yield) as a colorless oil. **$^1$H NMR** (400 MHz, $CDCl_3$) δ 3.47 (t, *J* = 6.8 Hz, 2H), 3.22 (s, 1H), 2.02 (t, *J* = 7.0 Hz, 4H), 1.56 – 1.09 (m, 20H), 0.78 (t, *J* = 7.0 Hz, 3H). $^{13}$C NMR (101 MHz, $CDCl_3$) δ 80.1, 80.0, 62.4, 32.6, 31.3, 29.4, 29.1, 29.1, 29.1, 28.8, 28.5, 25.7, 22.5, 18.7, 18.7, 14.0.

**hexadec-9-ynoic acid (s11)**

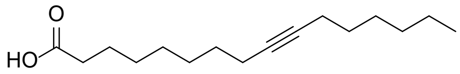


General procedure A was used with compound **s10** (402 mg, 1.69 mmol, 1.0 eq) as starting material, the crude product was purified by silica gel column chromatography ($Et_2O$ / pentane 1:9) to afford compound **s11** (357 mg, 1.41 mmol, 84% yield) as a light yellow crystalline solid. **$^1$H NMR** (400 MHz, $CDCl_3$) δ 2.33 (t, *J* = 7.5 Hz, 2H), 2.12 (t, *J* = 6.6 Hz, 4H), 1.69 – 1.57 (m, 2H), 1.51 – 1.17 (m, 16H), 0.87 (t, *J* = 6.9 Hz, 3H). **$^{13}$C NMR** (101 MHz, $CDCl_3$) δ 180.6, 80.4, 80.1, 34.2, 31.5, 29.2, 29.2, 29.1, 28.9, 28.7, 28.7, 24.7, 22.7, 18.9, 18.8, 14.2. Spectral data was in accordance with the literature.[6]

**methyl hexadec-9-ynoate (s12)**

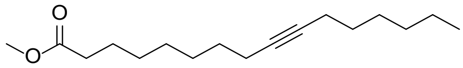


Thionylchloride (0.41 mL, 5.65 mmol, 4.0 eq) was added to a solution of compound **s11** (357 mg, 1.41 mmol, 1.0 eq) in methanol (0.2 M) at 0 °C under nitrogen atmosphere and stirred for 2.5 h at rt. The reaction mixture was concentrated under reduced pressure and the crude product was purified by silica gel column chromatography ($Et_2O$ / pentane 1:49) to afford compound **s12** (333 mg, 1.25 mmol, 88% yield) as a clear oil. **$^1$H NMR** (300 MHz, $CDCl_3$) δ 3.62 (s, 3H), 2.26 (t, *J* = 7.5 Hz, 2H), 2.13 – 2.04 (m, 4H), 1.65 – 1.51 (m, 2H), 1.49 – 1.15 (m, 16H), 0.85 (t, *J* = 6.7 Hz, 3H). **$^{13}$C NMR** (75 MHz, $CDCl_3$) δ 174.3, 80.4, 80.1, 51.4, 34.1, 31.5, 29.2, 29.1, 29.1, 28.9, 28.7, 28.6, 25.0, 22.7, 18.8, 18.8, 14.1

**ethyl 2-hexyl-3-(8-methoxy-8-oxooctyl)cycloprop-2-ene-1-carboxylate (s13)**

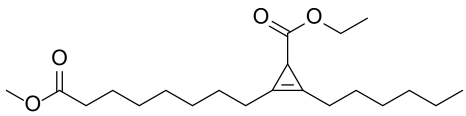


A solution of ethyl diazoacetate (15 wt. % in DCM, 0.9 mL, 1.29 mmol, 1.0 eq) was added dropwise over 3 hours to a suspension of compound **s12** (333 mg, 1.25 mmol, 1.0 eq) and rhodium(II) acetate dimer (28 mg, 0.063 mmol, 0.05 eq) in DCM (0.2 M) at 0 °C under $N_2$ and stirred for another 30 minutes. The reaction mixture was concentrated under reduced pressure and the crude product was purified by silica gel column chromatography ($Et_2O$ / pentane 1:49 to 1:9) to afford the compound **s13** (327 mg, 0.928 mmol, 76% yield) as a light yellow oil. **$^1$H NMR** (400 MHz, $CDCl_3$) δ 4.06 (q, *J* = 7.1 Hz, 2H), 3.62 (s, 3H), 2.35 (t, *J* = 7.3 Hz, 4H), 2.26 (t, *J* = 7.5 Hz, 2H), 1.98 (s, 1H), 1.62 – 1.55 (m, 2H), 1.55

– 1.45 (s, 4 Hz), 1.35 – 1.16 (m, 12H), 1.20 (t, *J* = 7.2 Hz, 3H), 0.85 (t, *J* = 6.8 Hz, 3H). **$^{13}$C NMR** (101 MHz, $CDCl_3$) δ 177.1, 174.3, 105.8, 105.6, 59.8, 51.5, 34.1, 31.6 29.1, 29.1, 29.0, 29.0, 27.0, 27.0, 25.0, 24.6, 24.5, 22.6, 22.3, 14.5, 14.1.

**2-(7-carboxyheptyl)-3-hexylcycloprop-2-ene-1-carboxylic acid (s14)**

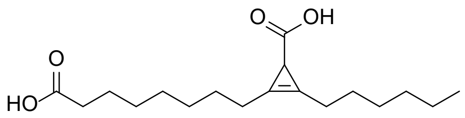


An aqueous solution of KOH (2.0 M, 4.2 mmol, 2.1 mL, 5.0 eq) was added to a solution of compound **s13** (296 mg, 0.840 mmol, 1.0 eq) in ethanol (0.2 M) and refluxed for 2.5 h. The reaction mixture was acidified with aq. 1 M HCl and extracted with $Et_2O$ (5x). The combined organic layers were washed with brine, dried over $MgSO_4$ and concentrated under reduced pressure. The crude product was purified by silica gel column chromatography ($Et_2O$ / pentane 1:3 + 1% AcOH) to afford the compound **s14** (254 mg, 0.819 mmol, 97% yield) as a clear oil. **$^1$H NMR** (400 MHz, $CDCl_3$) δ 11.07 (s, 2H), 2.38 (t, *J* = 7.2 Hz, 4H), 2.30 (t, *J* = 7.5 Hz, 2H), 1.99 (s, 1H), 1.64 – 1.46 (m, 6H), 1.38 – 1.18 (m, 12H), 0.85 (t, *J* = 6.7 Hz, 3H).

**8-(2-hexylcycloprop-1-en-1-yl)octanoic acid (3)**

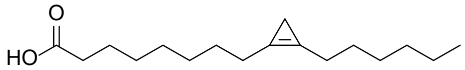


In a slightly modified procedure from the literature[23], thionylchloride (0.48 mL, 6.62 mmol, 8.1 eq) was added to a solution of compound **s14** (254 mg, 0.819 mmol, 1.0 eq) in anhydrous $Et_2O$ (0.2 M) under $N_2$ atmosphere at 0 °C and stirred for 1 h. The reaction mixture was concentrated under reduced pressure and a solution of the crude acid chloride in anhydrous DCM (0.2 M) was added to anhydrous $ZnCl_2$ (221 mg, 1.622 mmol, 2.0 eq) under $N_2$ atmosphere. The suspension was stirred for 1 h and periodically flushed with $N_2$ gas, after which additional anhydrous $ZnCl_2$ (271 mg, 1.988 mmol, 2.4 eq) was added and stirred for another 2 hours. Then, methanol (0.035 mL, 0.87 mmol, 1.06 eq) was added dropwise and stirred for 30 min. The reaction mixture was added to a solution of $NaBH_4$ (167 mg, 4.41 mmol, 5.4 eq) and NaOH (74 mg, 1.85 mmol, 2.25 eq) in anhydrous meOH (0.1 M) at -30 °C and stirred for 1 h gradually warming to rt. The reaction was quenched with water, acidified with aq. 1 M HCl and extracted with DCM (3x). The combined organic layers were washed with water, sat. aq. $NaHCO_3$ and brine, dried over $MgSO_4$ and concentrated under reduced pressure. The crude product was purified by silica gel column chromatography ($Et_2O$ / pentane 1:9) to afford compound **3** (21 mg, 0.079 mmol, 10% yield). **$^1$H NMR** (400 MHz, $CDCl_3$) δ 2.37 (t, *J* = 7.2Hz, 4H), 2.35 (t, *J* = 7.68Hz, 2H), 1.71 – 1.58 (m, 2H), 1.59 – 1.46 (m, 4H), 1.42 – 1.15 (m, 12H), 0.88 (t, *J* = 6.8 Hz, 3H), 0.76 (s, 2H). **$^{13}$C NMR** (101 MHz, $CDCl_3$) δ 179.7, 109.5, 109.2, 34.0, 31.7, 29.2, 29.1, 29.0, 29.0, 27.4, 27.3, 26.1, 26.0, 24.7, 22.6, 14.1, 7.4.

## References


[1] S. Guria, C. G. Daniliuc, U. Hennecke, *Adv. Synth. Catal.* **2021**, *363*, 3852–3858.

[2] T. Ando, R. Ohno, K. Ikemoto, Y. Yajima, *J. Pestic. Sci.* **1995**, *20*, 25–32.

[3] J. R. Al Dulayymi, M. S. Baird, M. J. Simpson, S. Nyman, G. R. Port, *Tetrahedron* **1996**, *52*, 12509–12520.

[4] B. W. Gung, A. O. Omollo, *European J. Org. Chem.* **2008**, 4790–4795.

[5] L. Lin, Q. Zhao, A. N. Li, F. Ren, F. Yang, R. Wang, *Org. Biomol. Chem.* **2009**, *7*, 3663–3665.

[6] D. Tasdemir, D. Sanabria, I. L. Lauinger, A. Tarun, R. Herman, R. Perozzo, M. Zloh, S. H. Kappe, R. Brun, N. M. Carballeira, *Bioorganic Med. Chem.* **2010**, *18*, 7475–7485.

[7] W. J. Gensler, M. B. Floyd, R. Yanase, K. W. Pober, *J. Am. Chem. Soc.* **1970**, *92*, 2472–2475.

[8] L. K. Sydnes, E. Bakstad, *Acta Chem. Scand.* **1996**, *50*, 446–453.

[9] P. J. K. and S. D. Crawford, *J. Org. Chem.* **1994**, *59*, 3102–3112.

[10] A. N. Baumann, A. Music, K. Karaghiosoff, D. Didier, *Chem. Commun.* **2016**, *52*, 2529–2532.

[11] A. J. Winter, M. T. Rowe, A. N. M. Weir, N. Akter, S. Z. Mbatha, P. D. Walker, C. Williams, Z. Song, P. R. Race, C. L. Willis, M. P. Crump, *Angew. Chemie - Int. Ed.* **2022**, *61*, 1–10.

[12] T. Fortunati, M. D’Acunto, T. Caruso, A. Spinella, *Tetrahedron* **2015**, *71*, 2357–2362.

[13] E. Dunny, P. Evans, *J. Org. Chem.* **2010**, *75*, 5334–5336.

[14] A. R. Offenbacher, H. Zhu, J. P. Klinman, *Tetrahedron Lett.* **2016**, *57*, 4537–4540.

[15] M. Serhan, M. Sprowls, D. Jackemeyer, M. Long, I. D. Perez, W. Maret, N. Tao, E. Forzani, *Org. Biomol. Chem.* **2017**, *15*, 4308–4313.

[16] C. A. Brown, V. K. Ahuja, *J. Org. Chem.* **1973**, *38*, 2226–2230.

[17] Y. Tanabe, Shuhei; Kobayashi, *Org. Biomol. Chem.* **2019**, *17*, 2393–2402.

[18] S. Peng, N. M. Okeley, A. L. Tsai, G. Wu, R. J. Kulmacz, W. A. Van der Donk, *J. Am. Chem. Soc.* **2002**, *124*, 10785–10796.

[19] X. Jiang, J. Zhang, S. Ma, *J. Am. Chem. Soc.* **2016**, *138*, 8344–8347.

[20] S. Thurnhofer, W. Vetter, *Tetrahedron* **2007**, *63*, 1140–1145.

[21] S. M. Rafferty, J. E. Rutherford, L. Zhang, L. Wang, D. A. Nagib, *J. Am. Chem. Soc.* **2021**, *143*, 5622–5628.

[22] F. Rui, W. Boland, *J. Org. Chem.* **2010**, *75*, 3958–3964.

[23] W. J. Gensler, M. B. Floyd, R. Yanase, K. W. Pober, *J. Am. Chem. Soc.* **1970**, *92*, 2472–2475.

## 2-bromohept-1-ene (11)

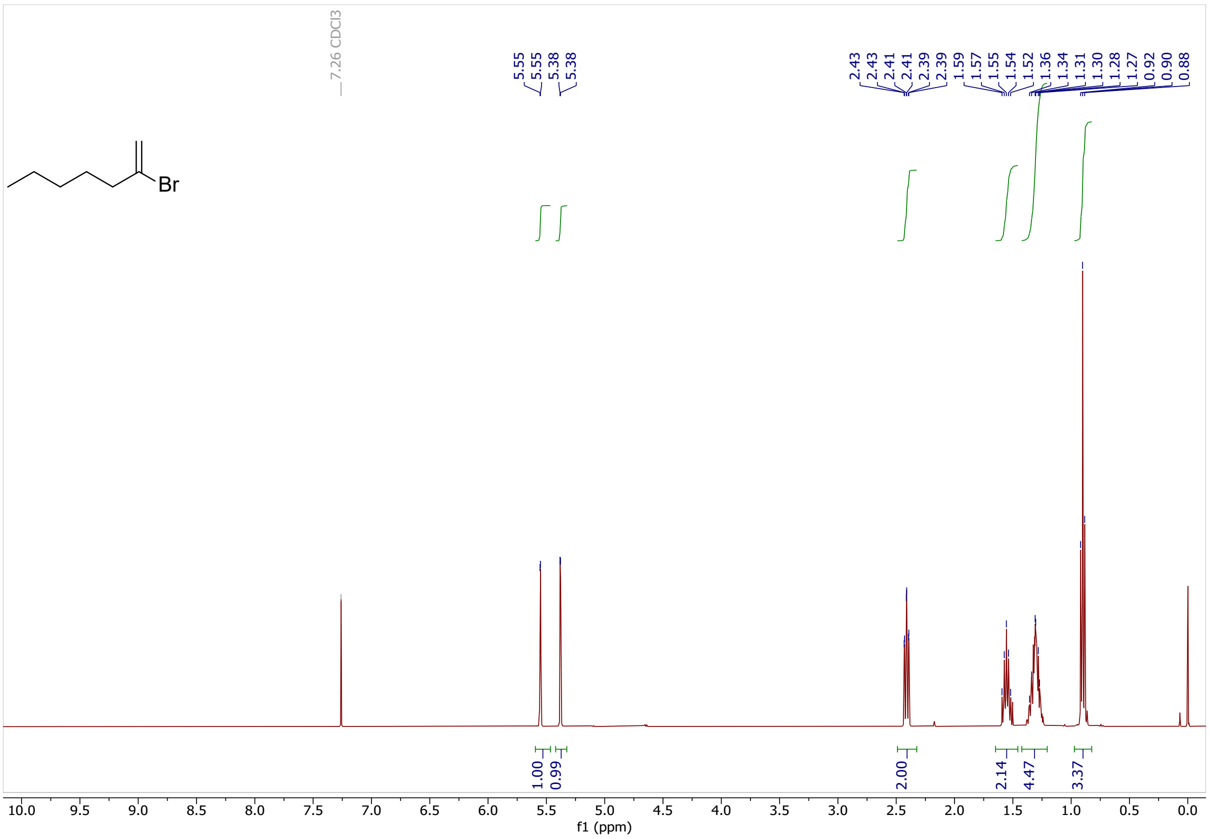

## 1,1,2-tribromo-2-pentylcyclopropane (12)

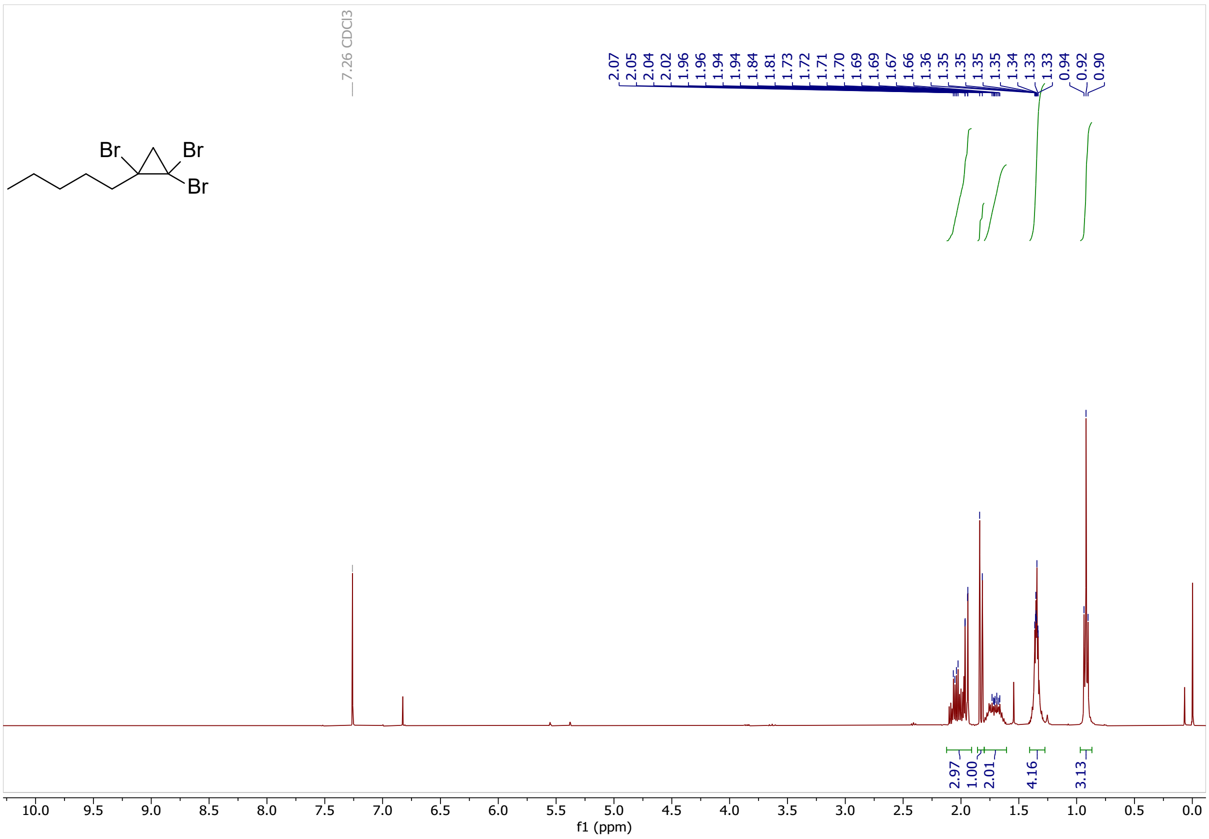

**2-(2-pentylcycloprop-1-en-1-yl)ethan-1-ol (13)**

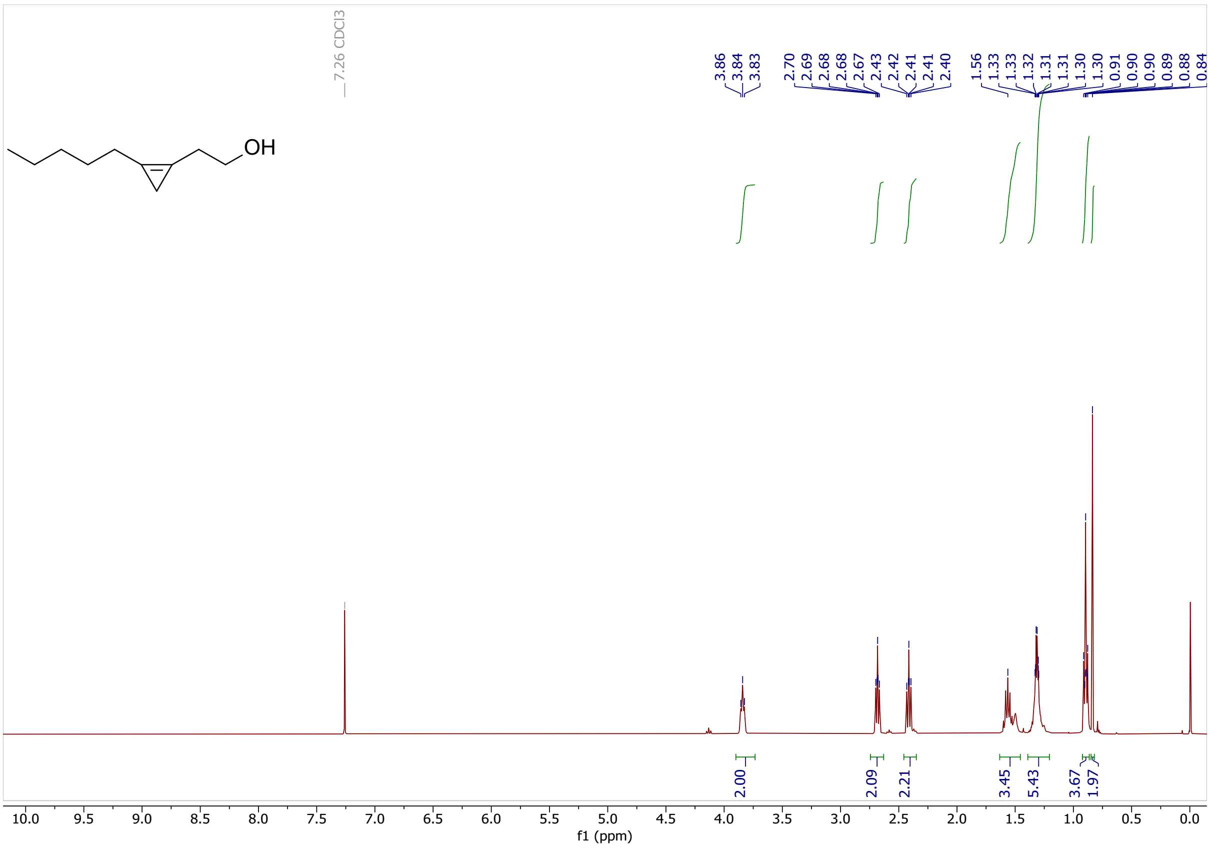

## 1-(2-bromoethyl)-2-pentylcycloprop-1-ene (14)

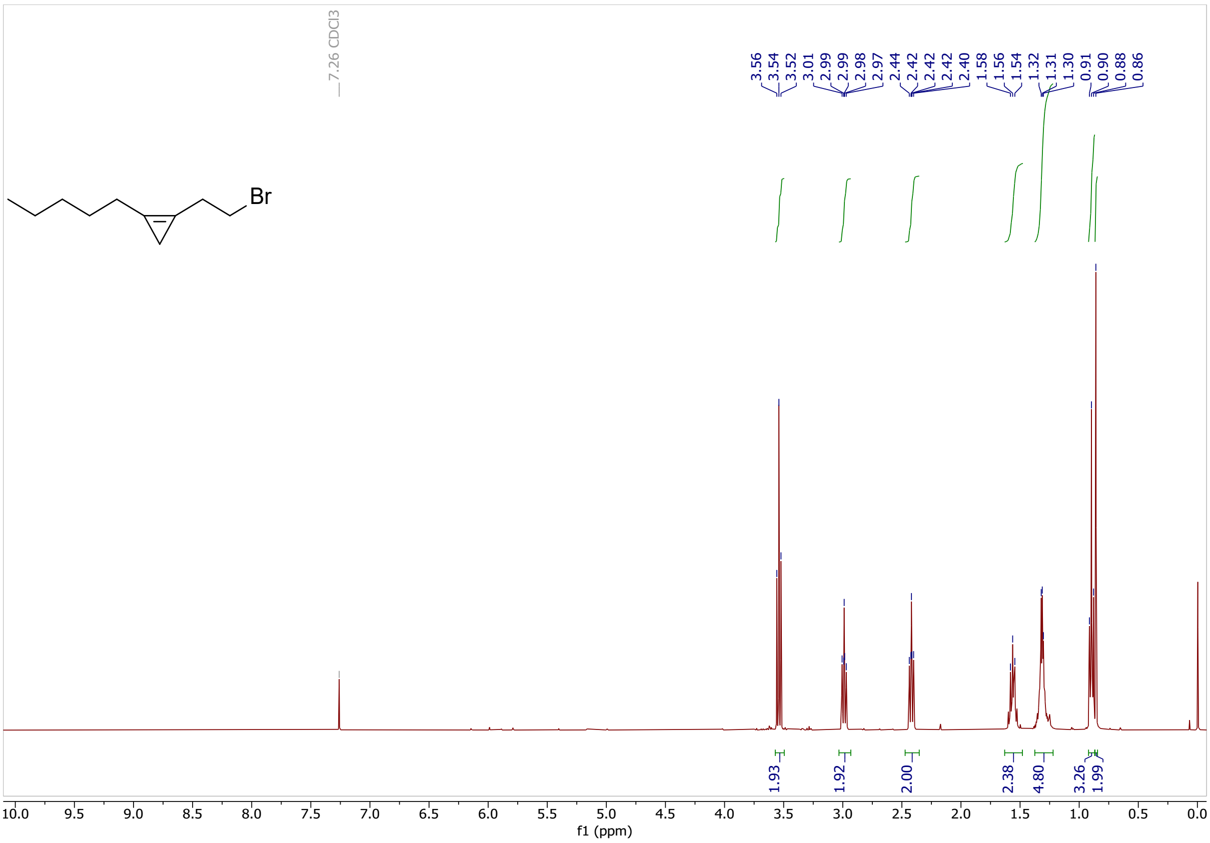


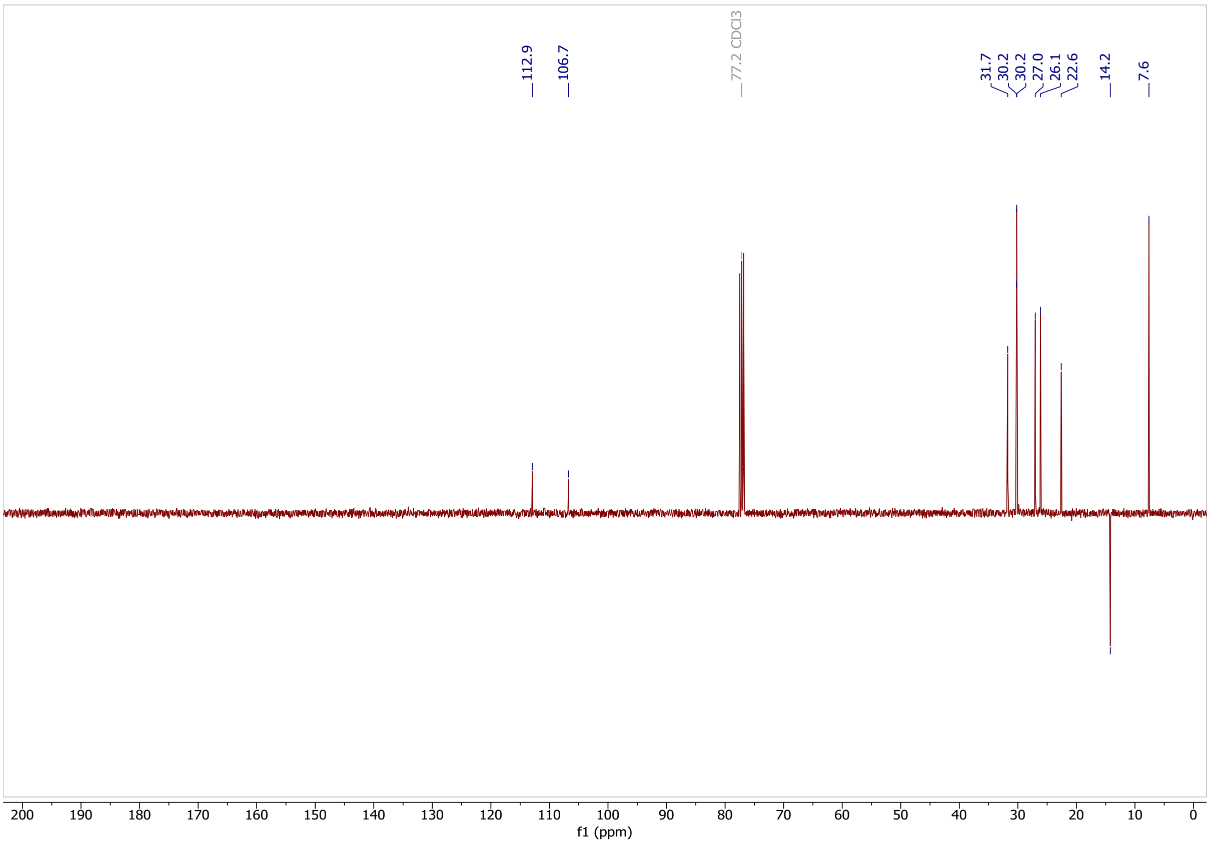

**(2-(2-pentylcycloprop-1-en-1-yl)ethyl)triphenylphosphonium bromide (7)**

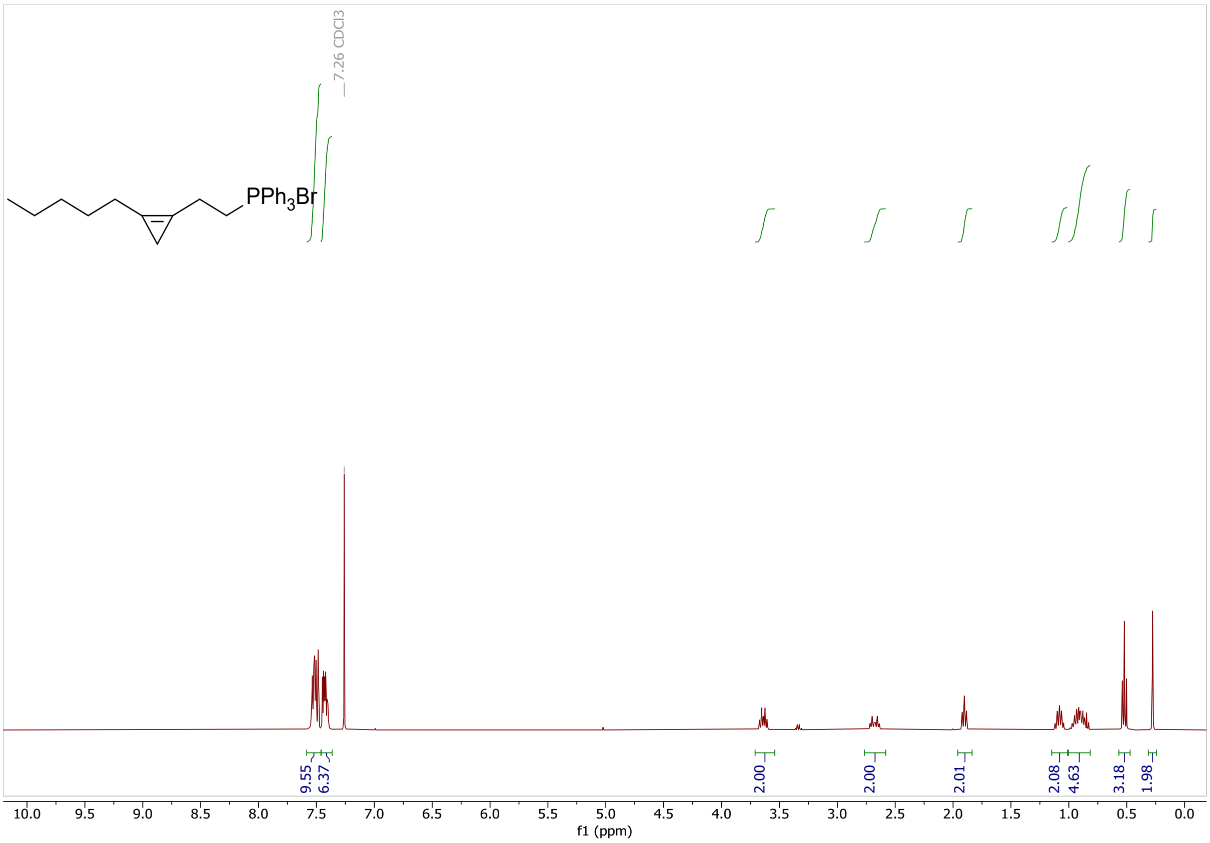


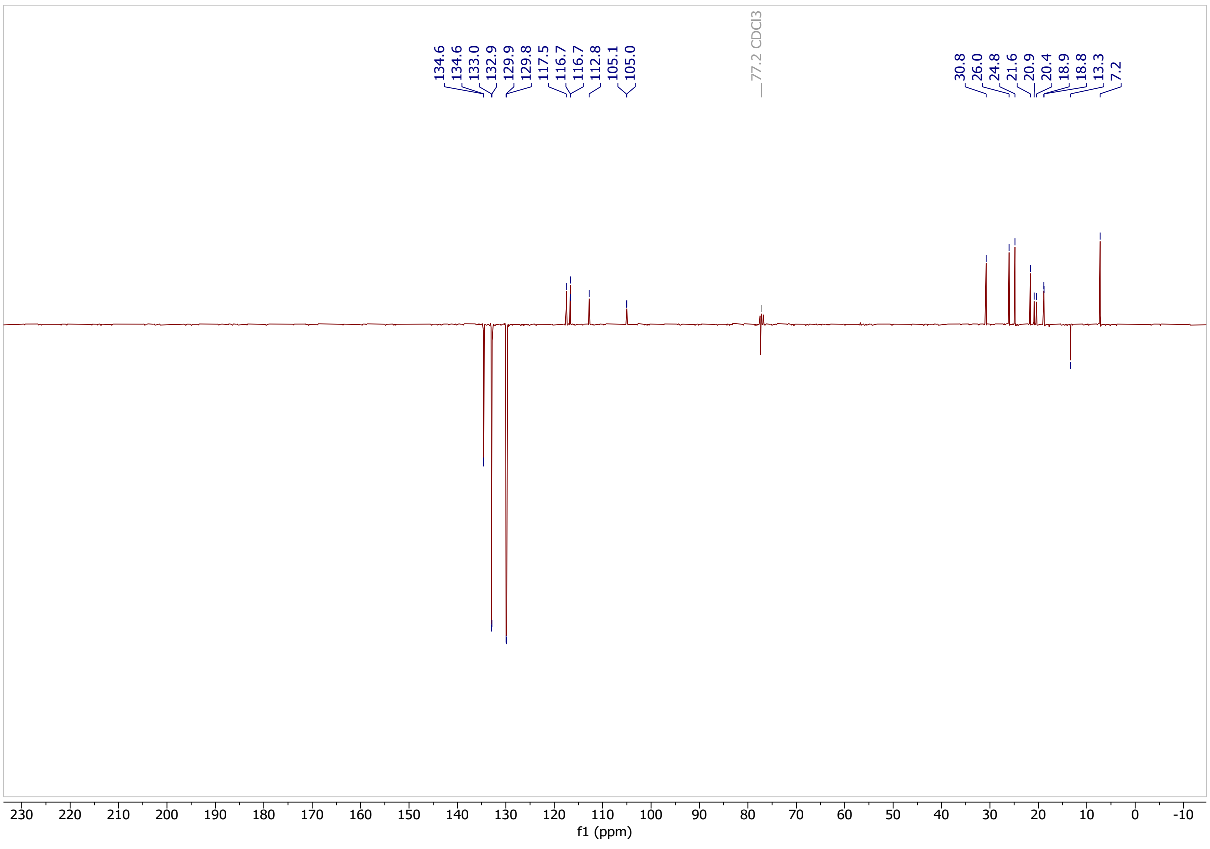

## 9-((tert-butyldimethylsilyl)oxy)nonan-1-ol (14)

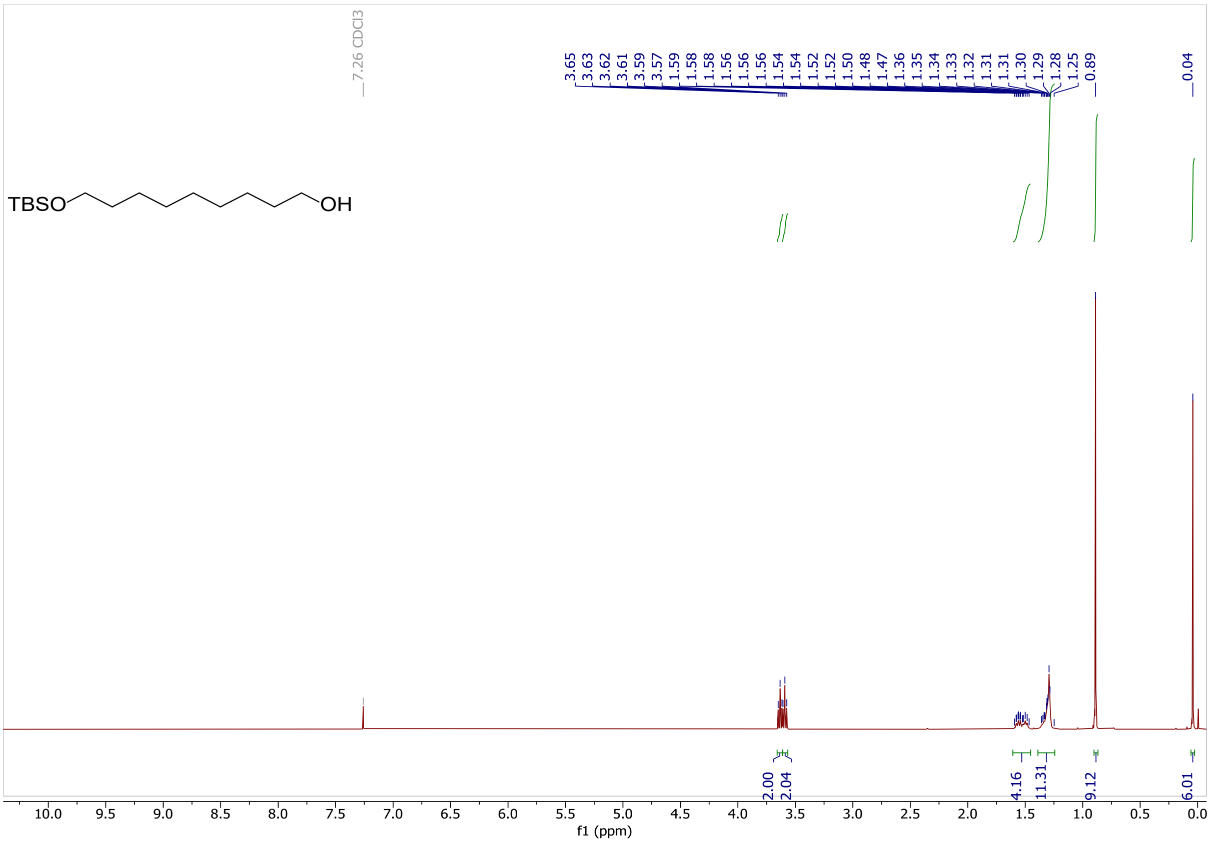


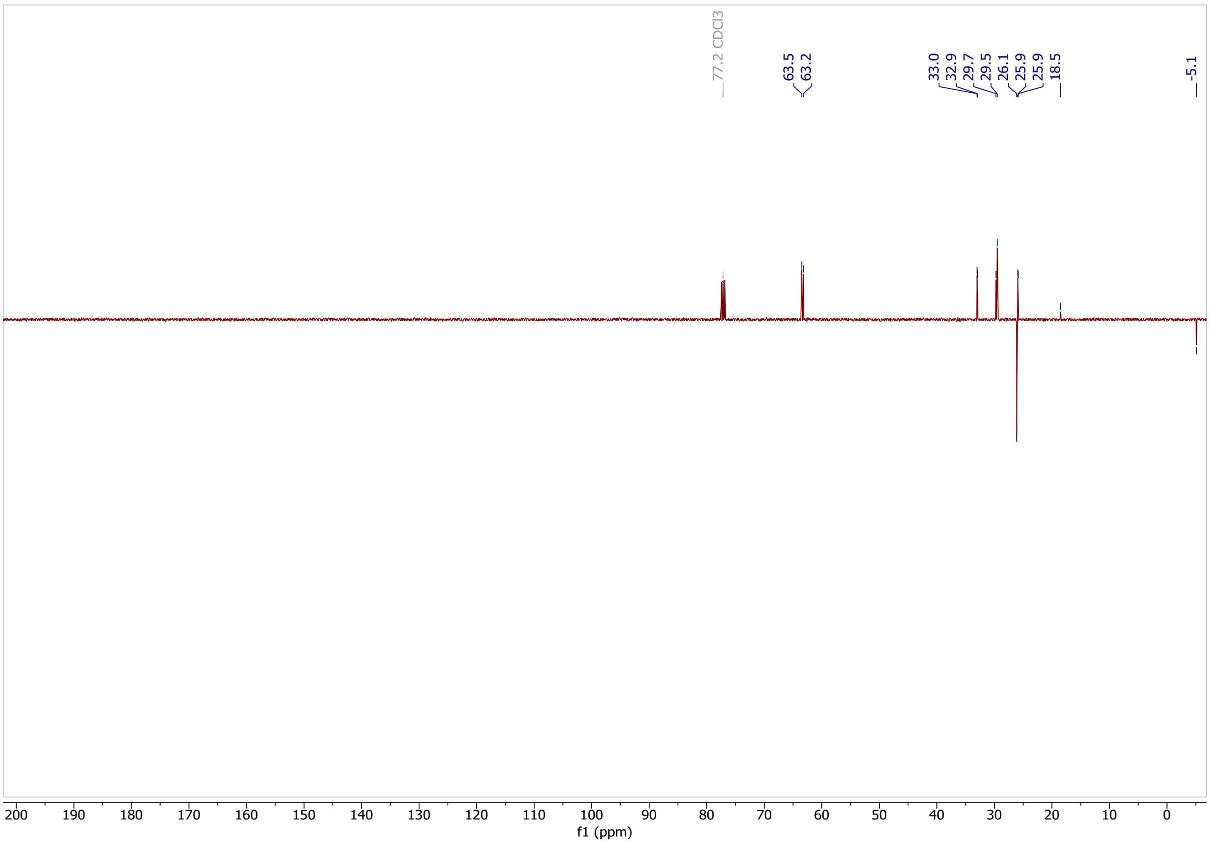

**9-((tert-butyldimethylsilyl)oxy)nonanoic acid (15)**

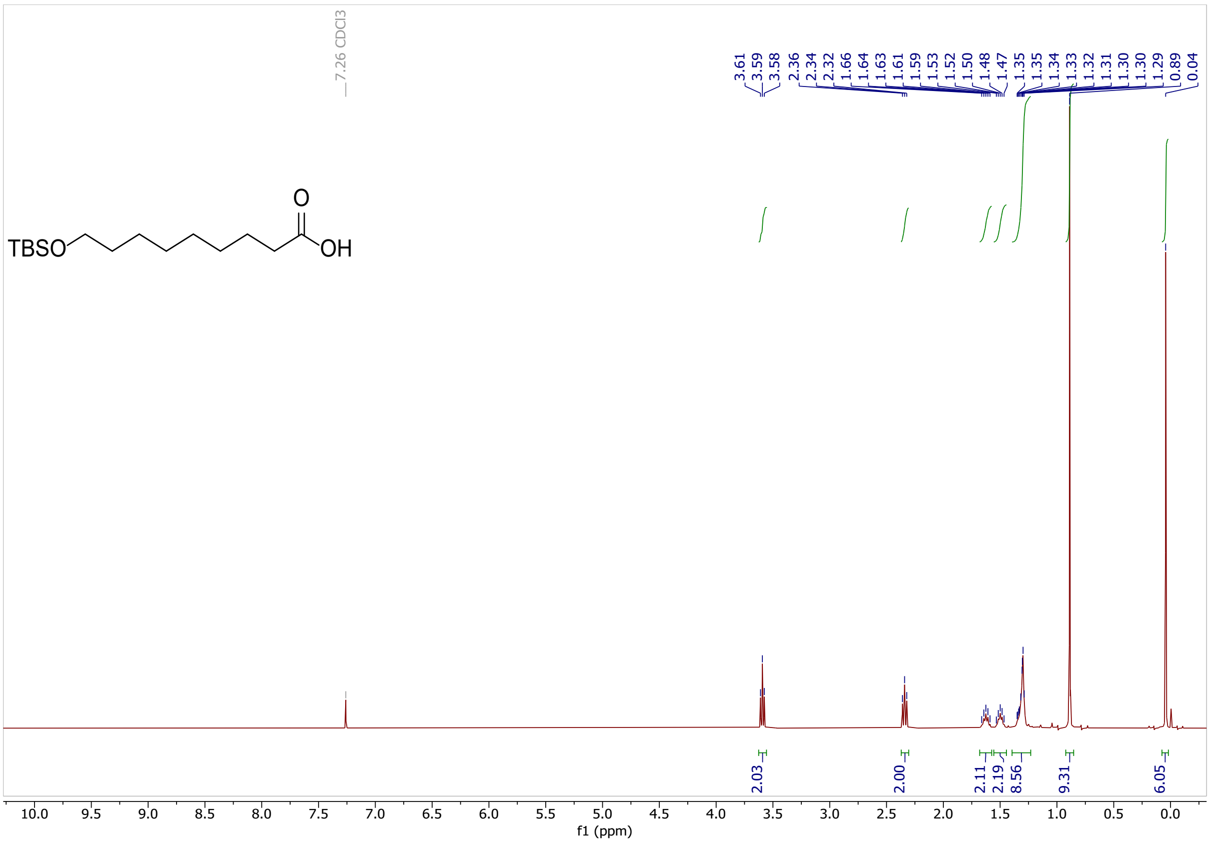


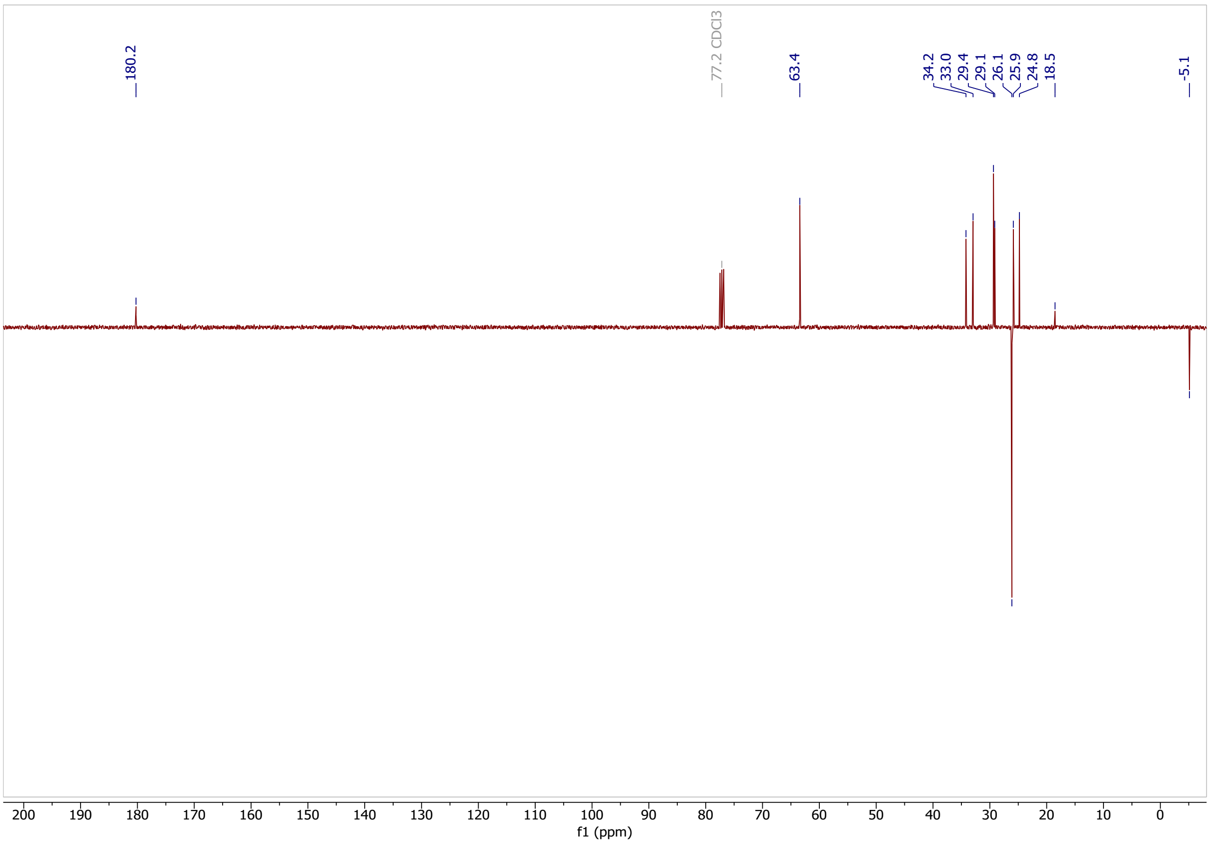

**methyl 9-((tert-butyldimethylsilyl)oxy)nonanoate (16)**

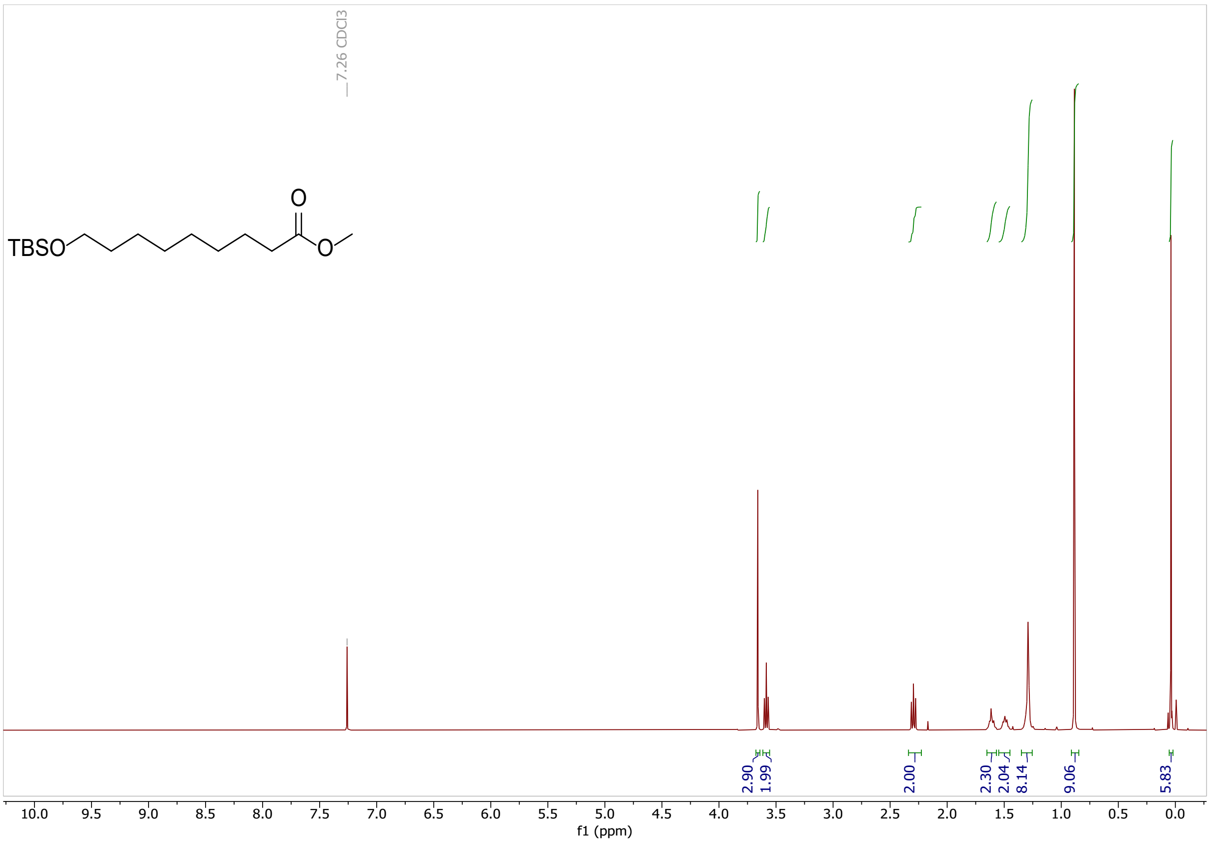


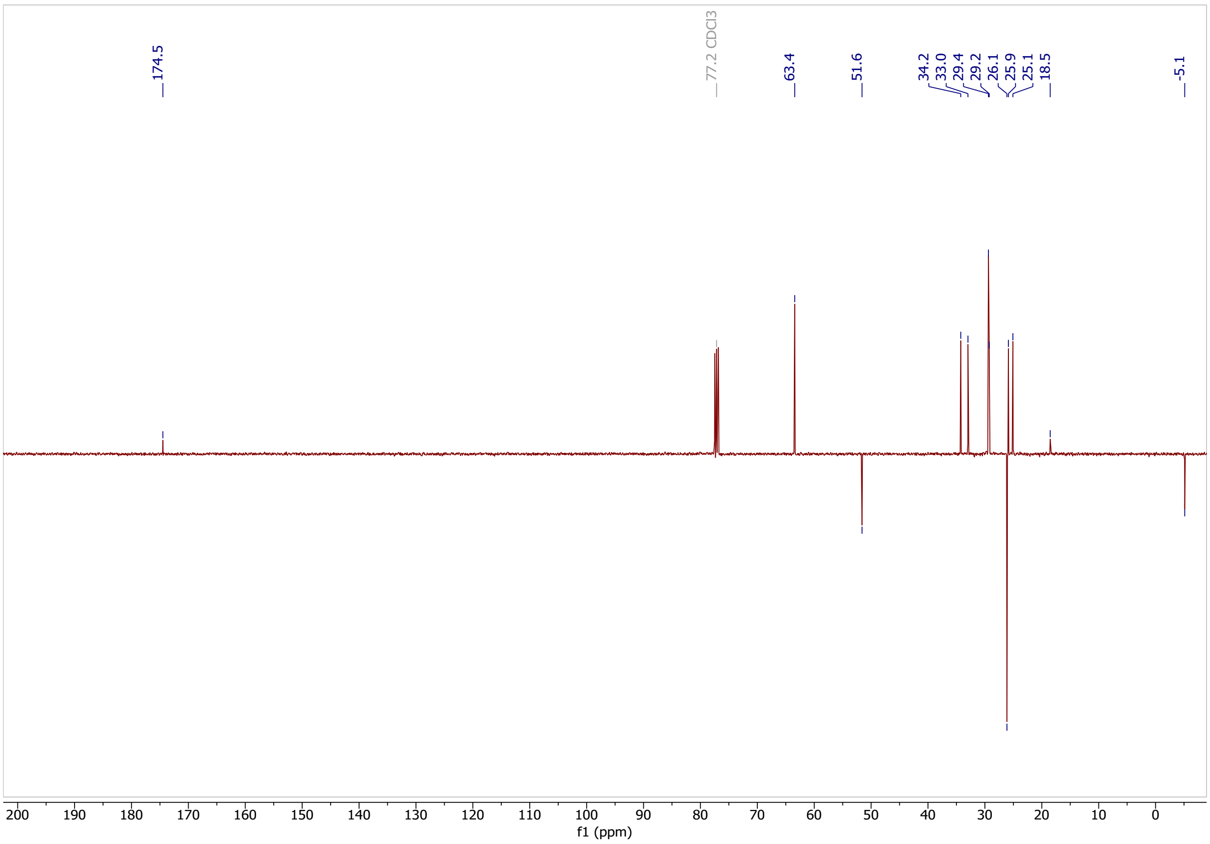

**methyl 9-hydroxynonanoate (17)**

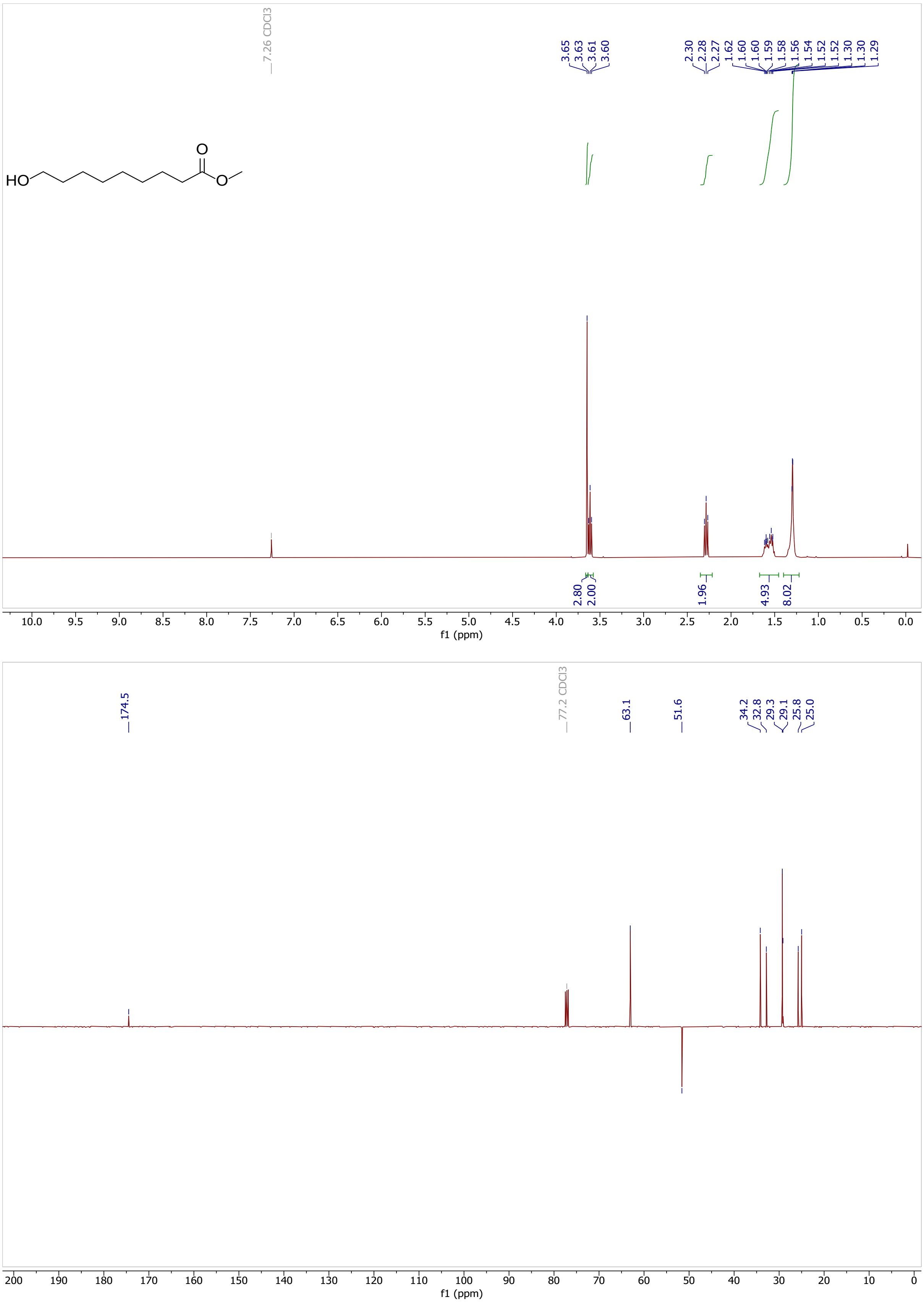

**methyl 9-oxononanoate (8)**

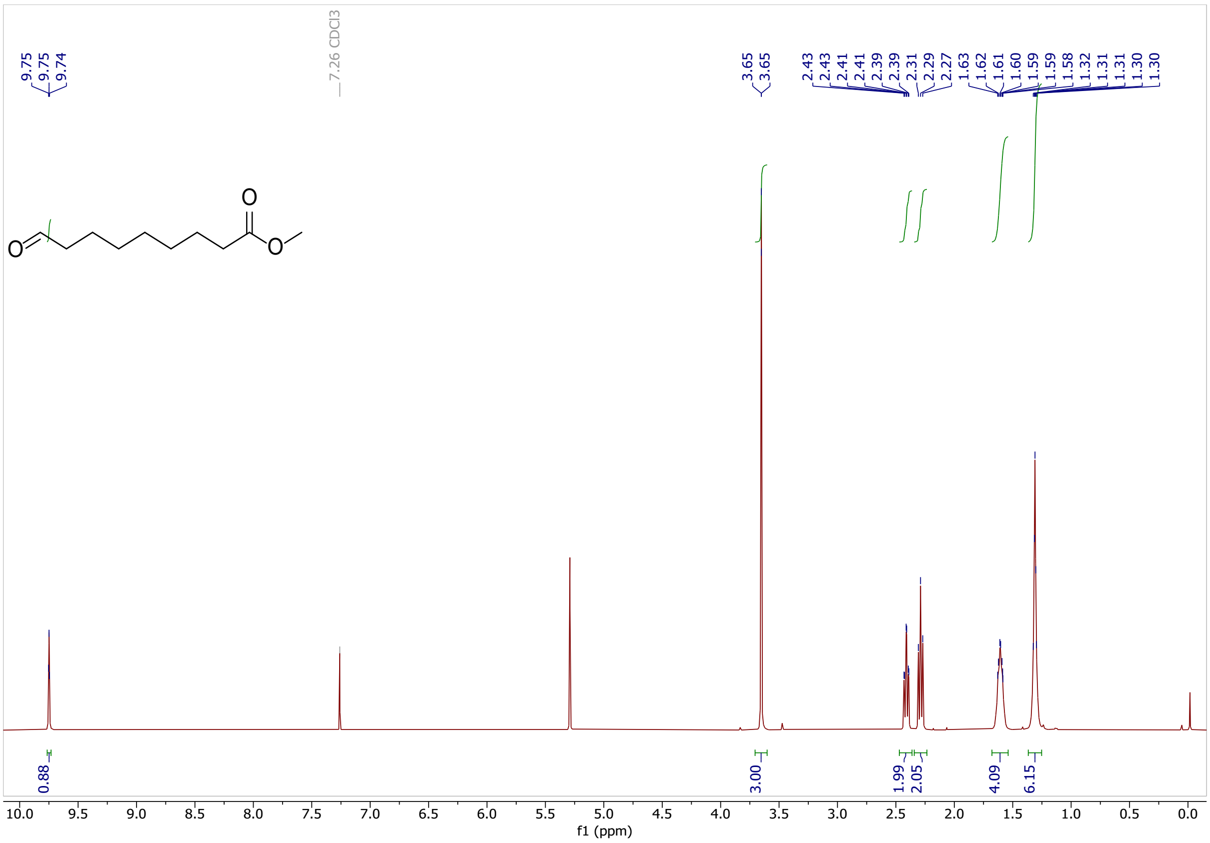


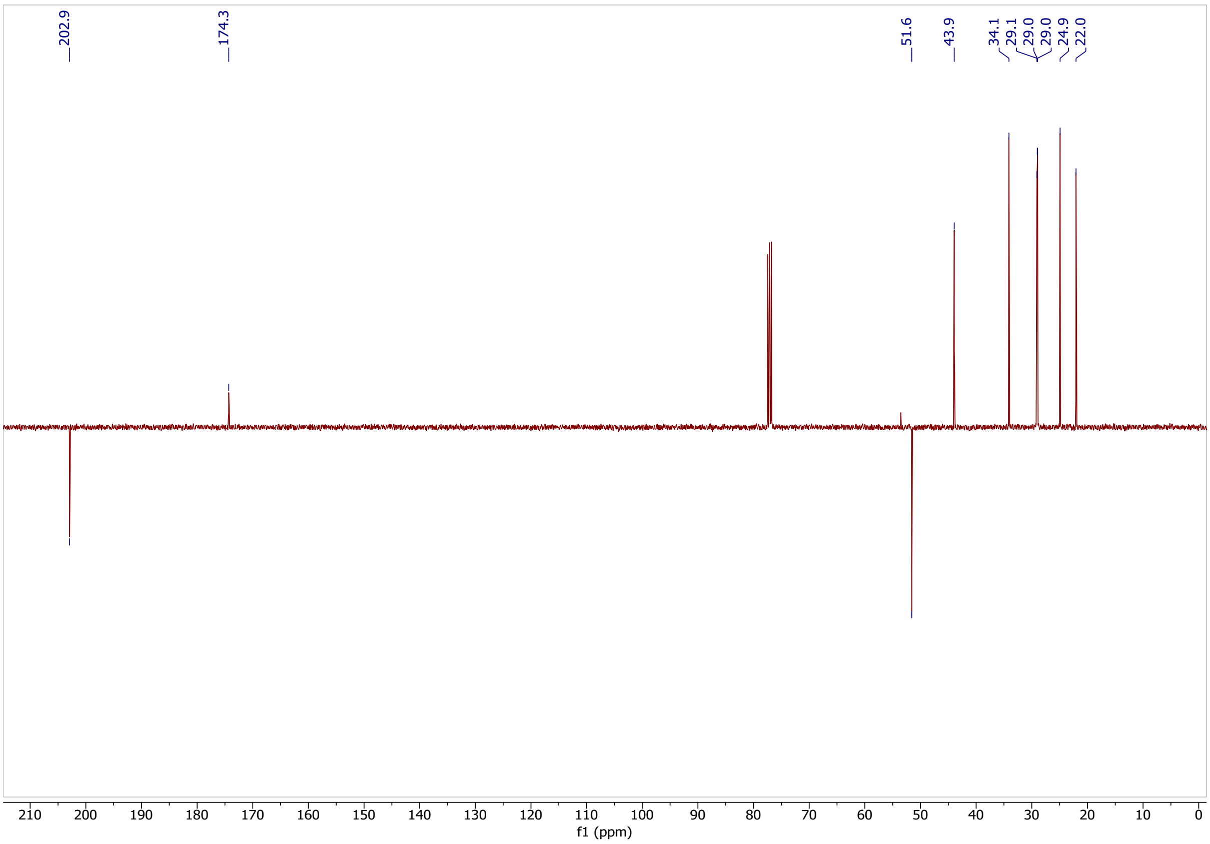

## methyl (Z)-11-(2-pentylcycloprop-1-en-1-yl)undec-9-enoate (18)

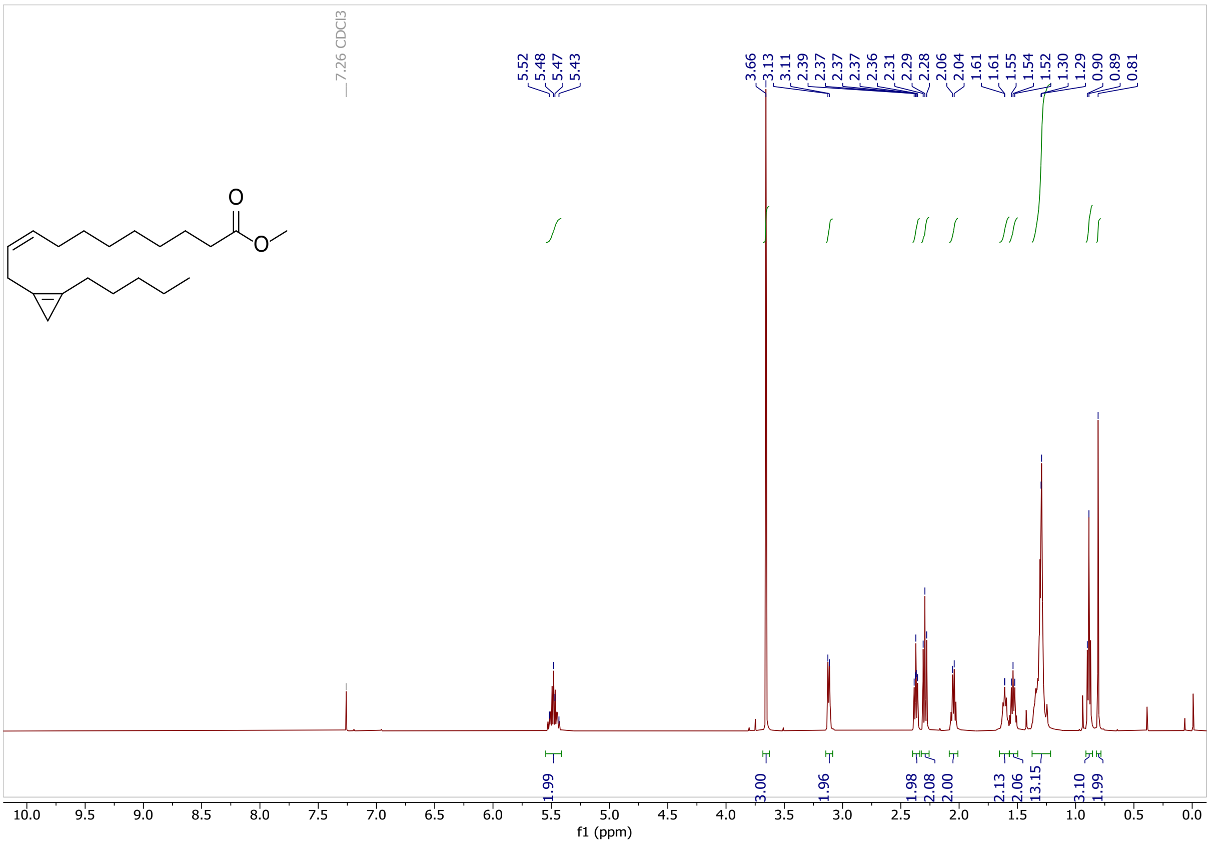


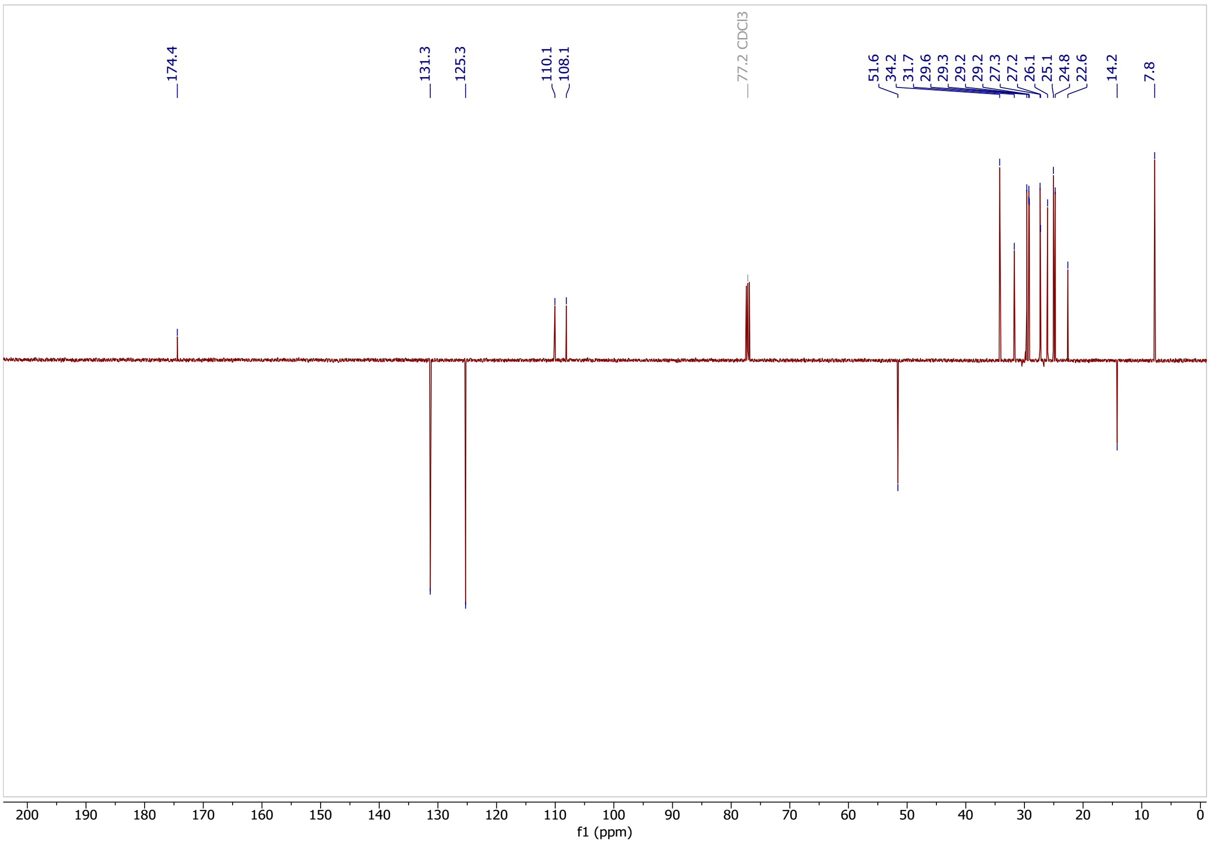

**(Z)-11-(2-pentylcycloprop-1-en-1-yl)undec-9-enoic acid (5)**

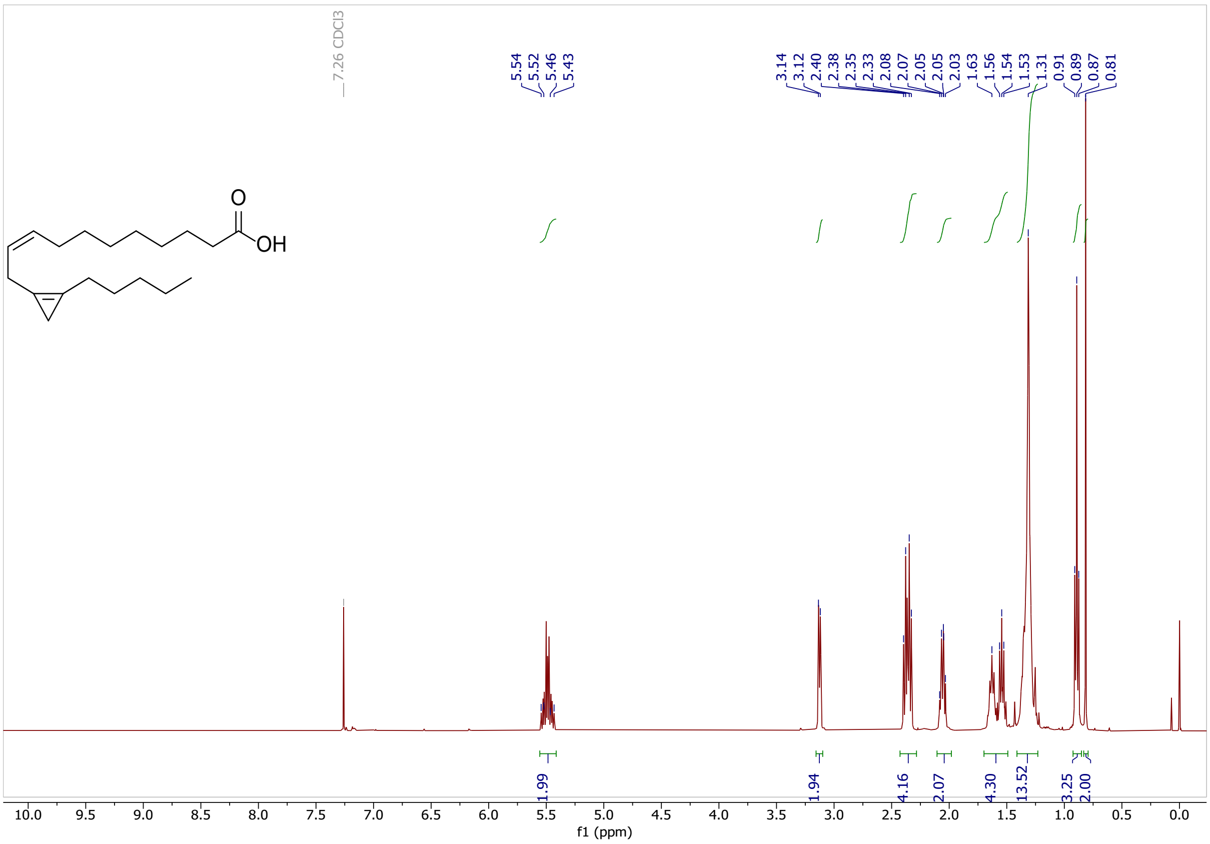


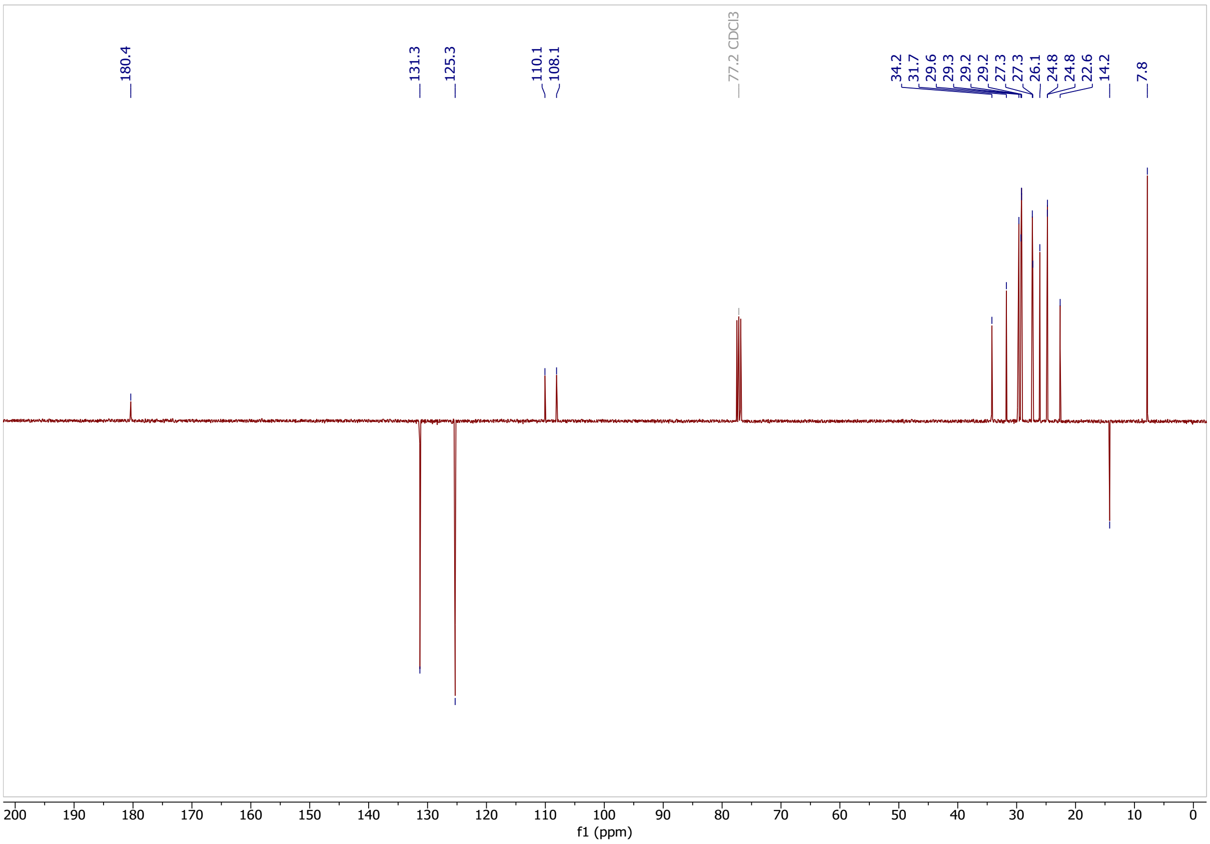

## methyl 7-hydroxyhept-5-ynoate (19)

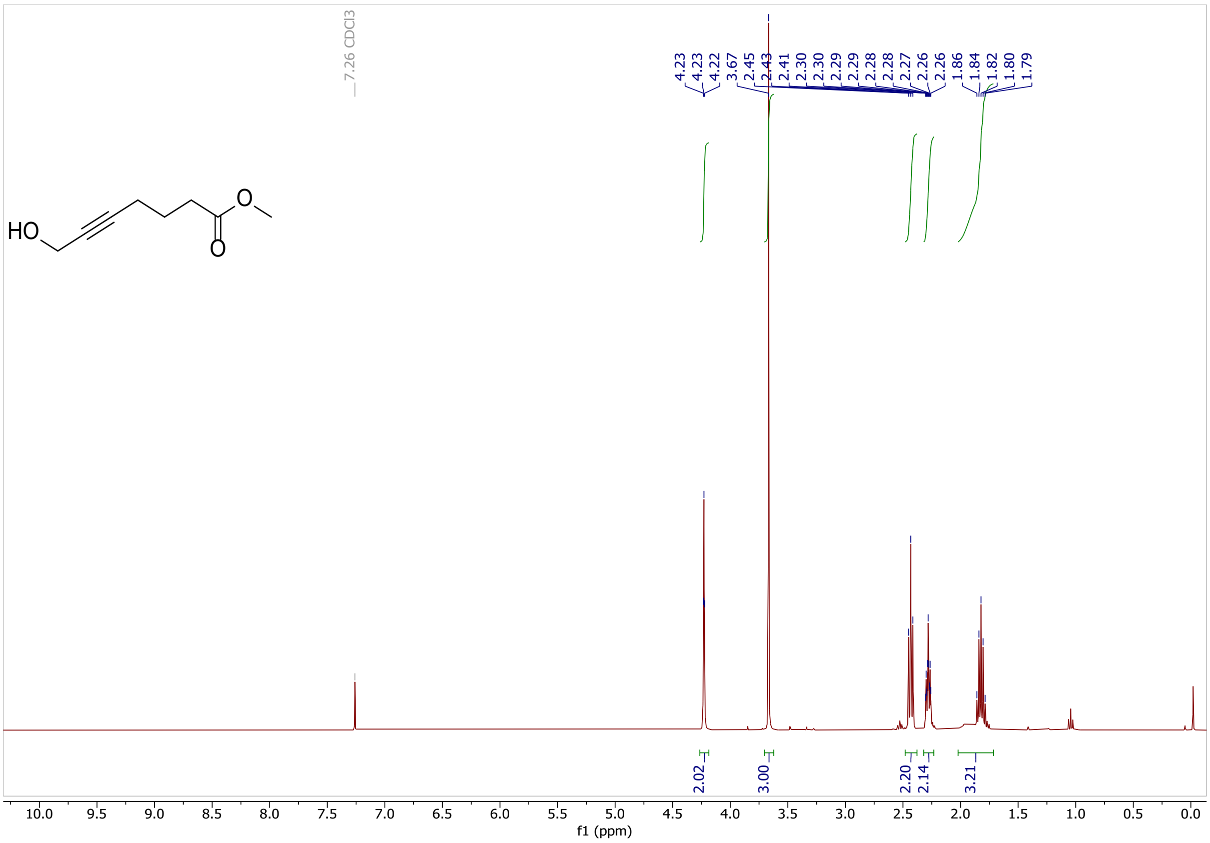


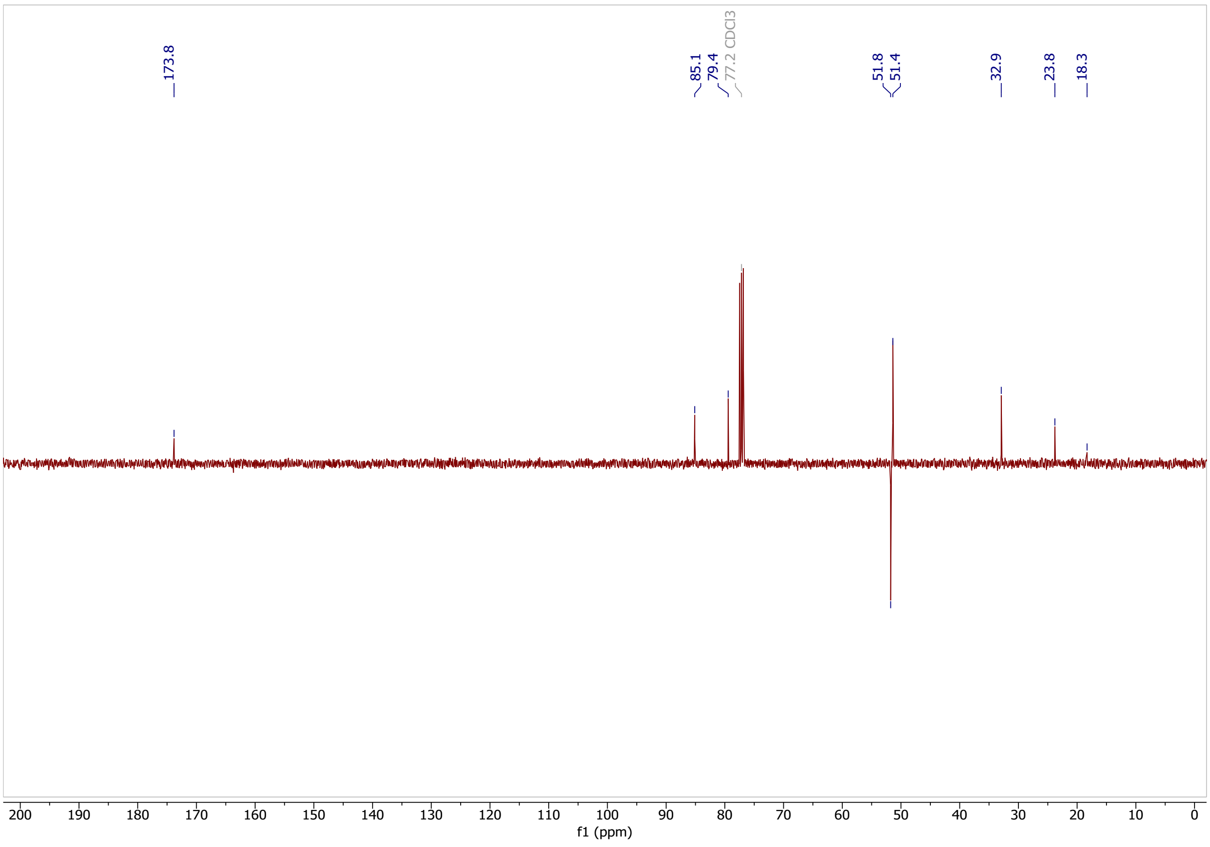

**methyl 7-bromohept-5-ynoate (20)**

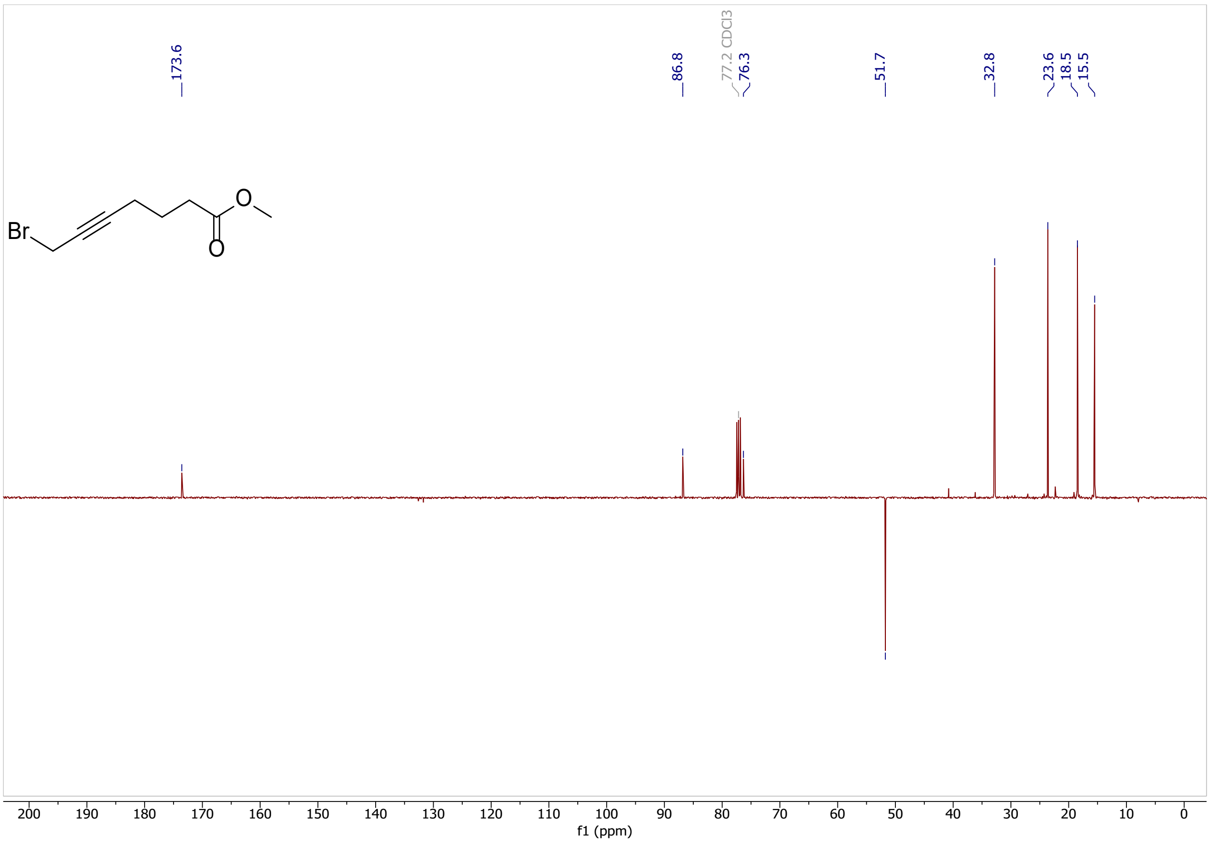


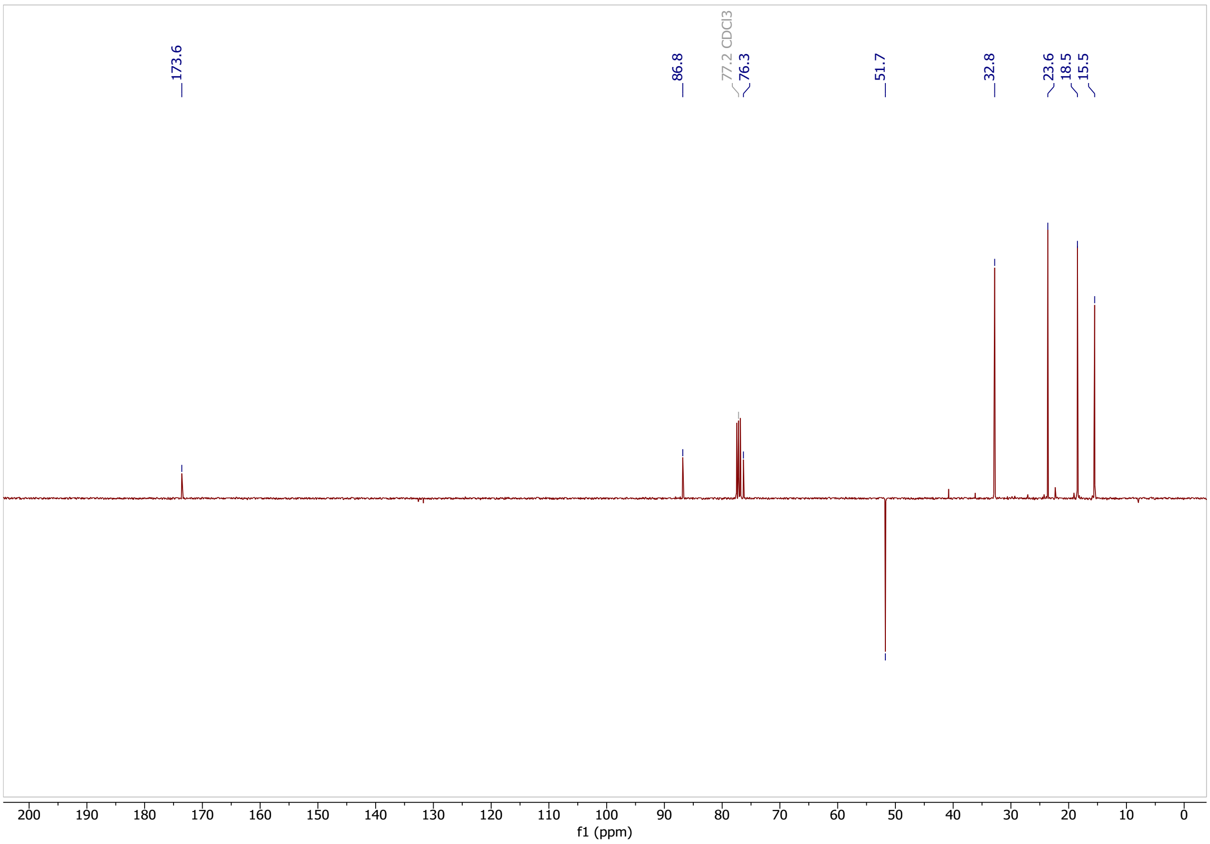

**methyl 11-hydroxyundeca-5,8-diynoate (10)**

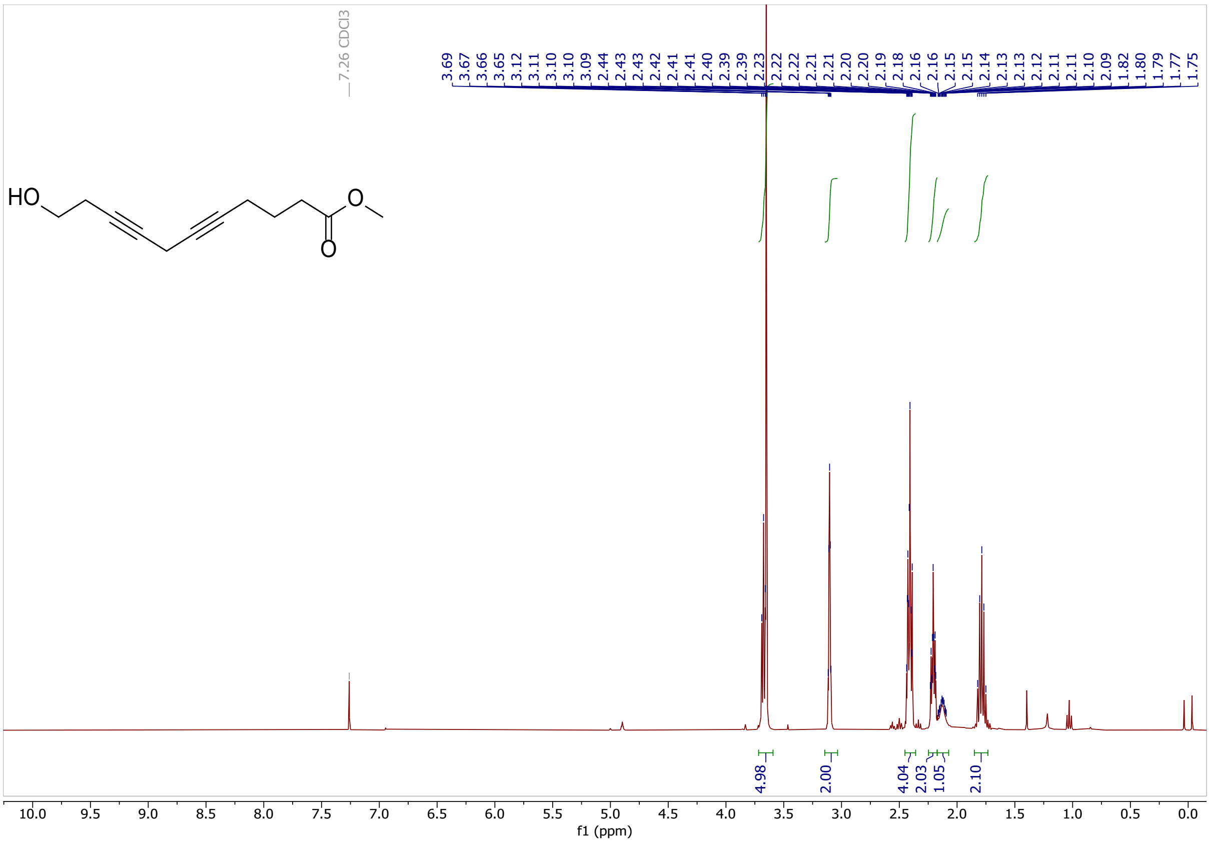

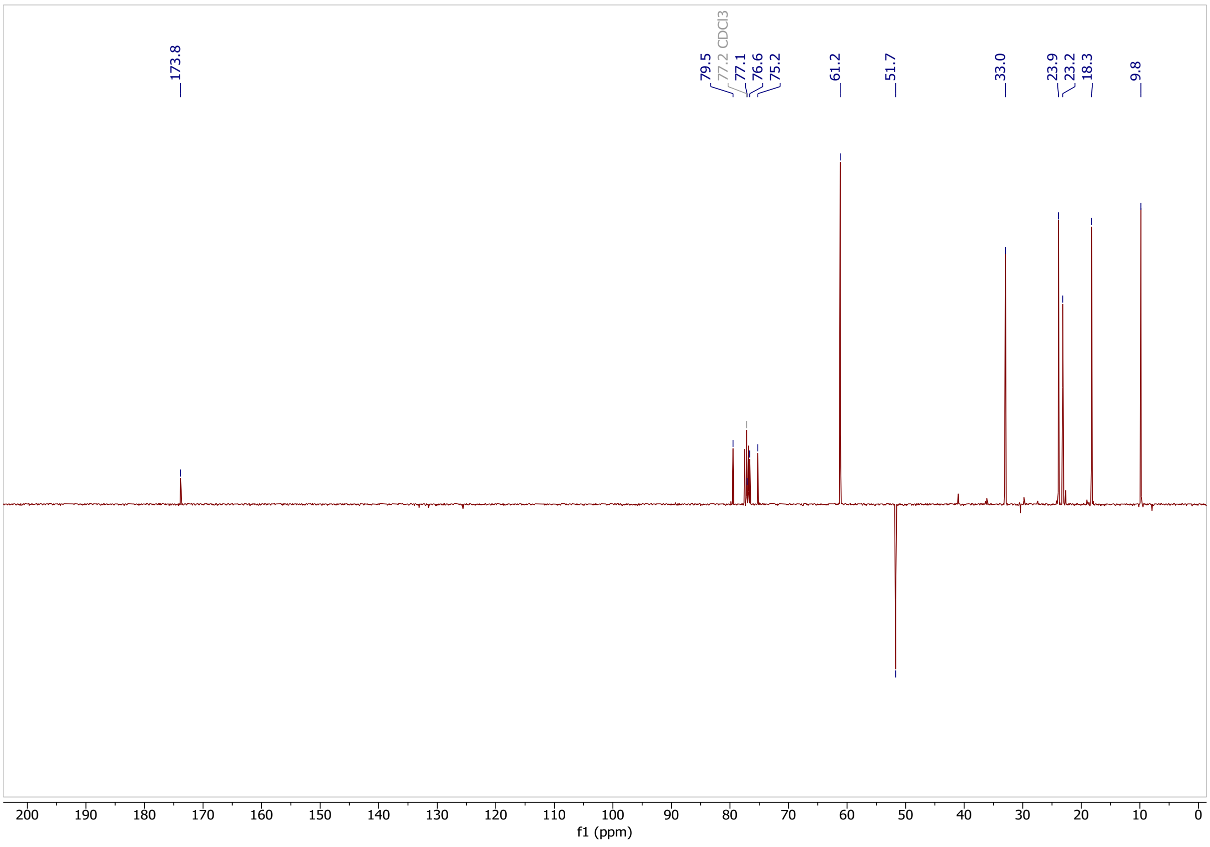

**methyl (5Z,8Z)-11-hydroxyundeca-5,8-dienoate (21)**

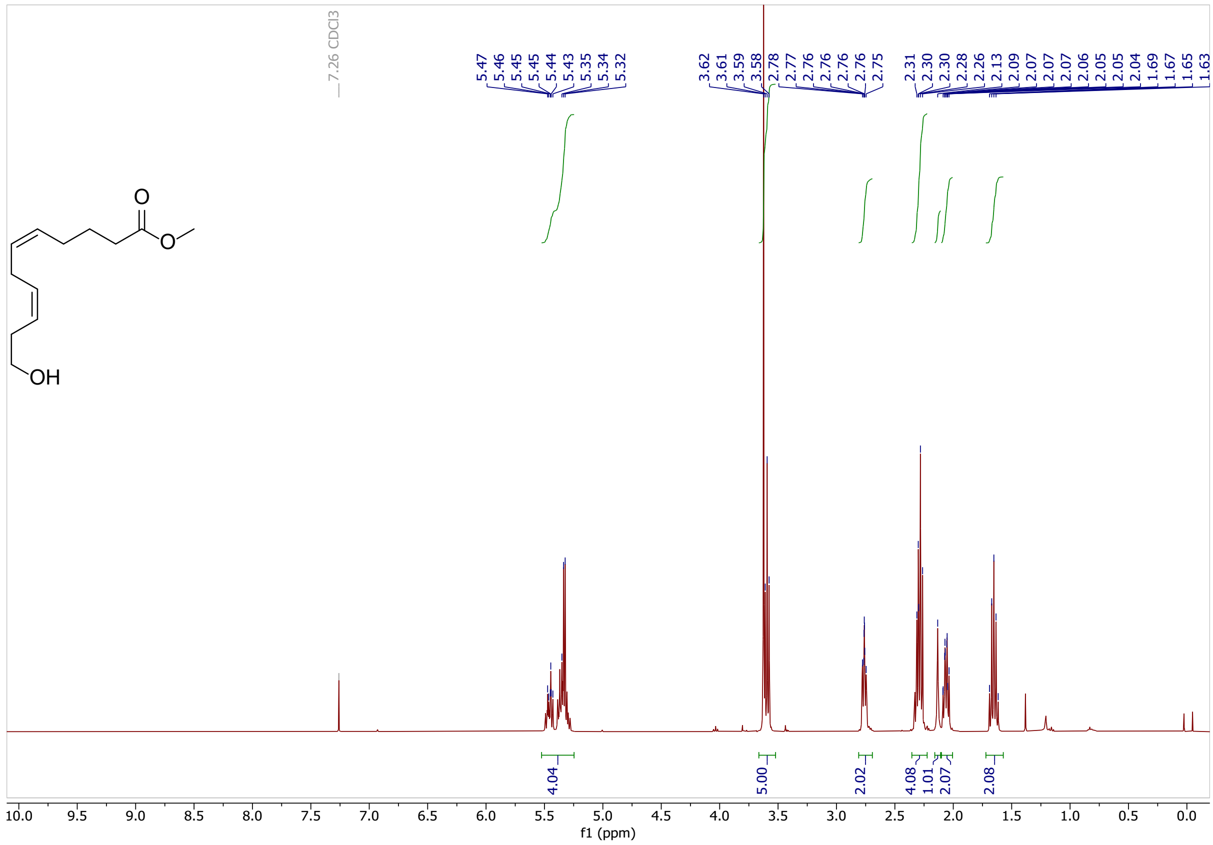


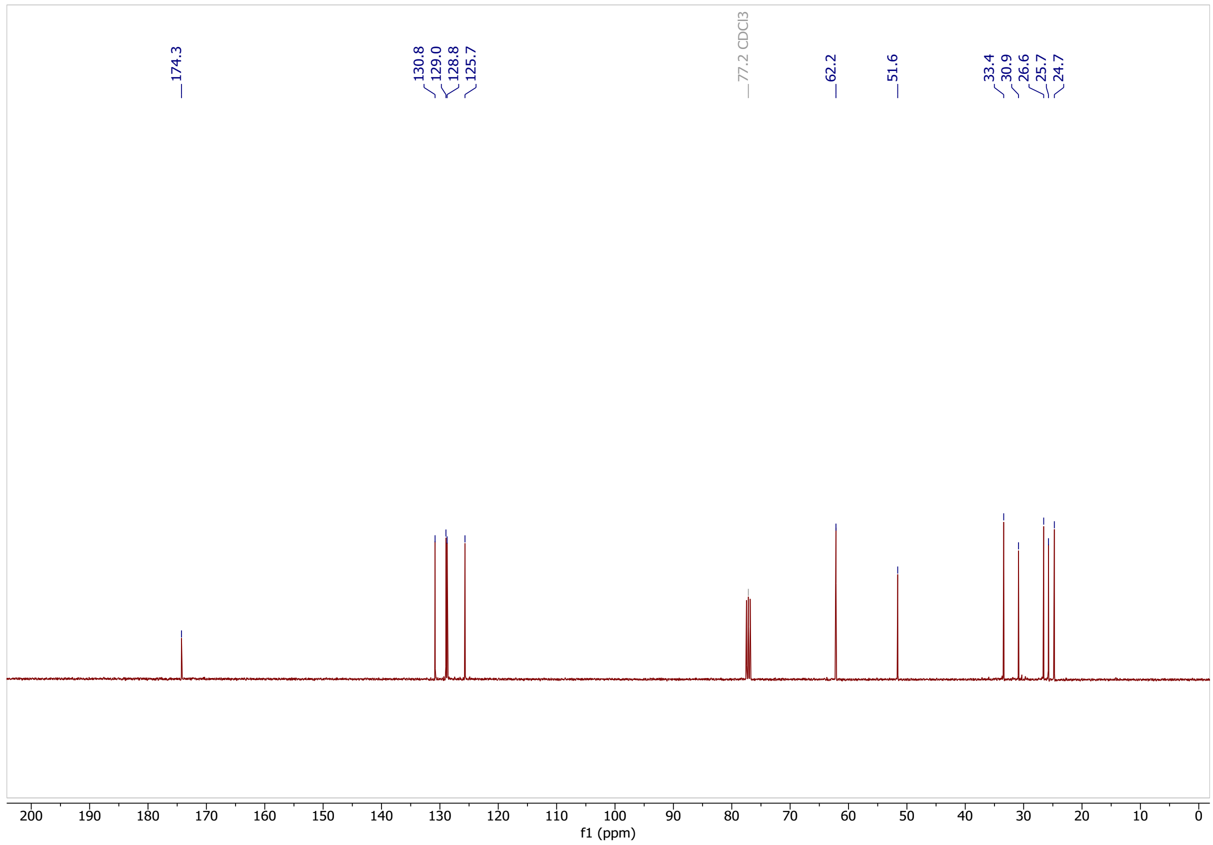

**methyl (5Z,8Z)-11-oxoundeca-5,8-dienoate (9)**

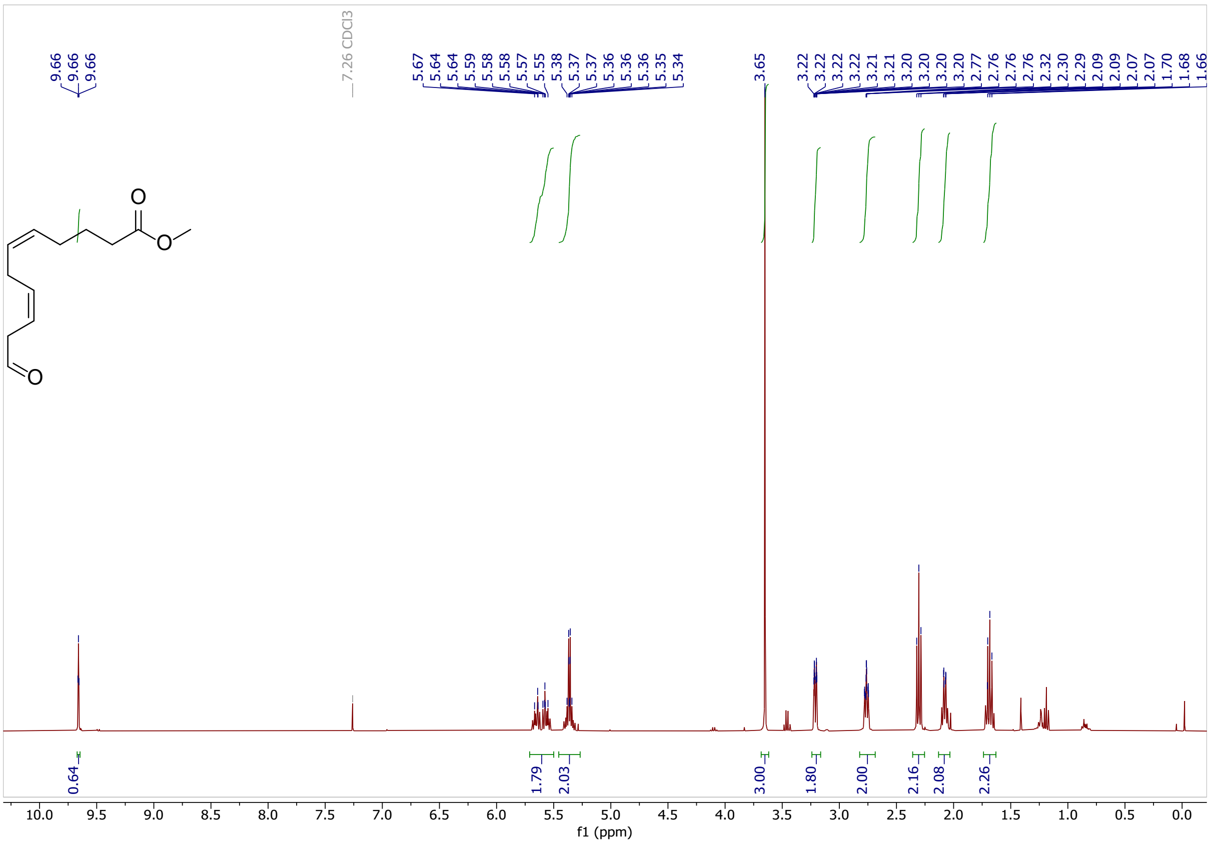

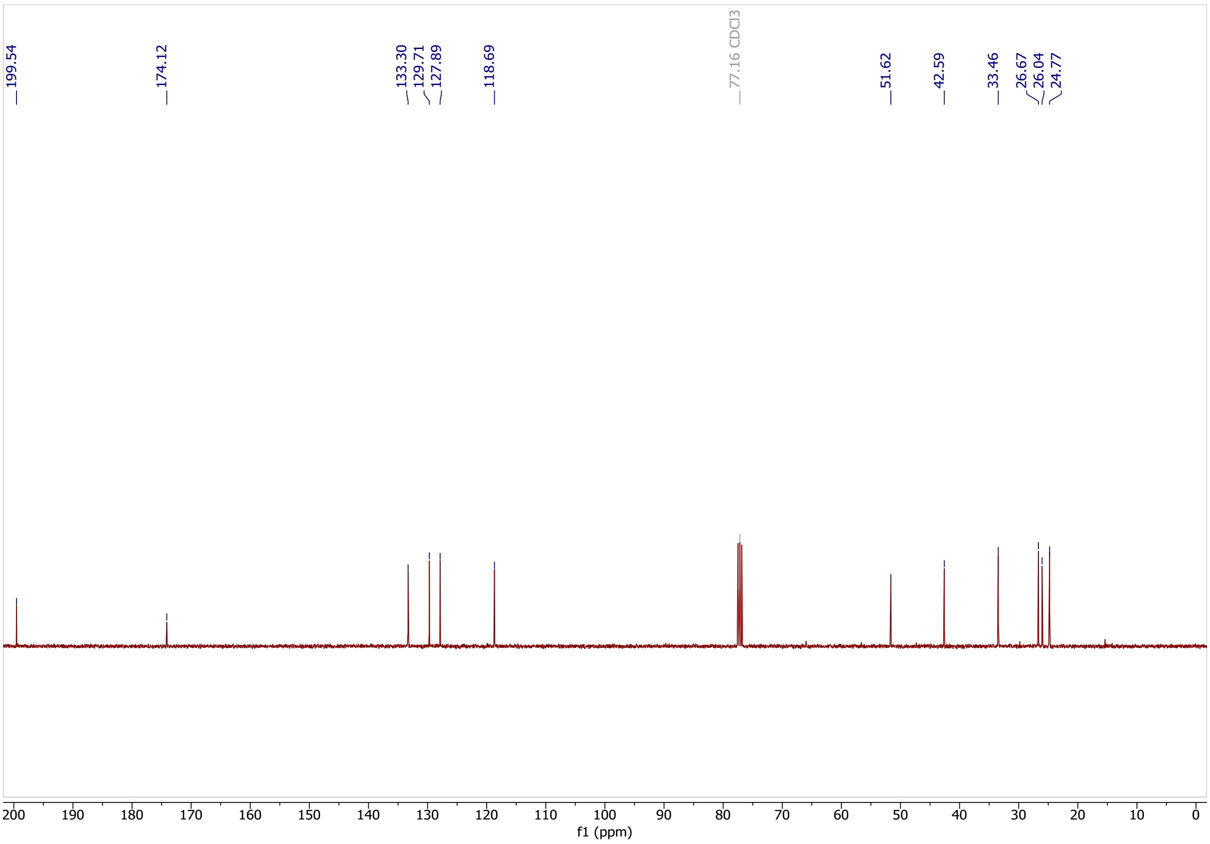

## methyl (5Z,8Z,11Z)-13-(2-pentylcycloprop-1-en-1-yl)trideca-5,8,11-trienoate (22)

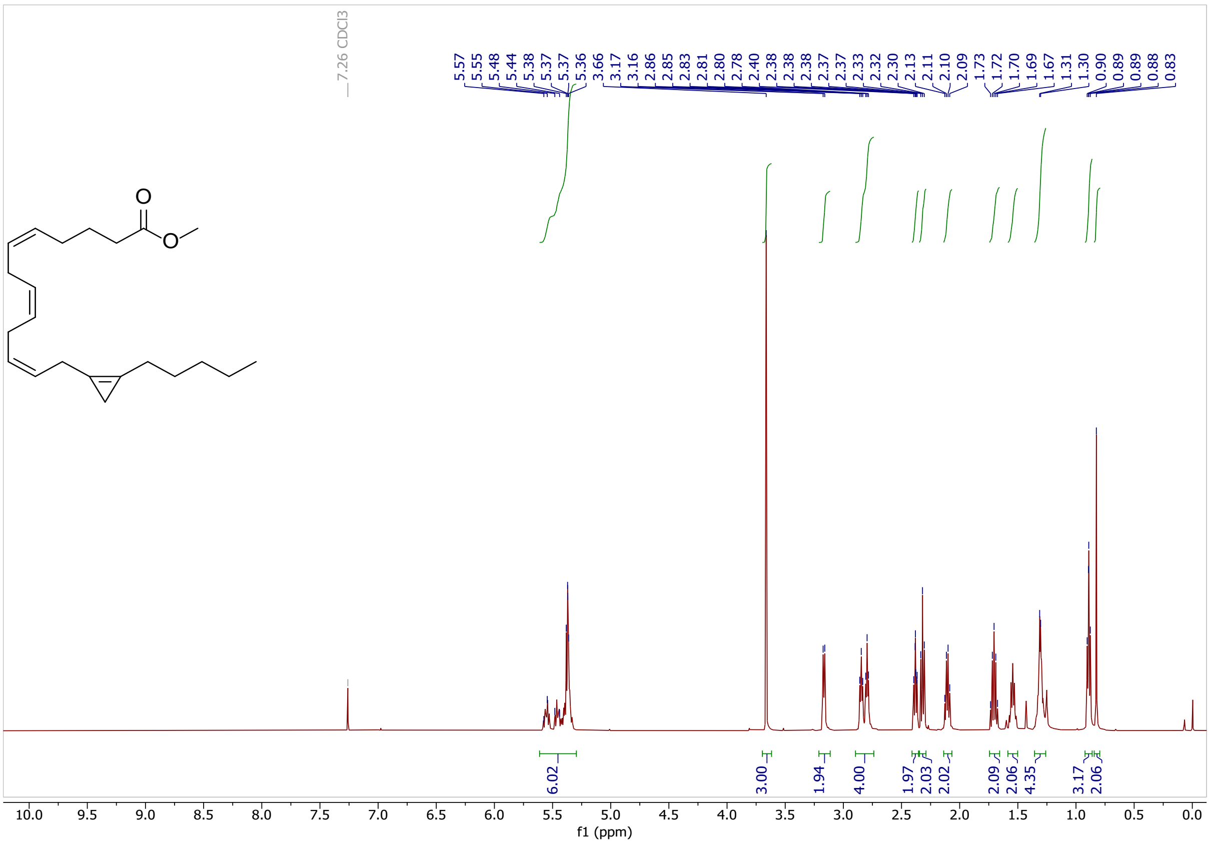

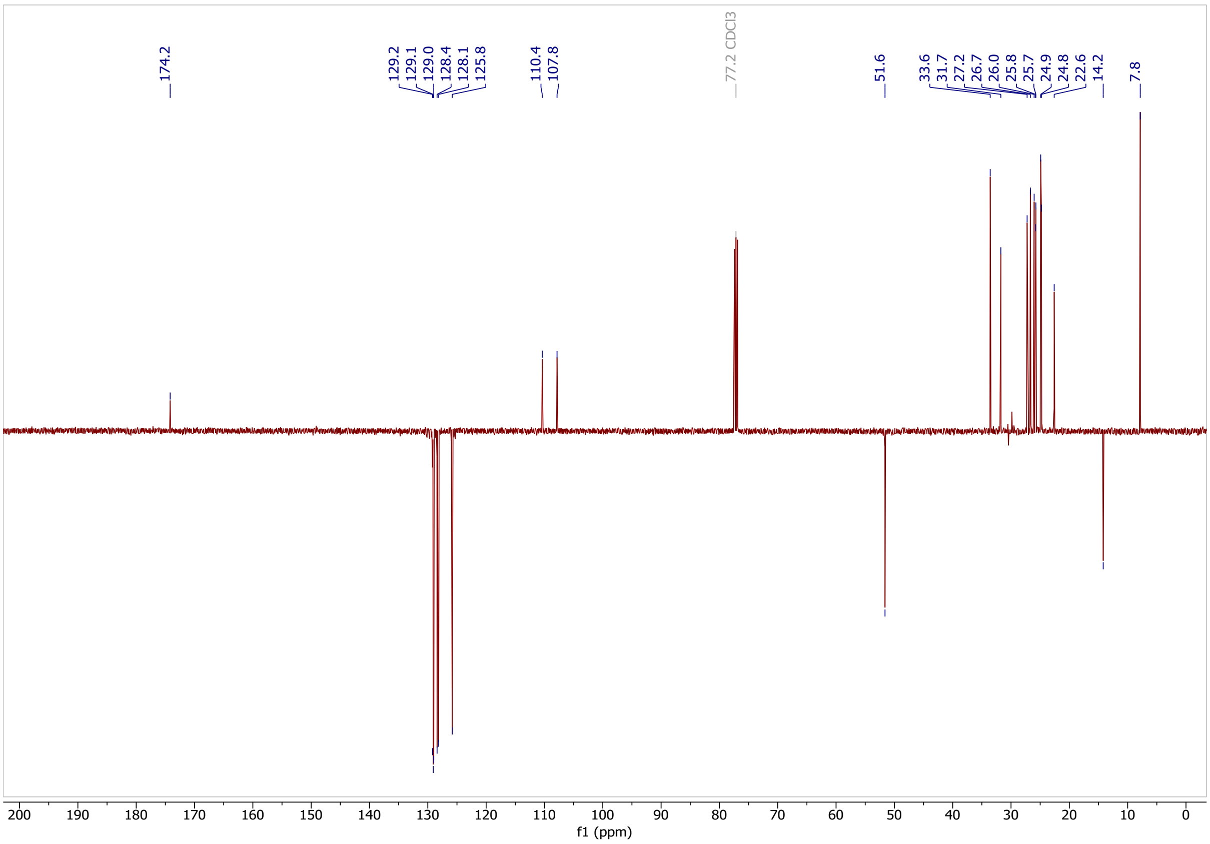

## (5Z,8Z,11Z)-13-(2-pentylcycloprop-1-en-1-yl)trideca-5,8,11-trienoic acid (6)

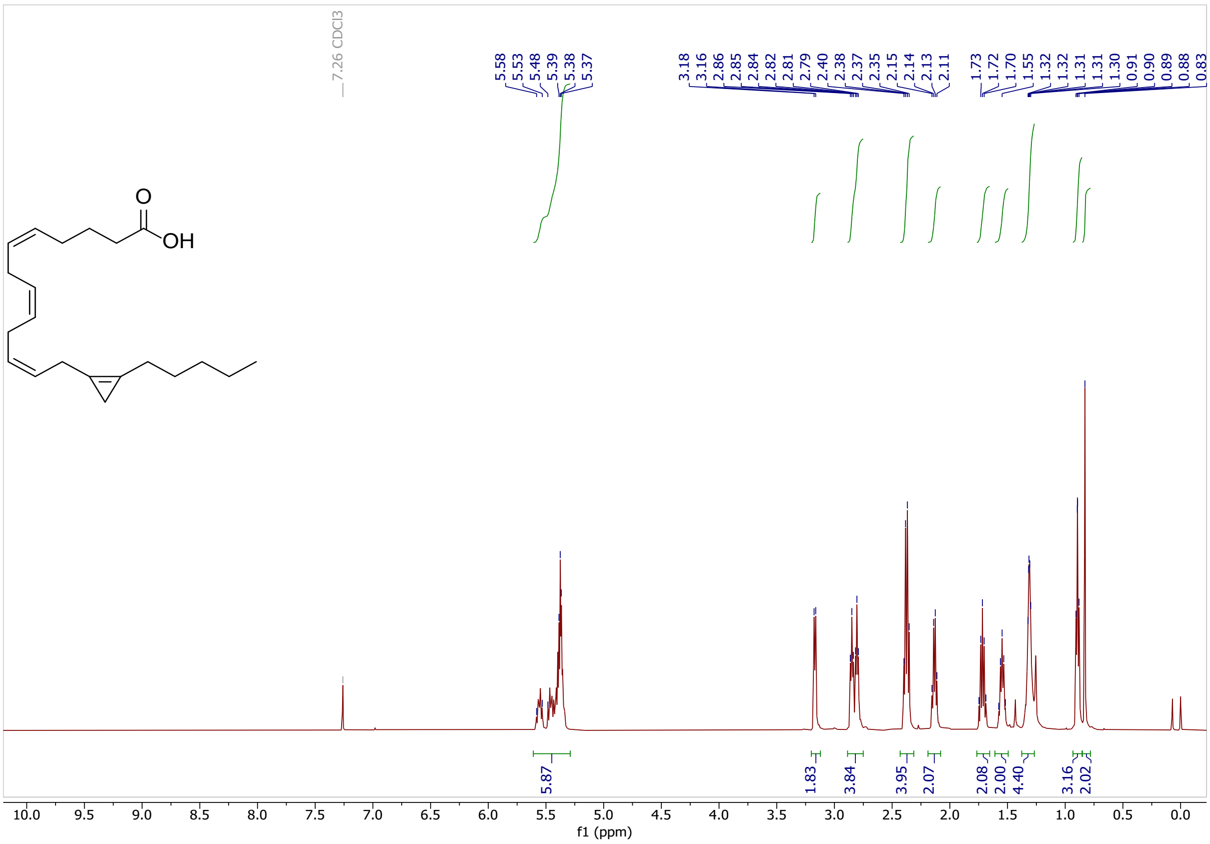


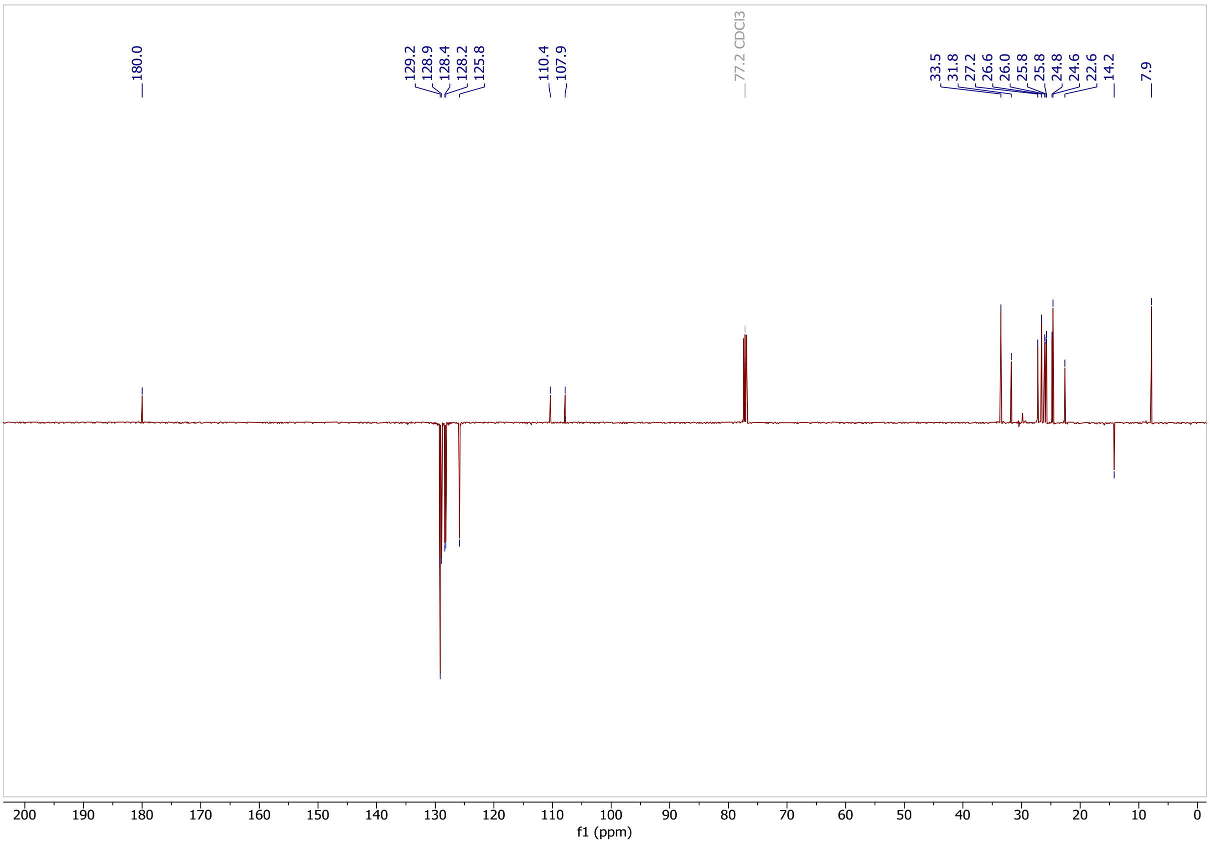

**8-(2-hexylcycloprop-1-en-1-yl)octanoic acid (3)**

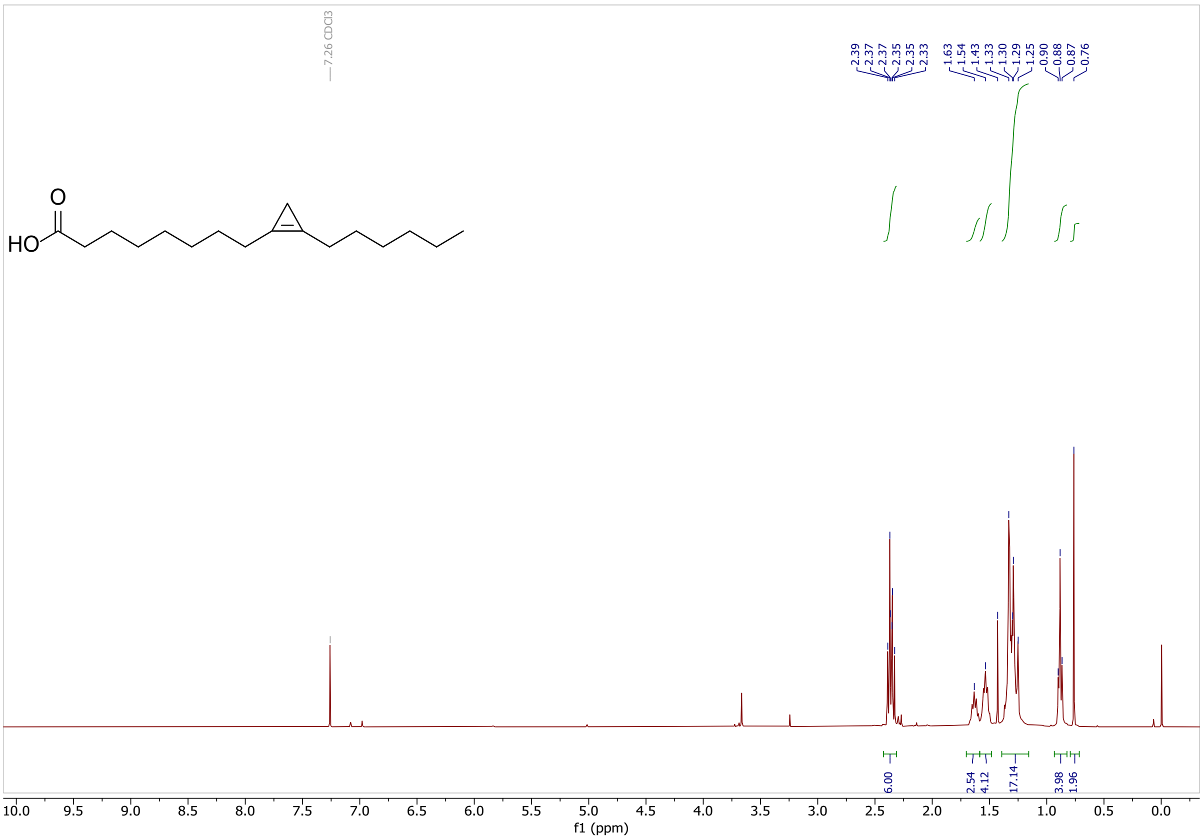


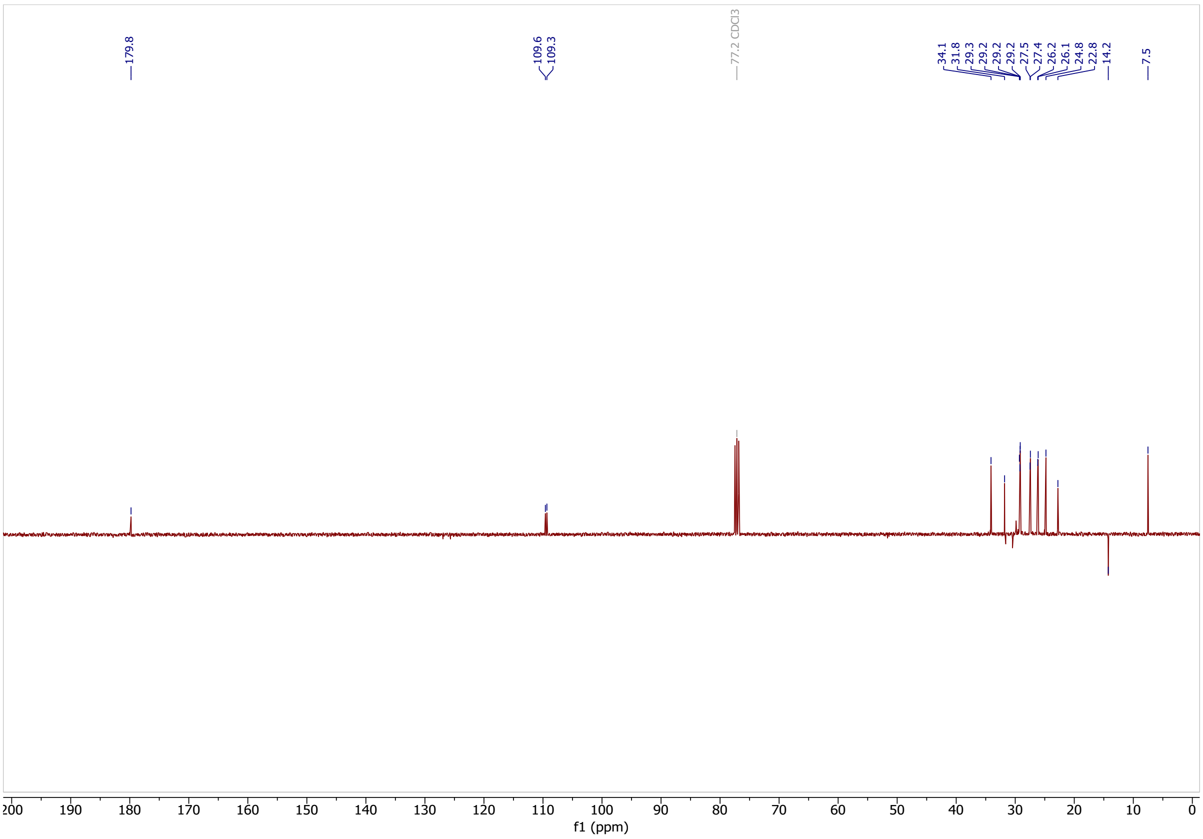

**1,1,2-tribromo-2-methylcyclopropane (25)**

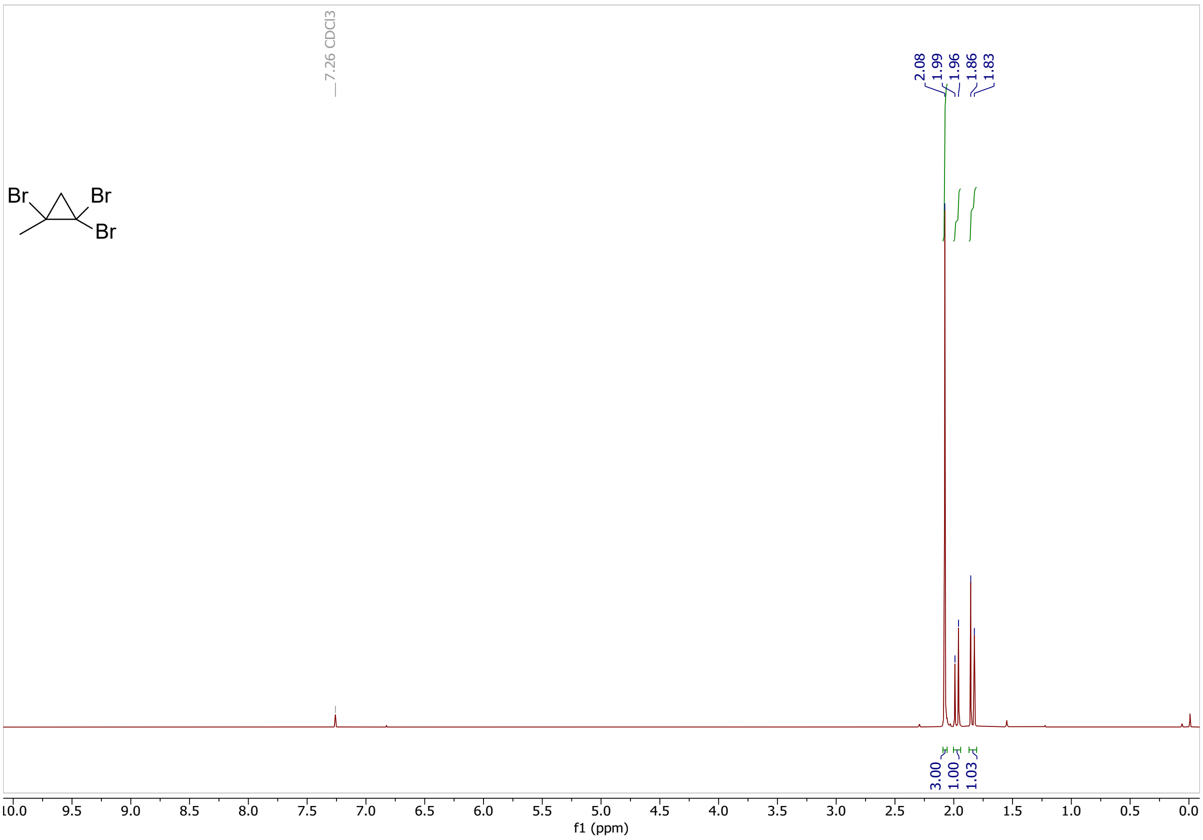


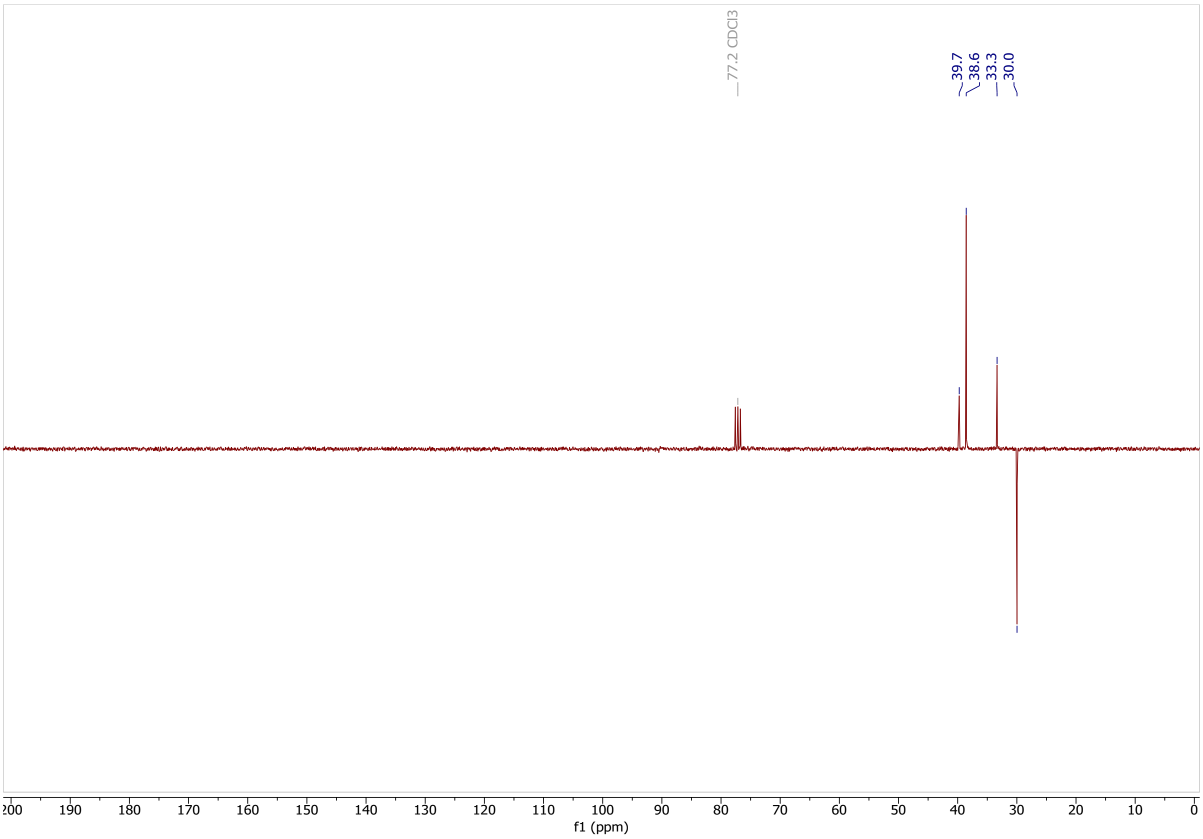

## 1-(12-iodododecyl)-2-methylcycloprop-1-ene (27)

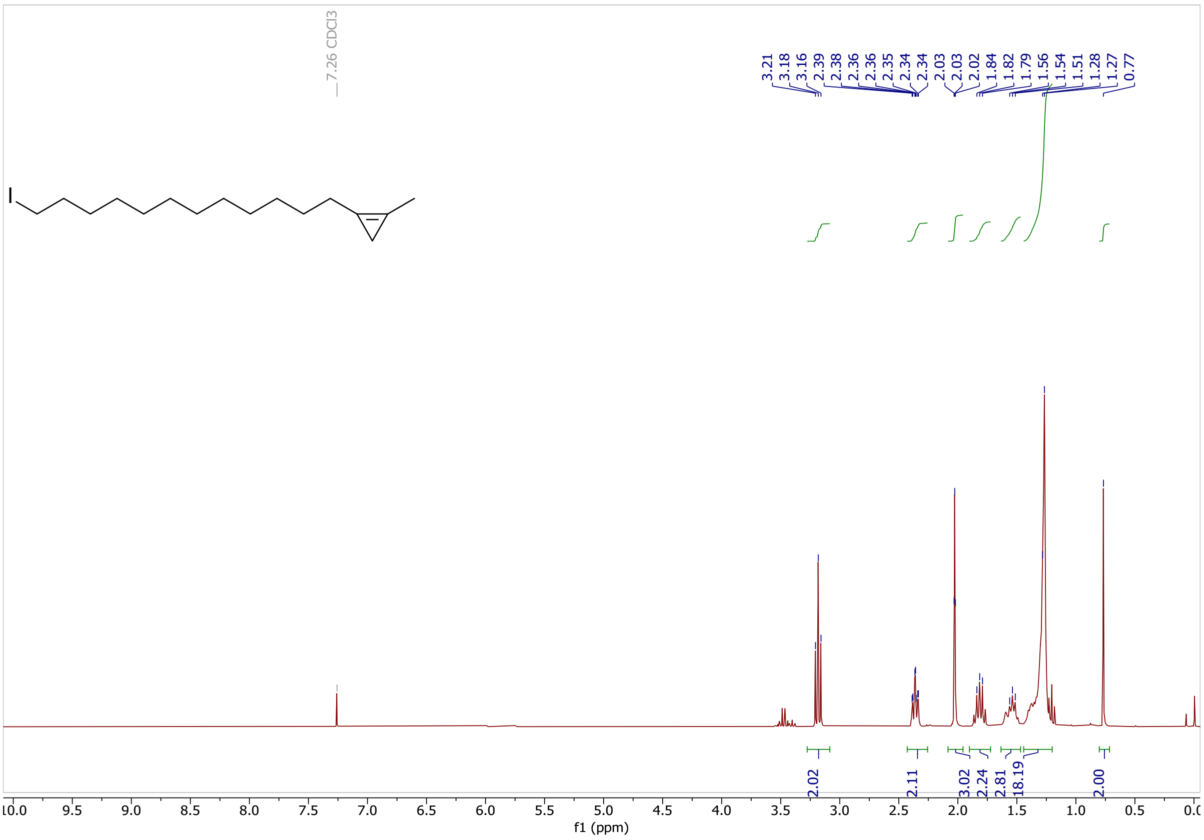


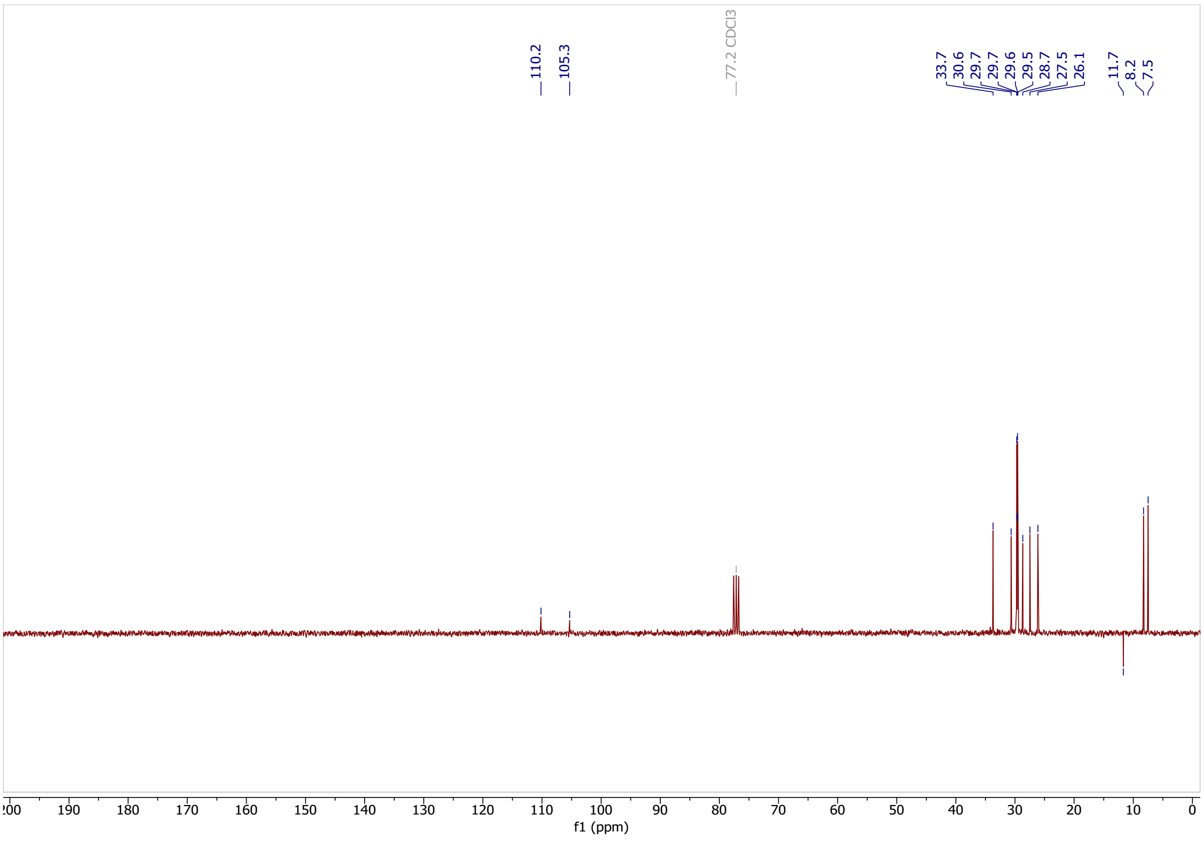

## 13-(2-methylcycloprop-1-en-1-yl)tridecanenitrile (30)

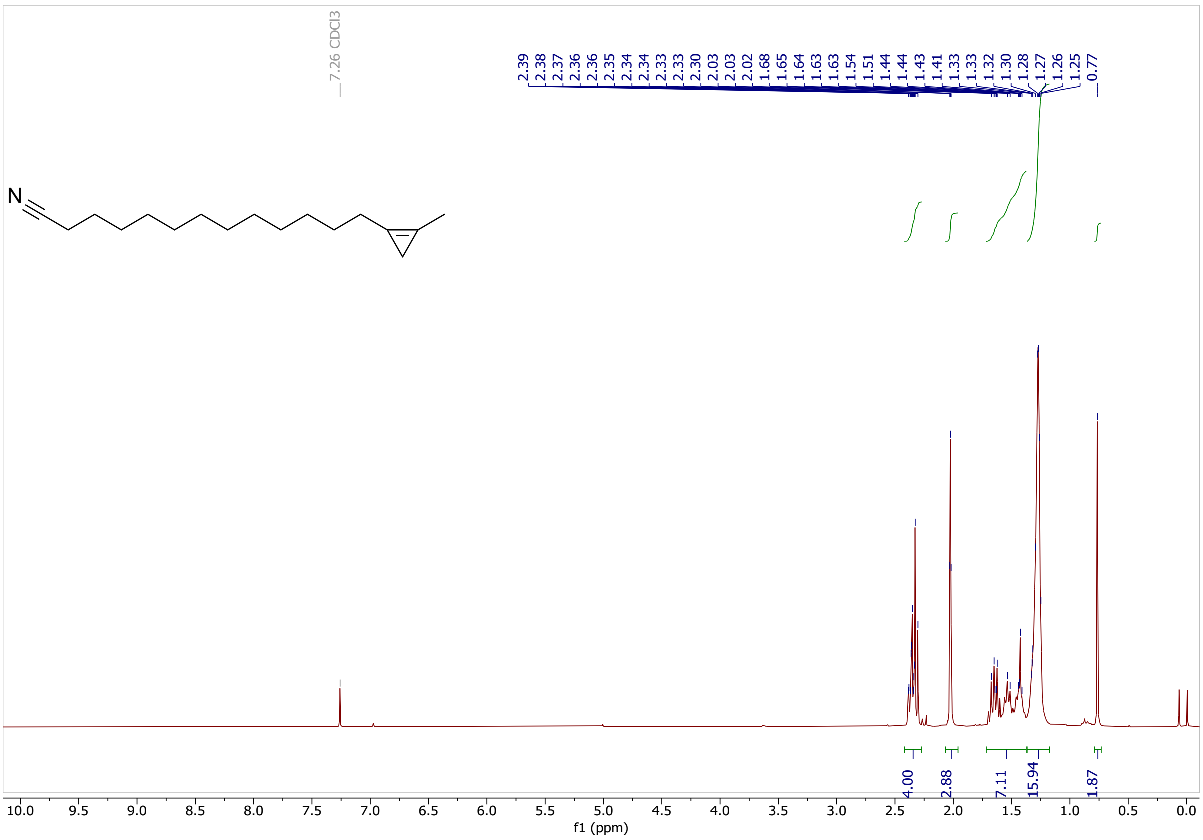


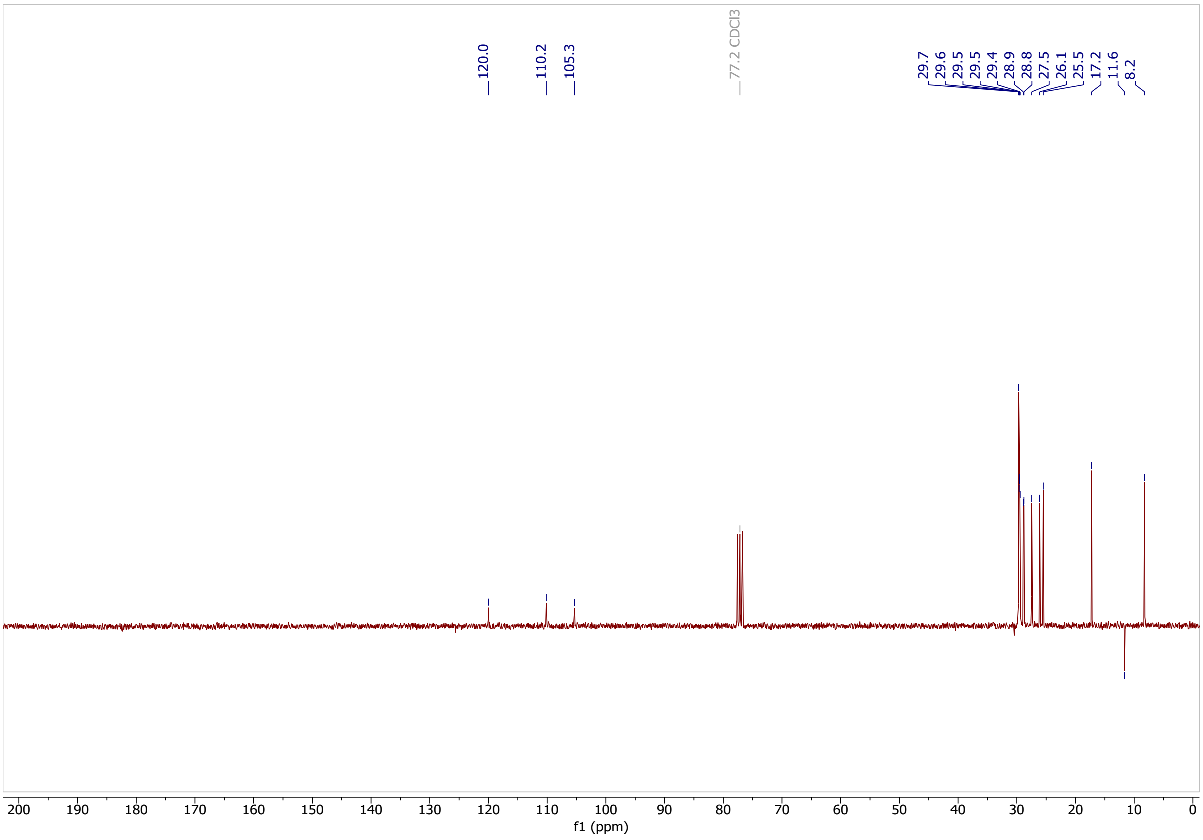

## 13-(2-methylcycloprop-1-en-1-yl)tridecanoic acid (1)

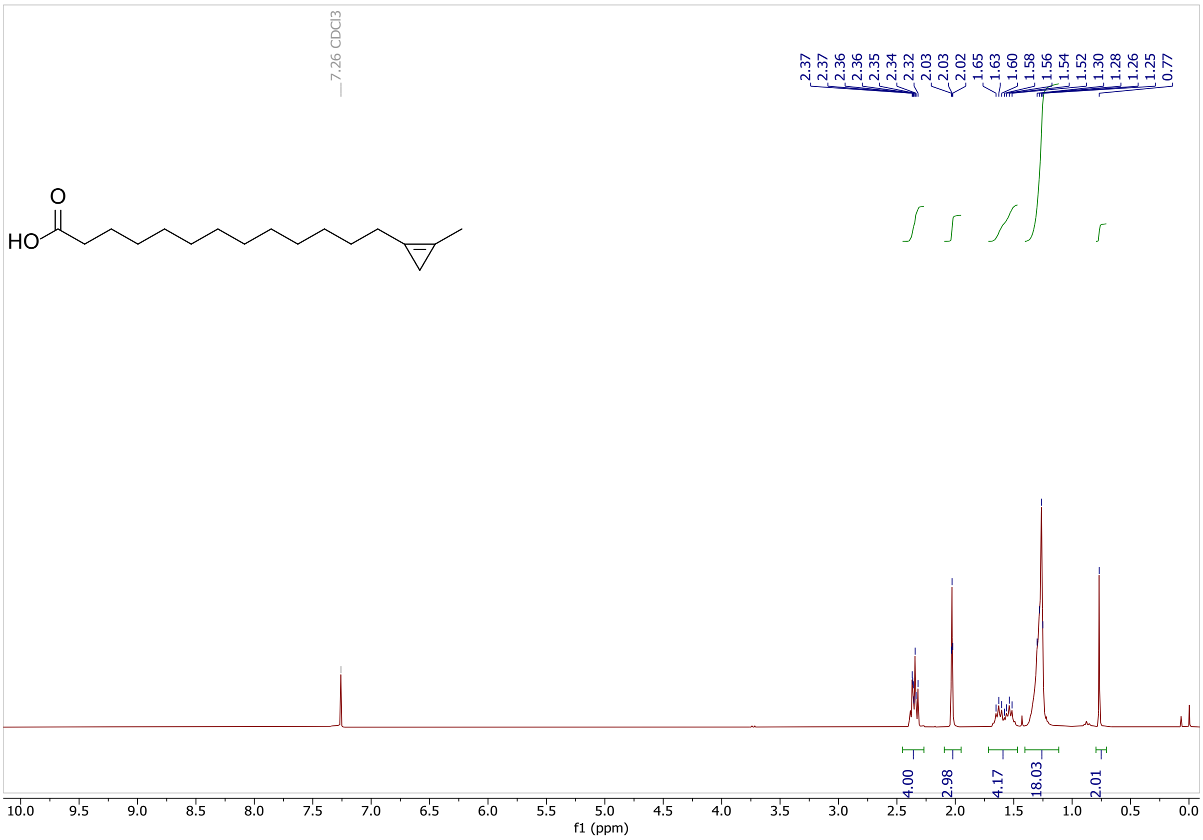


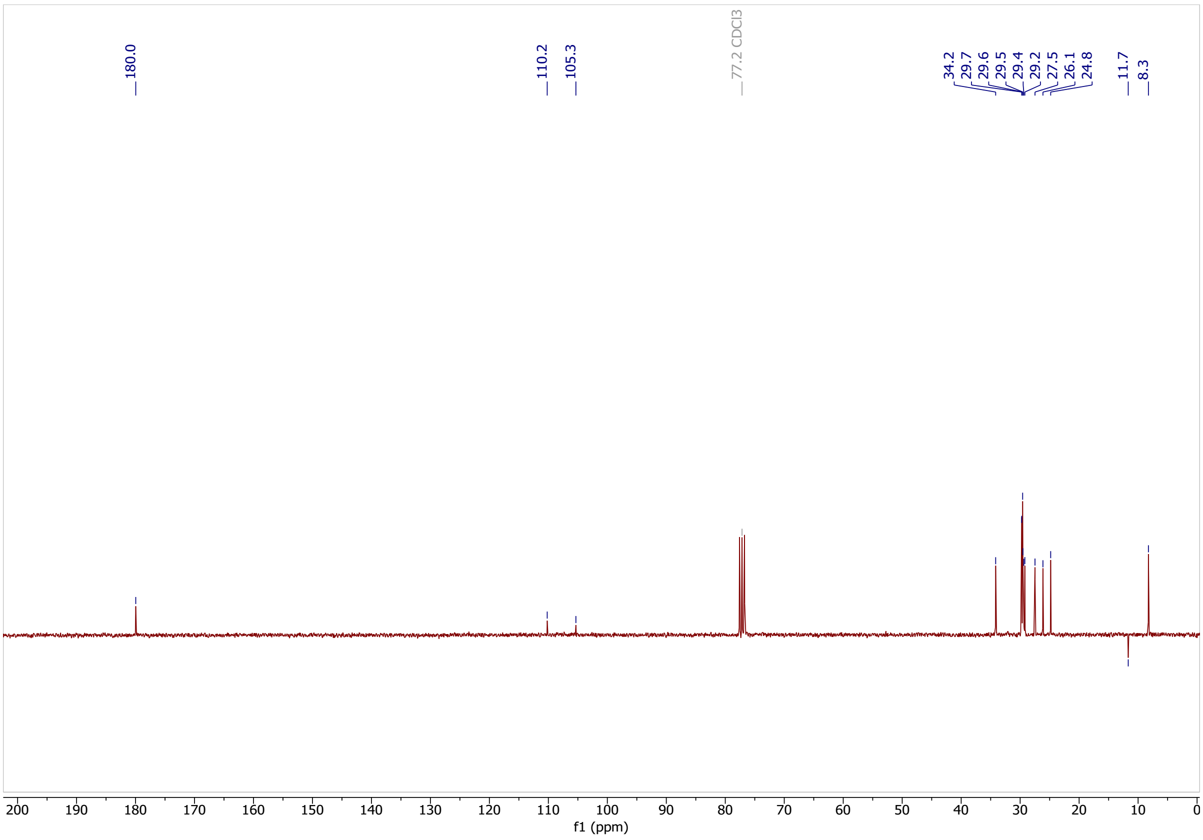

**1-(14-iodotetradecyl)-2-methylcycloprop-1-ene (28)**

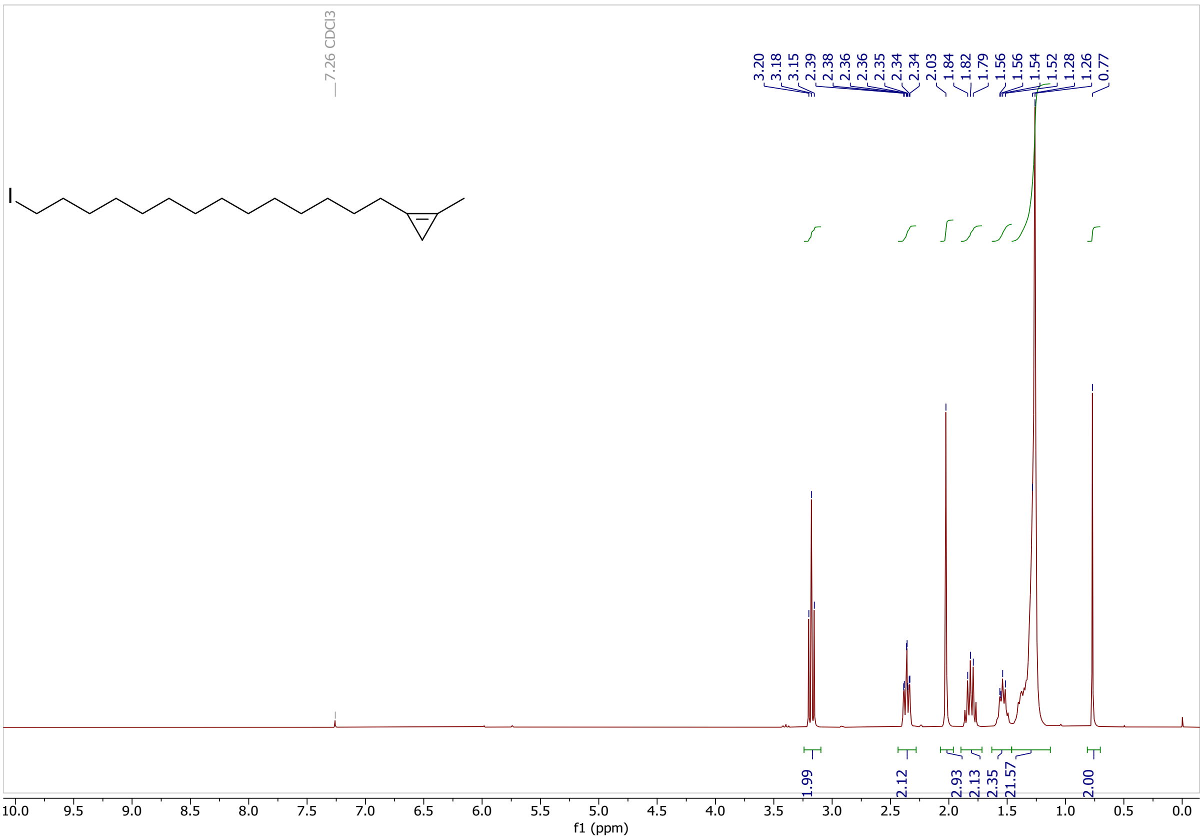


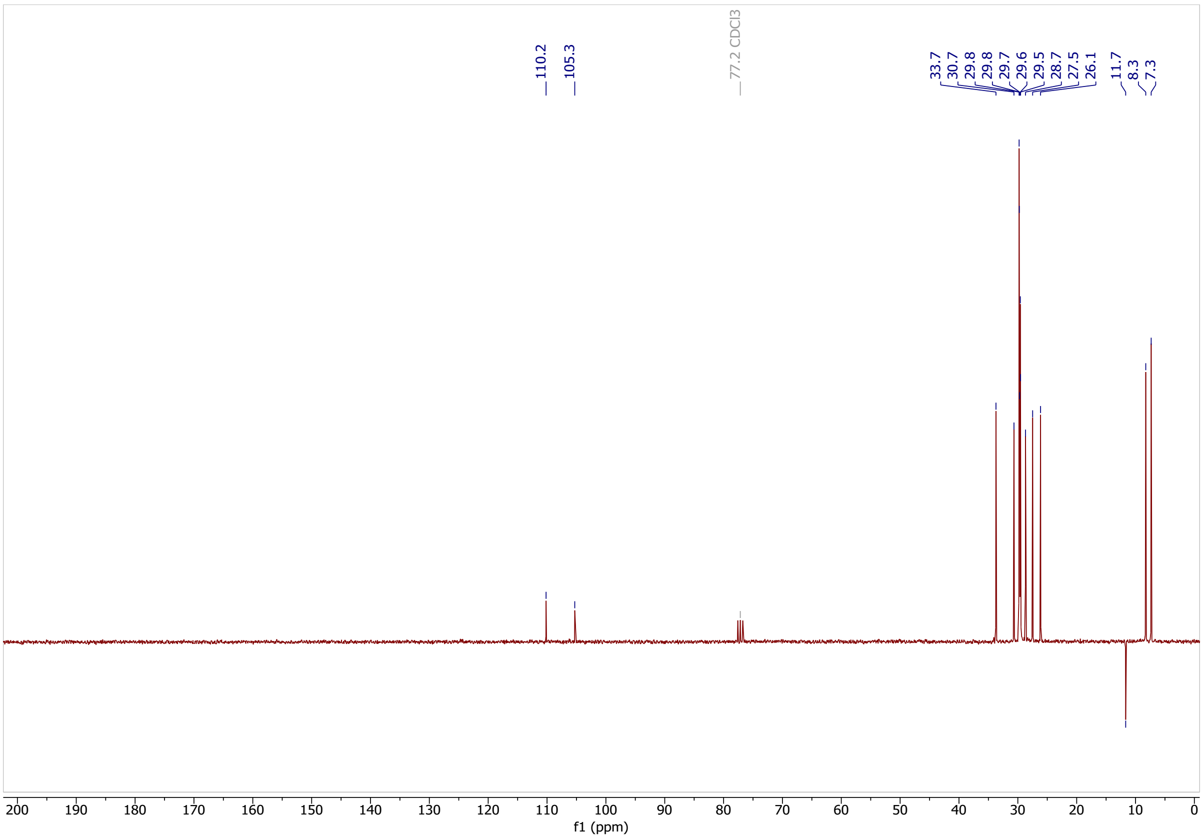

## 15-(2-methylcycloprop-1-en-1-yl)pentadecanenitrile (31)

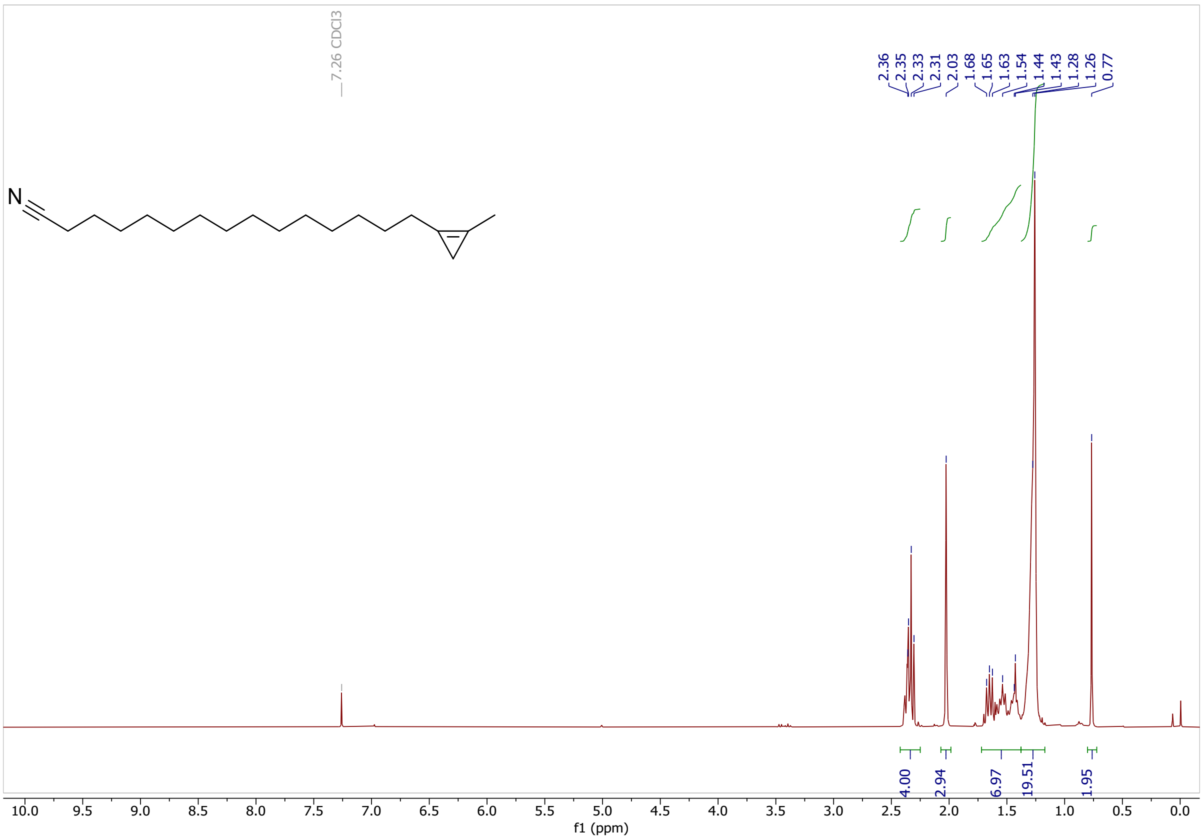


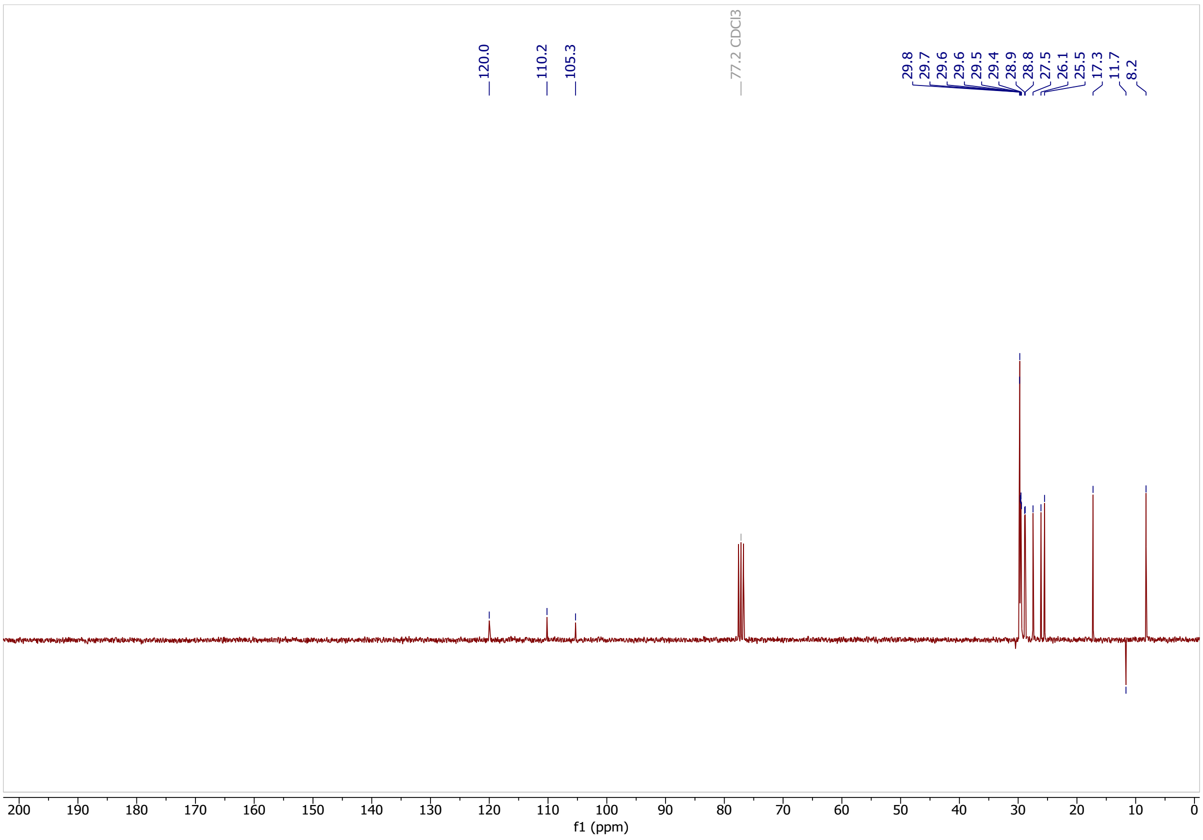

## 15-(2-methylcycloprop-1-en-1-yl)pentadecanoic acid (2)

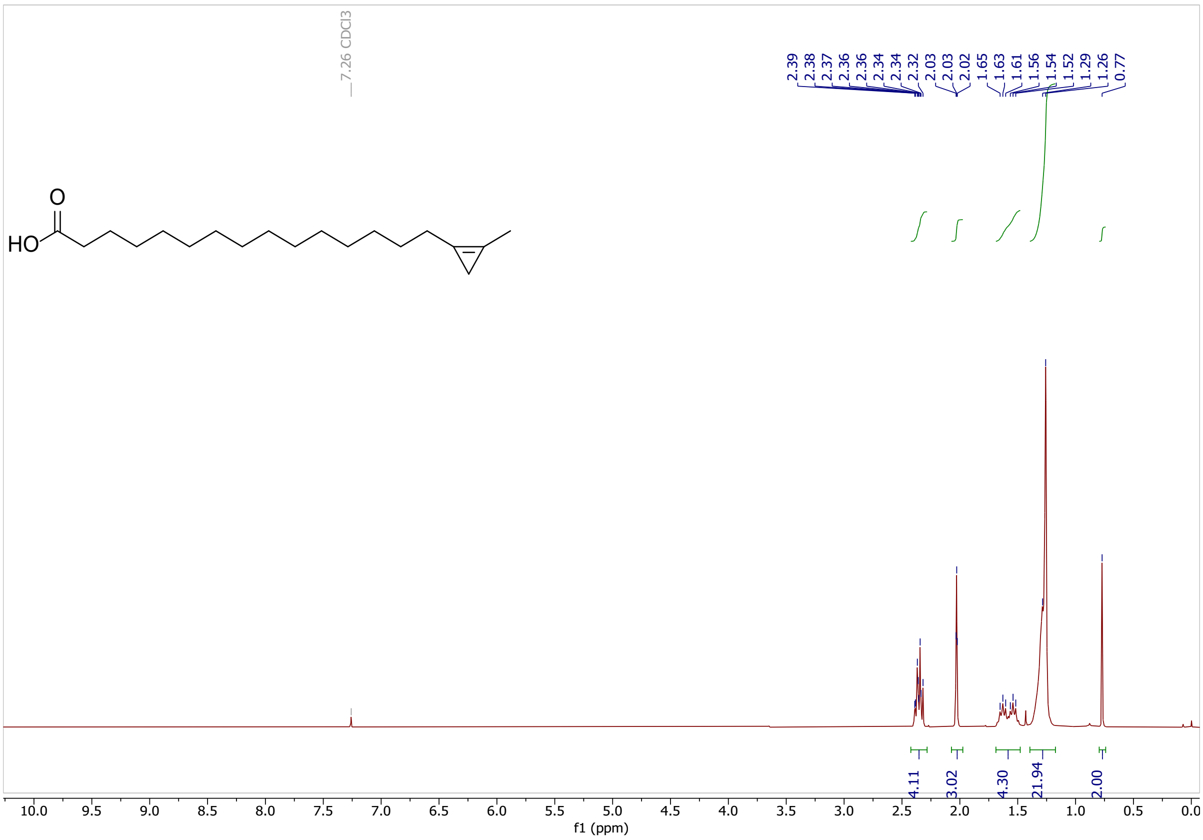


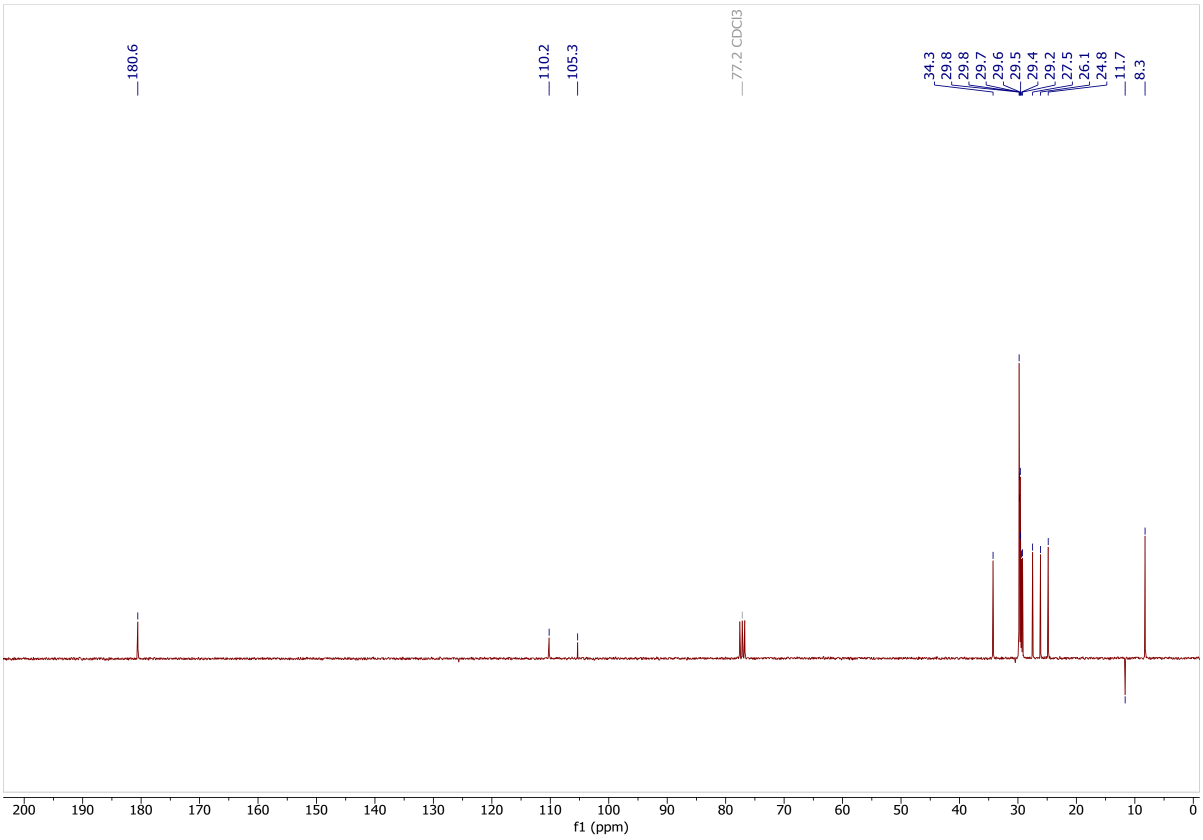

**2-bromodec-1-ene (24)**

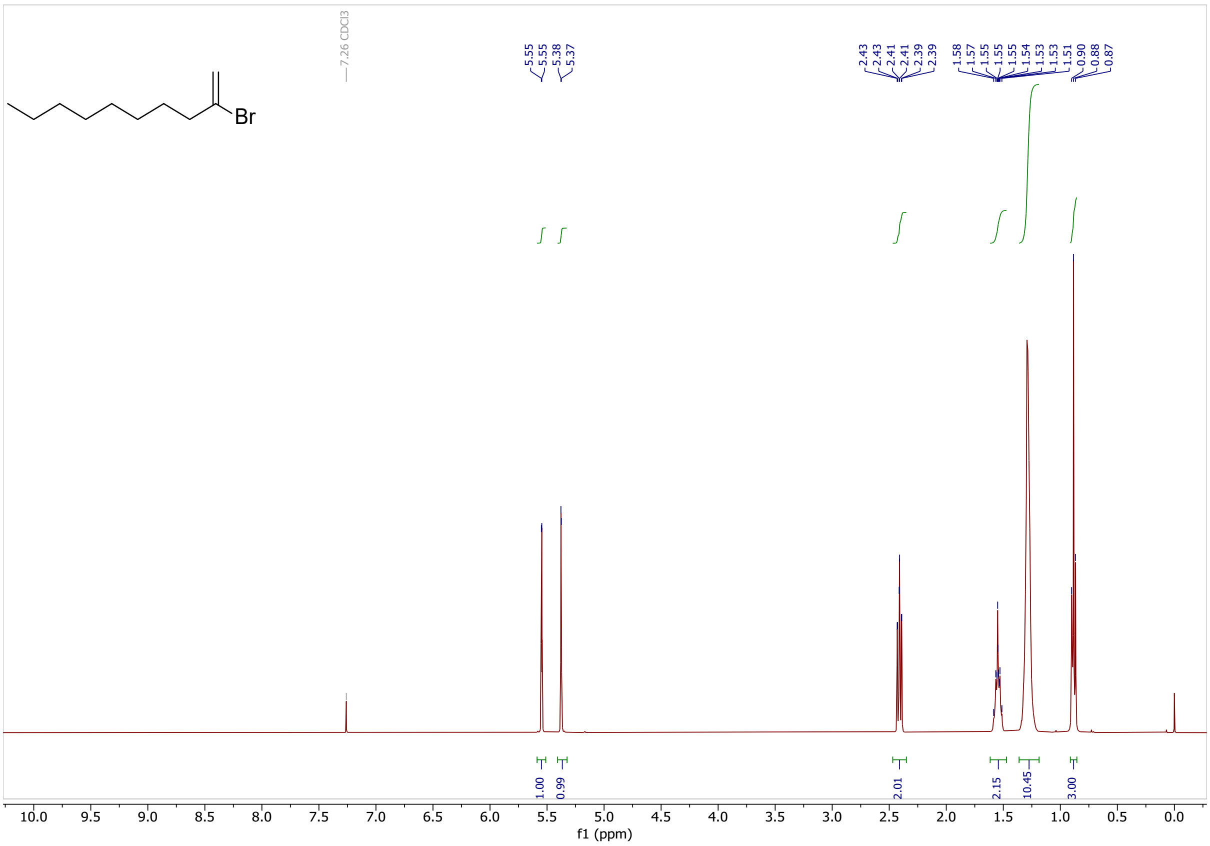


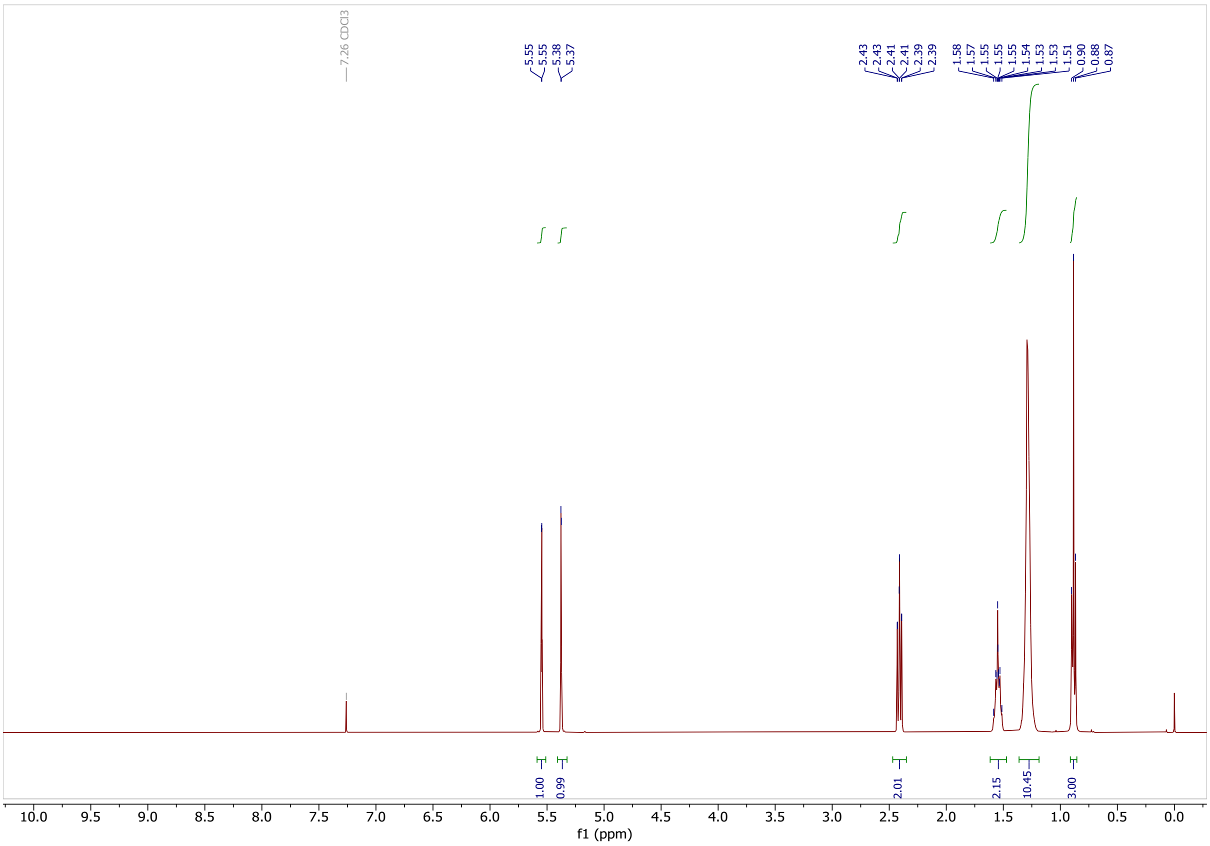

## 1,1,2-tribromo-2-octylcyclopropane (26)

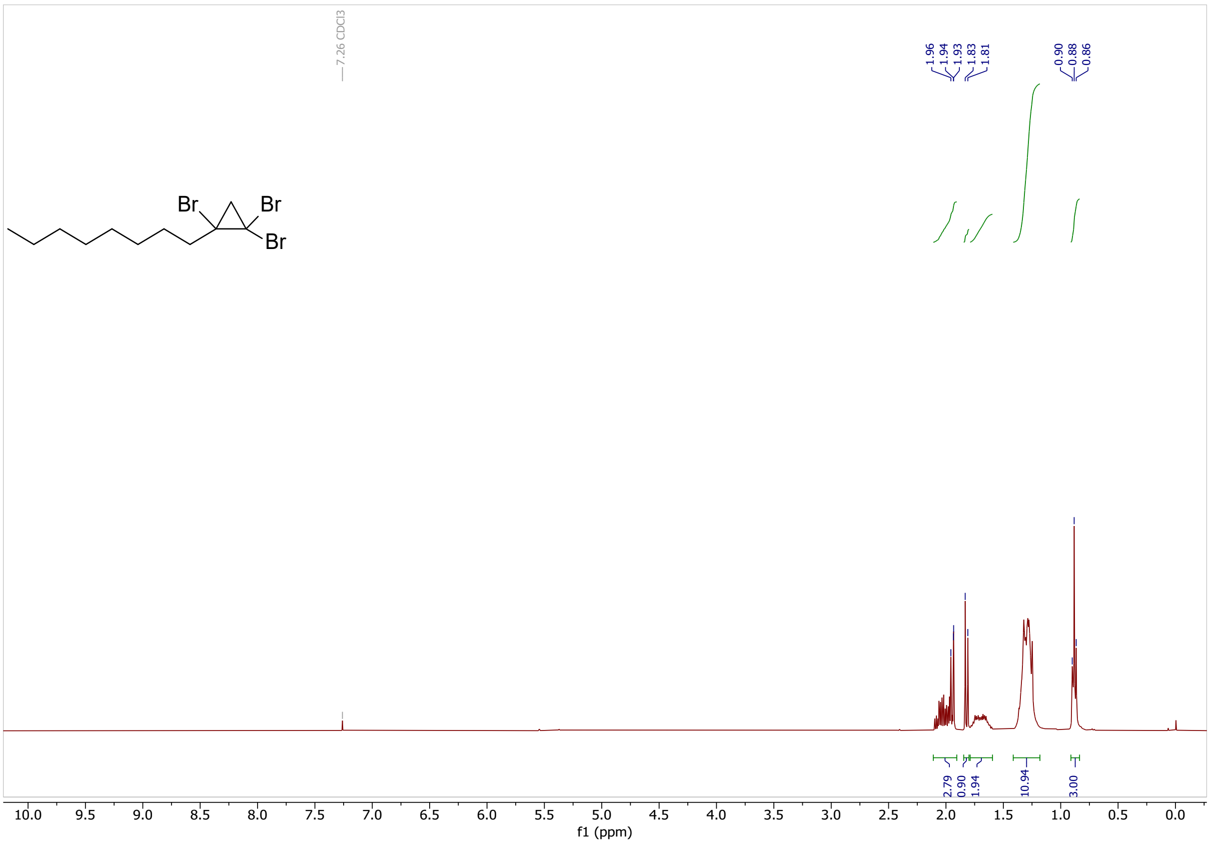


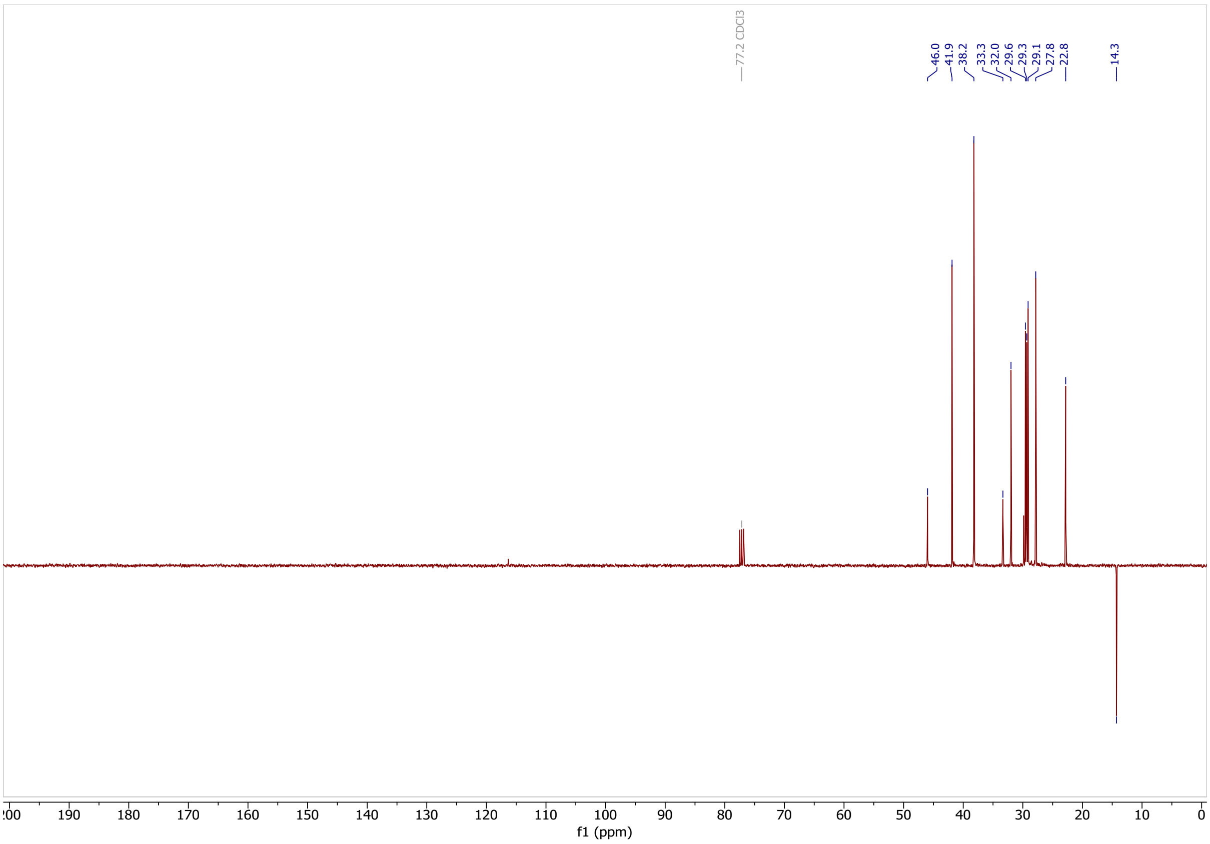

## 1-(7-iodoheptyl)-2-octylcycloprop-1-ene (29)

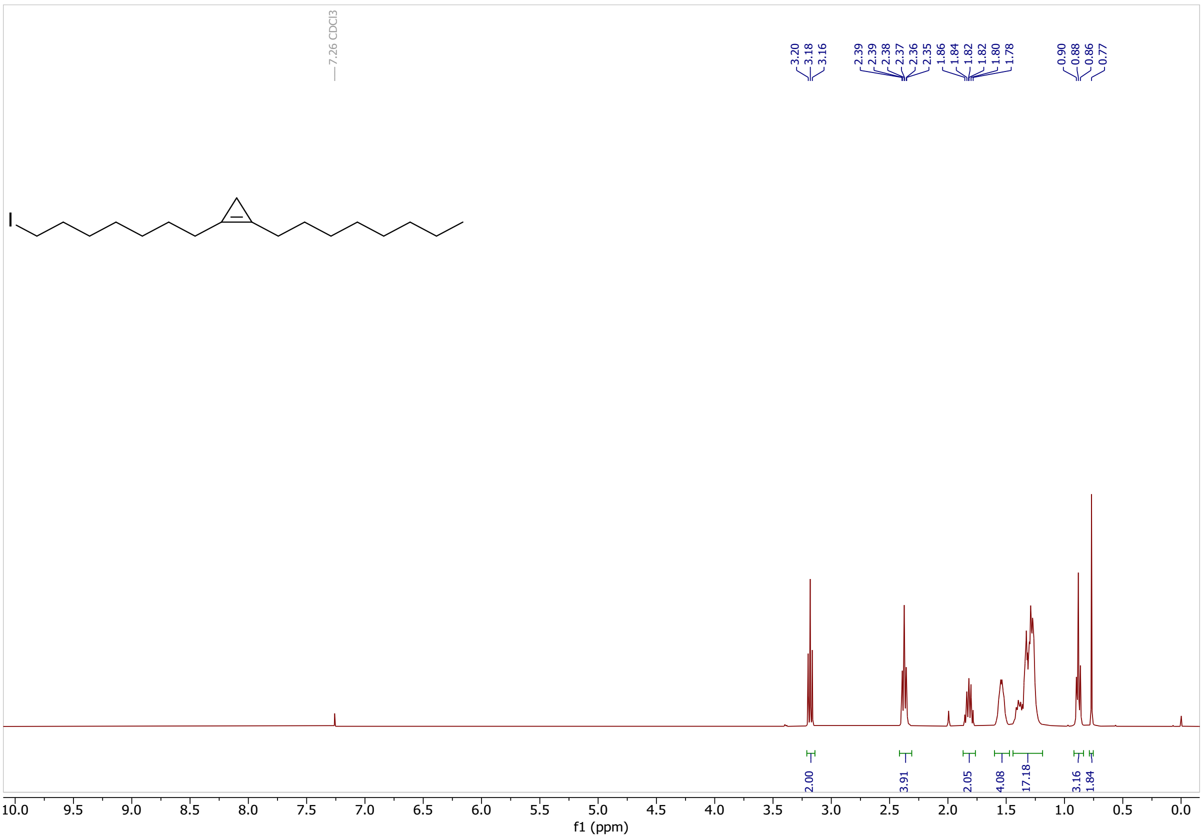


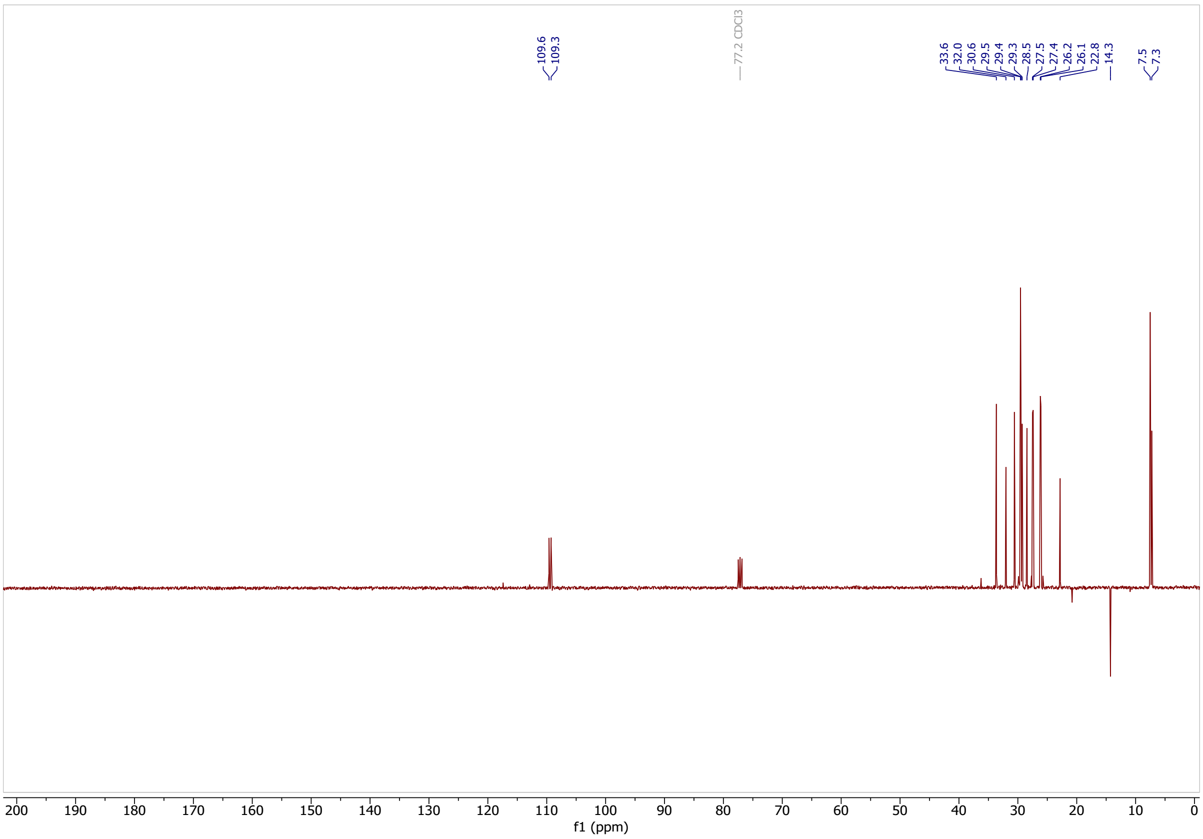

**8-(2-octylcycloprop-1-en-1-yl)octanenitrile (32)**

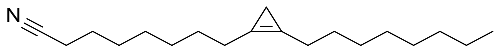


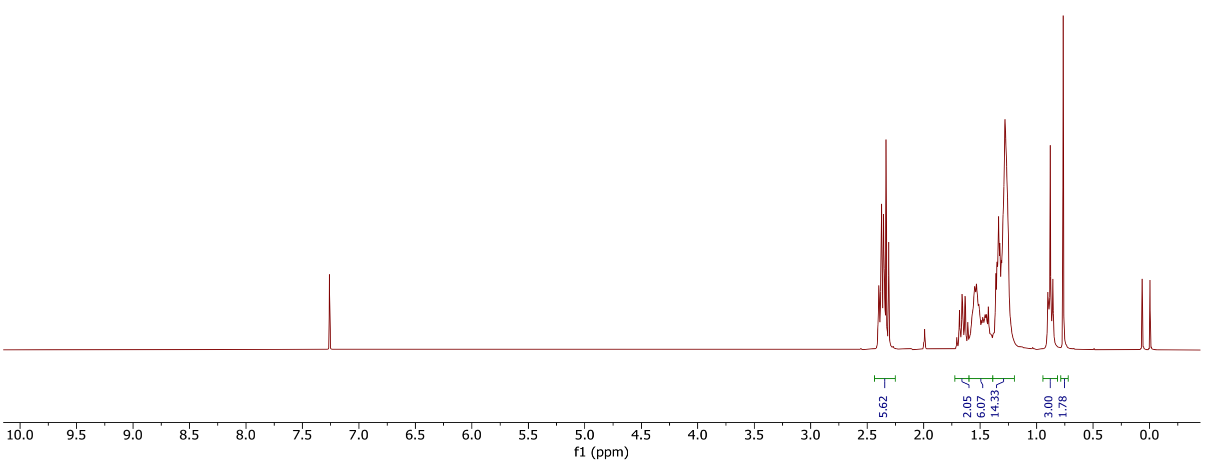


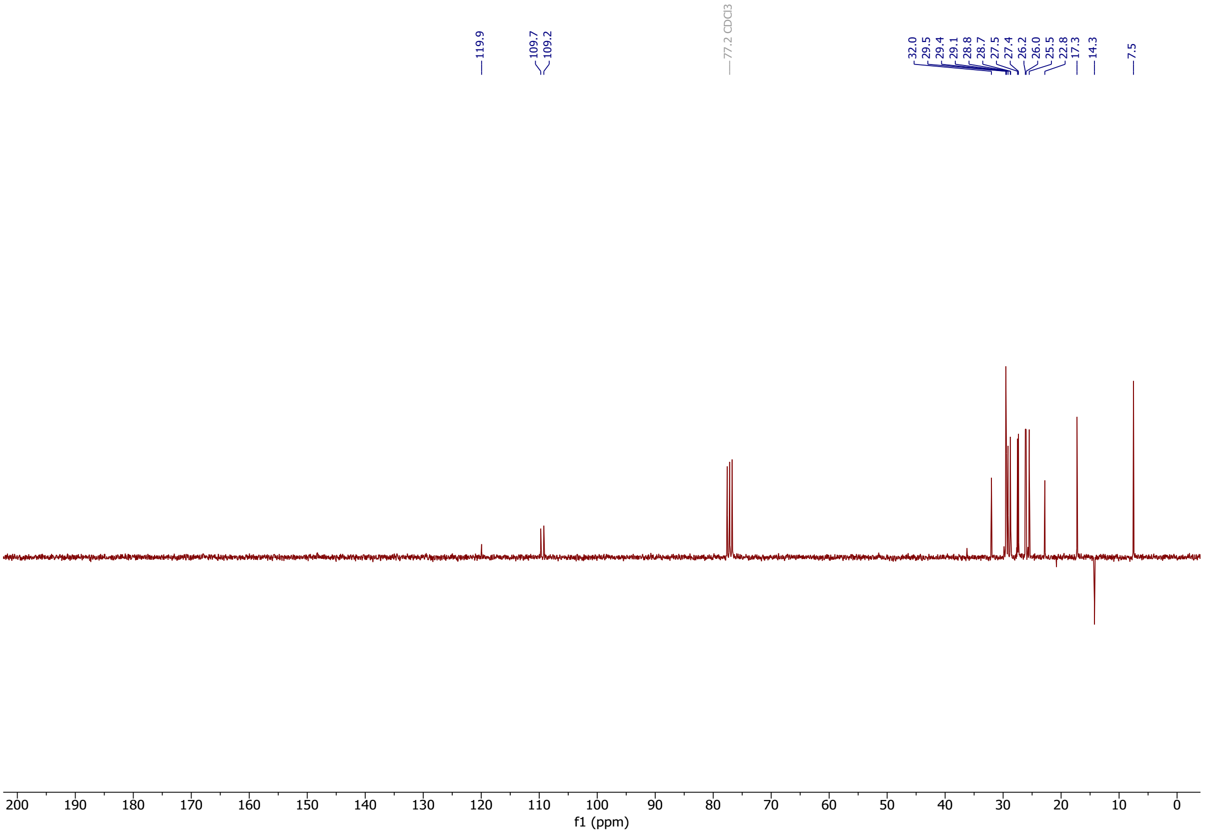

**8-(2-octylcycloprop-1-en-1-yl)octanoic acid (4)**

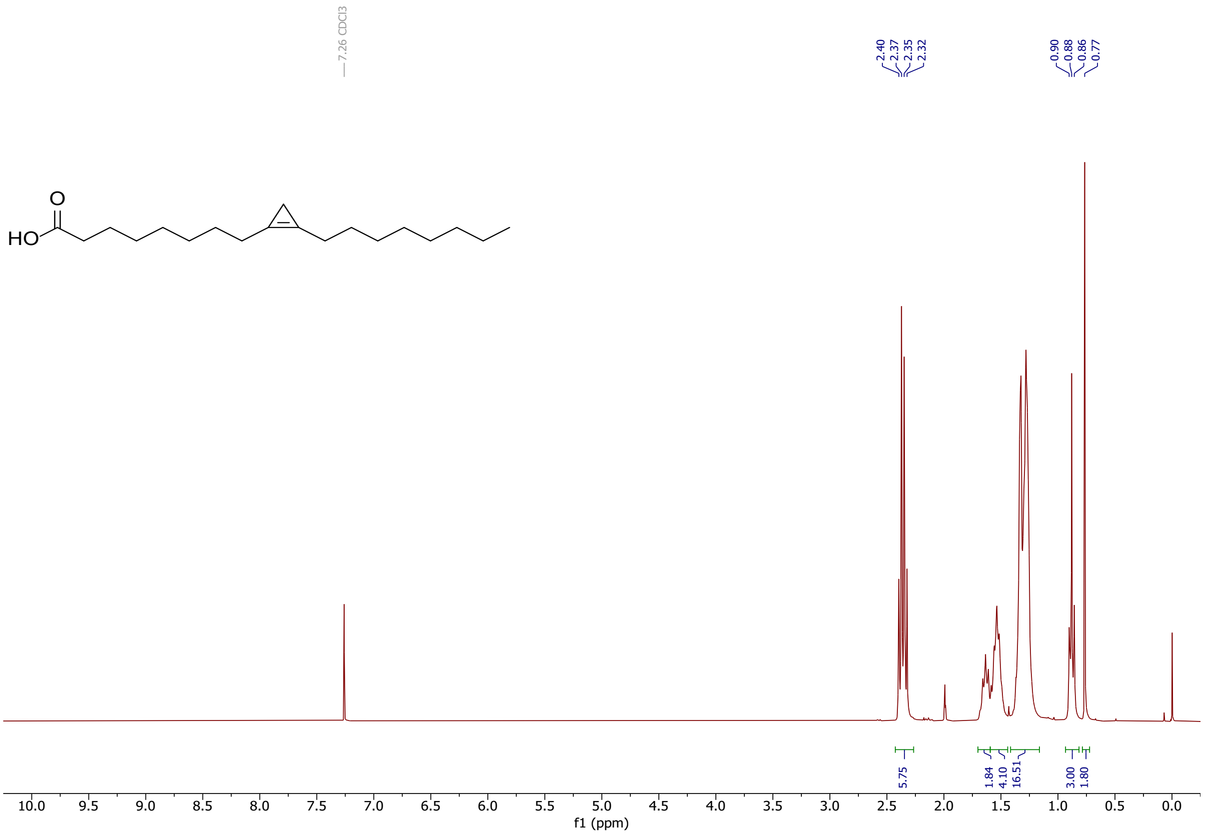

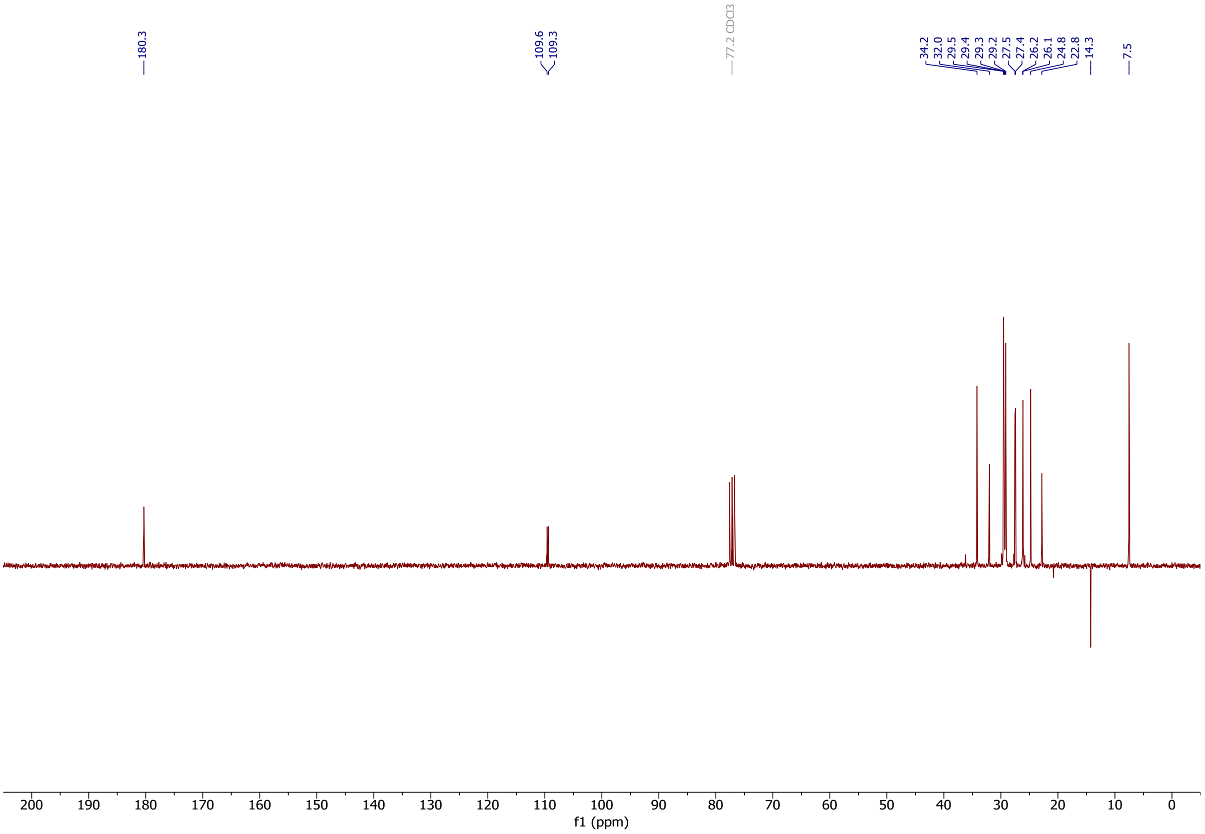

## 9-bromononanoic acid (s4)

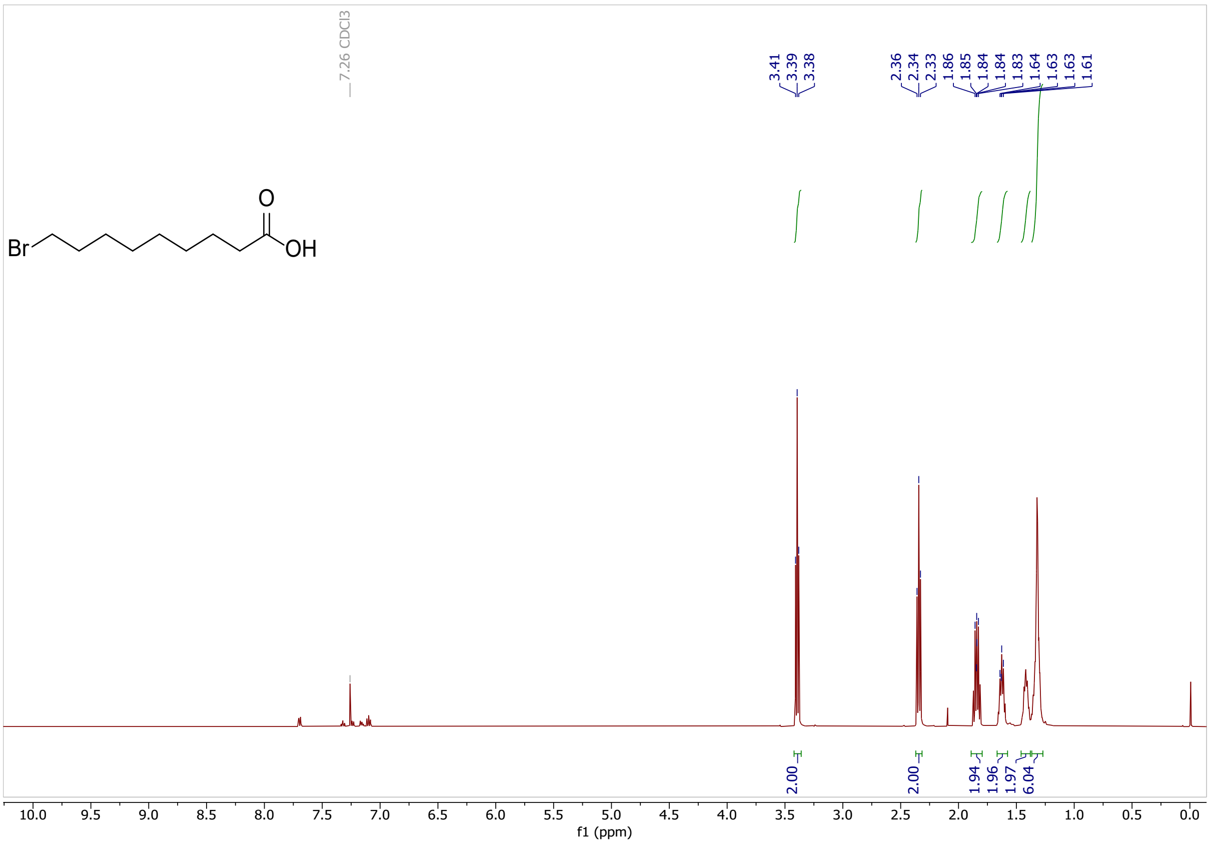


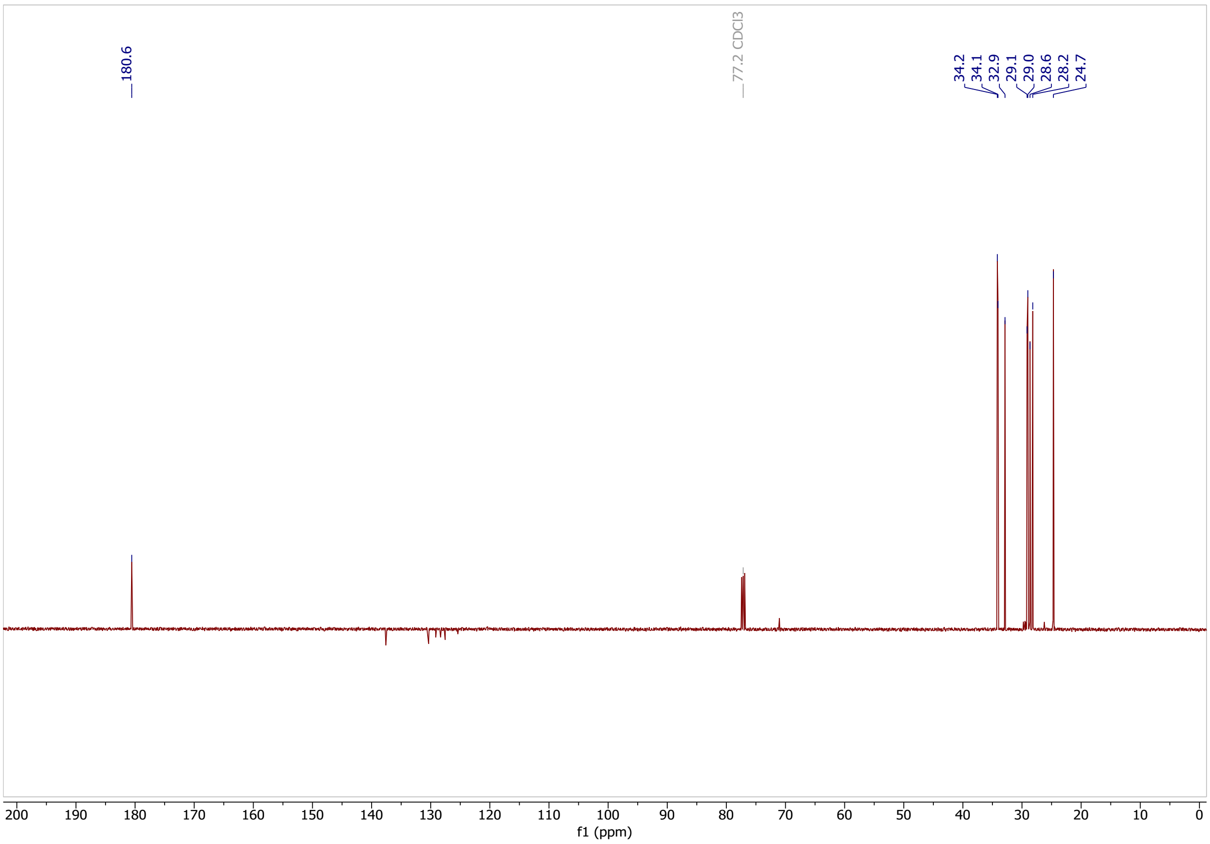

**8-Carboxyoctyl)triphenylphosphonium bromide (s5)**

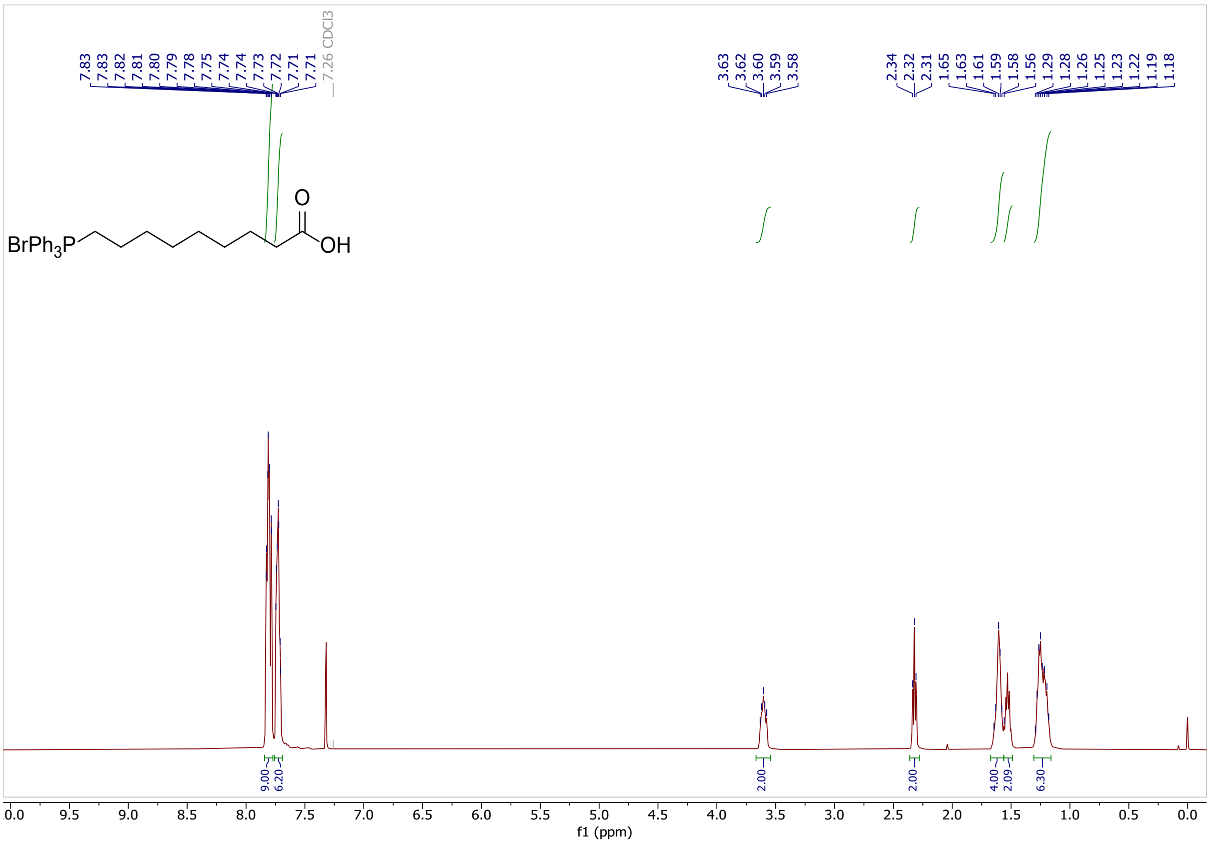


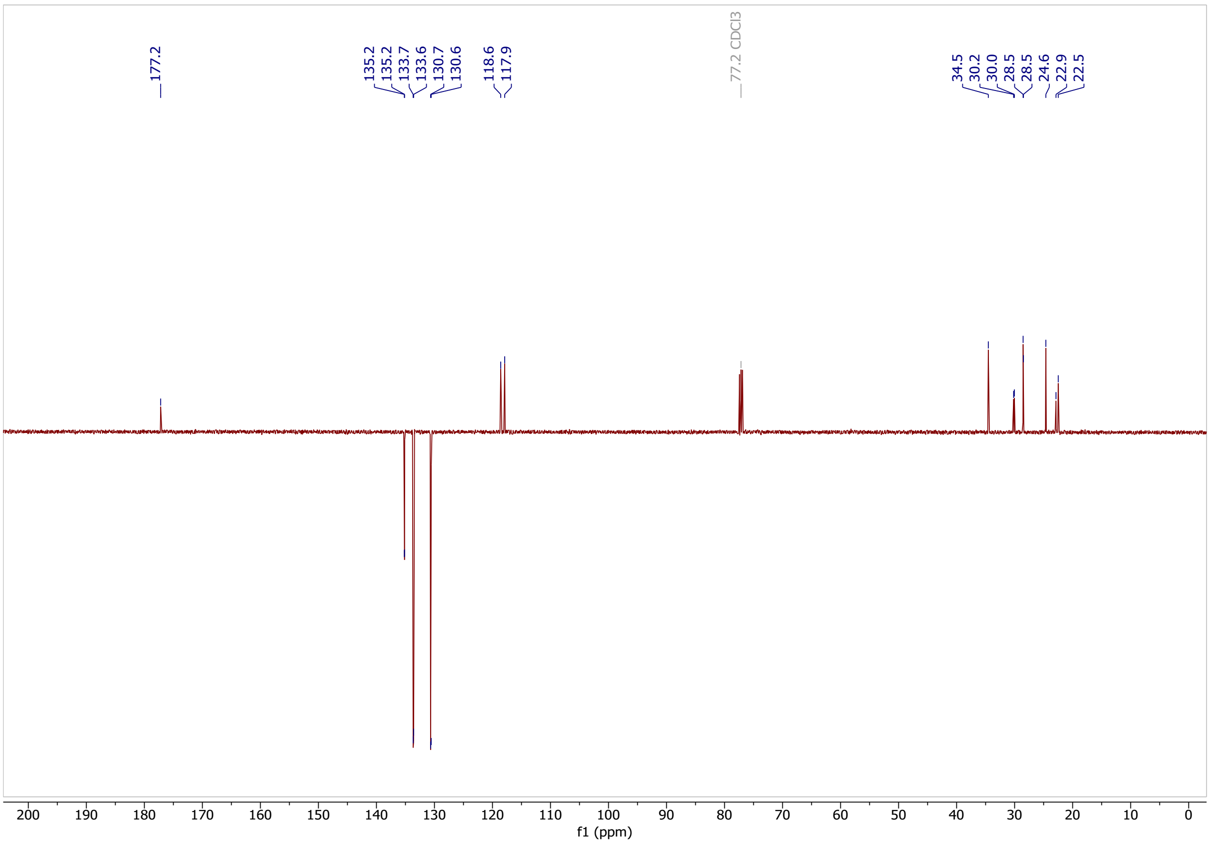

## (2-(1,3-dioxan-2-yl)ethyl)triphenylphosphonium bromide (s6)

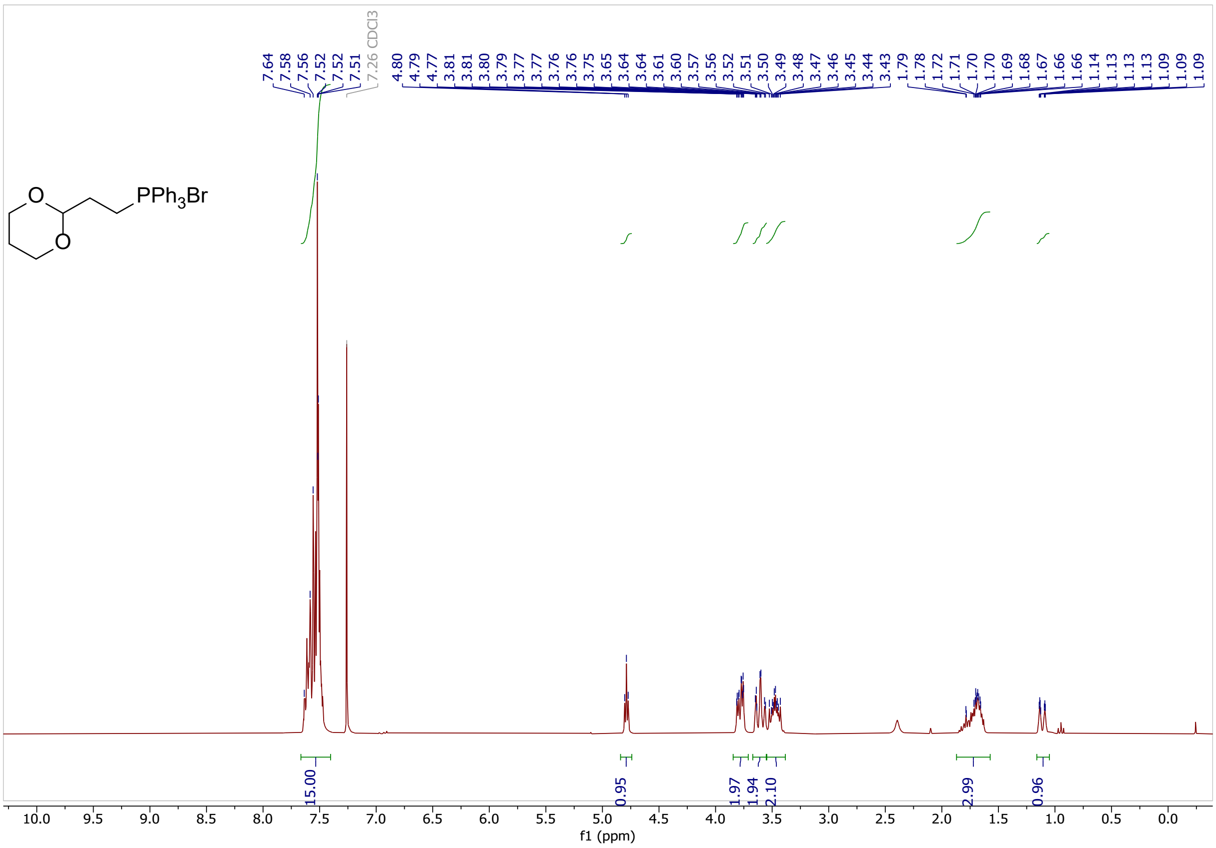


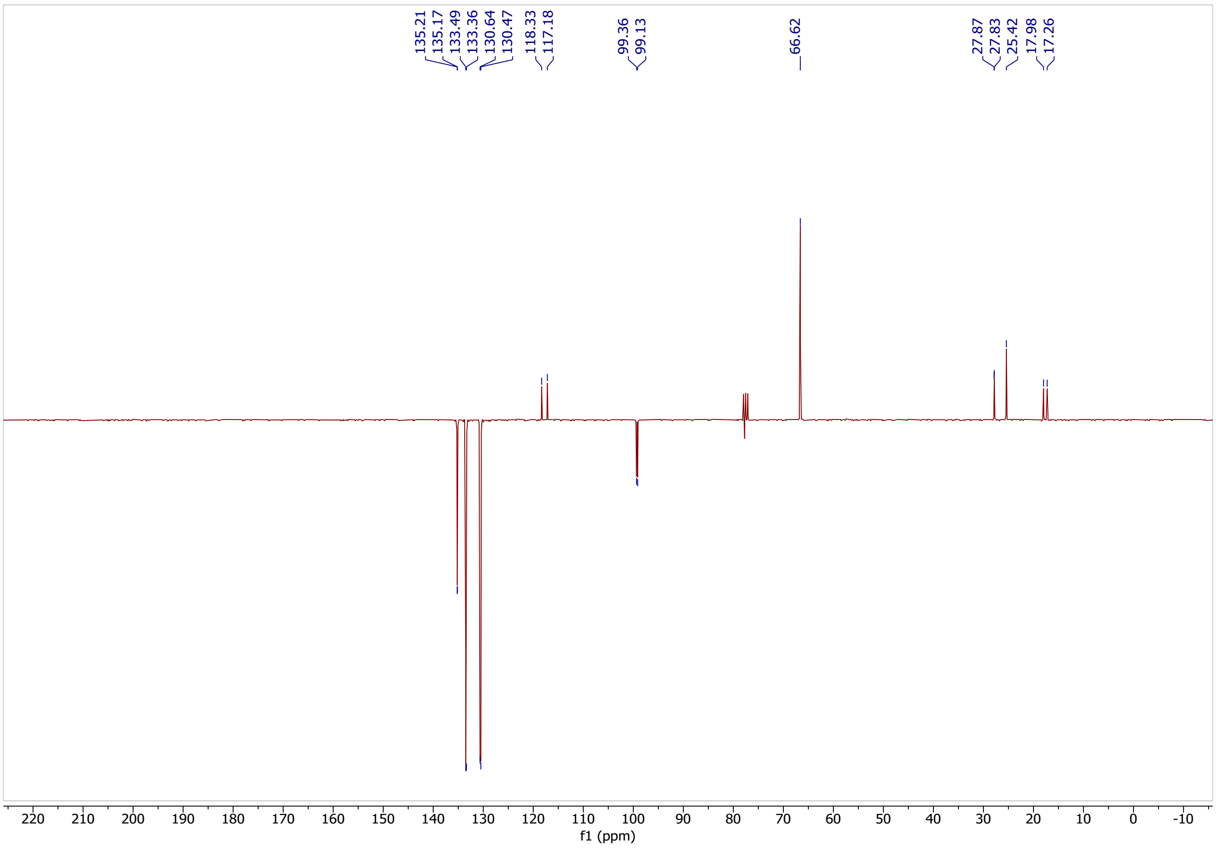

## methyl 5-oxopentanoate (s7)

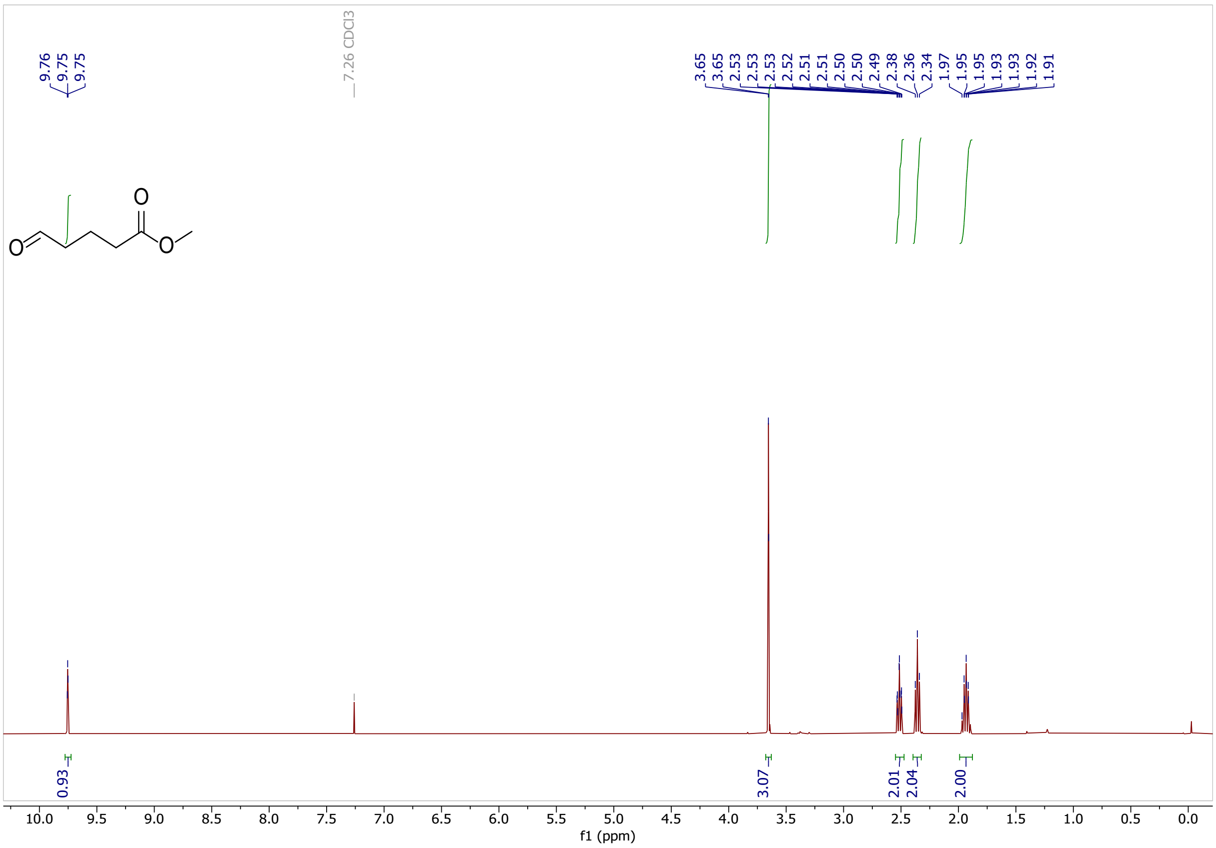


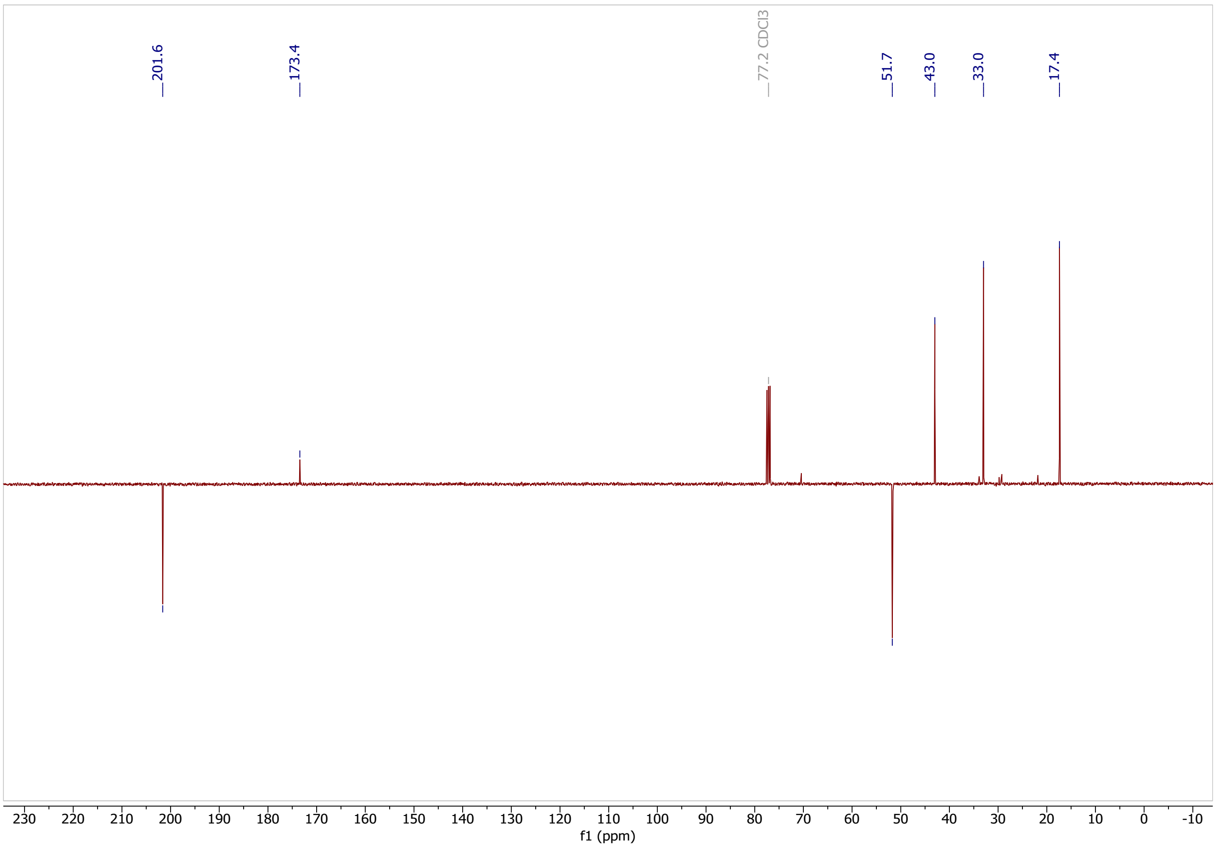

**methyl (Z)-7-(1,3-dioxan-2-yl)hept-5-enoate (s8)**

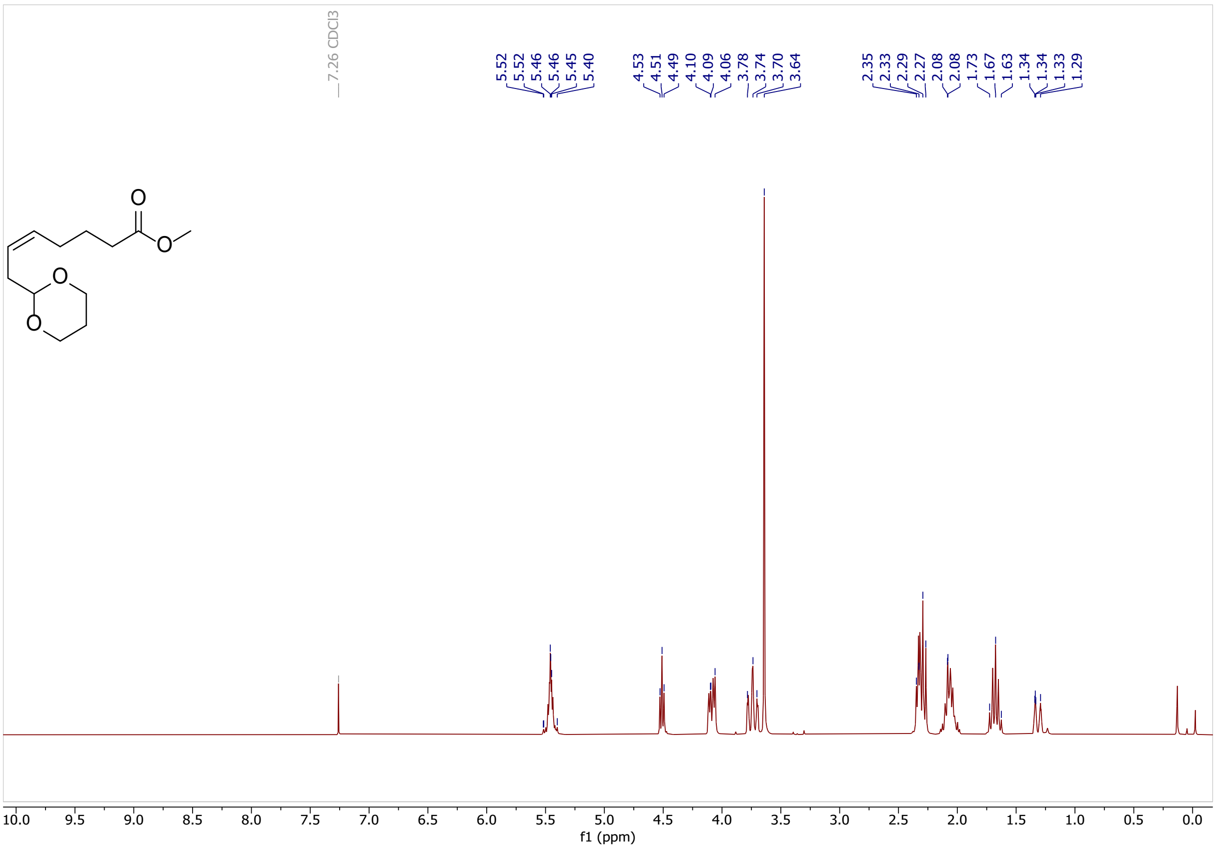


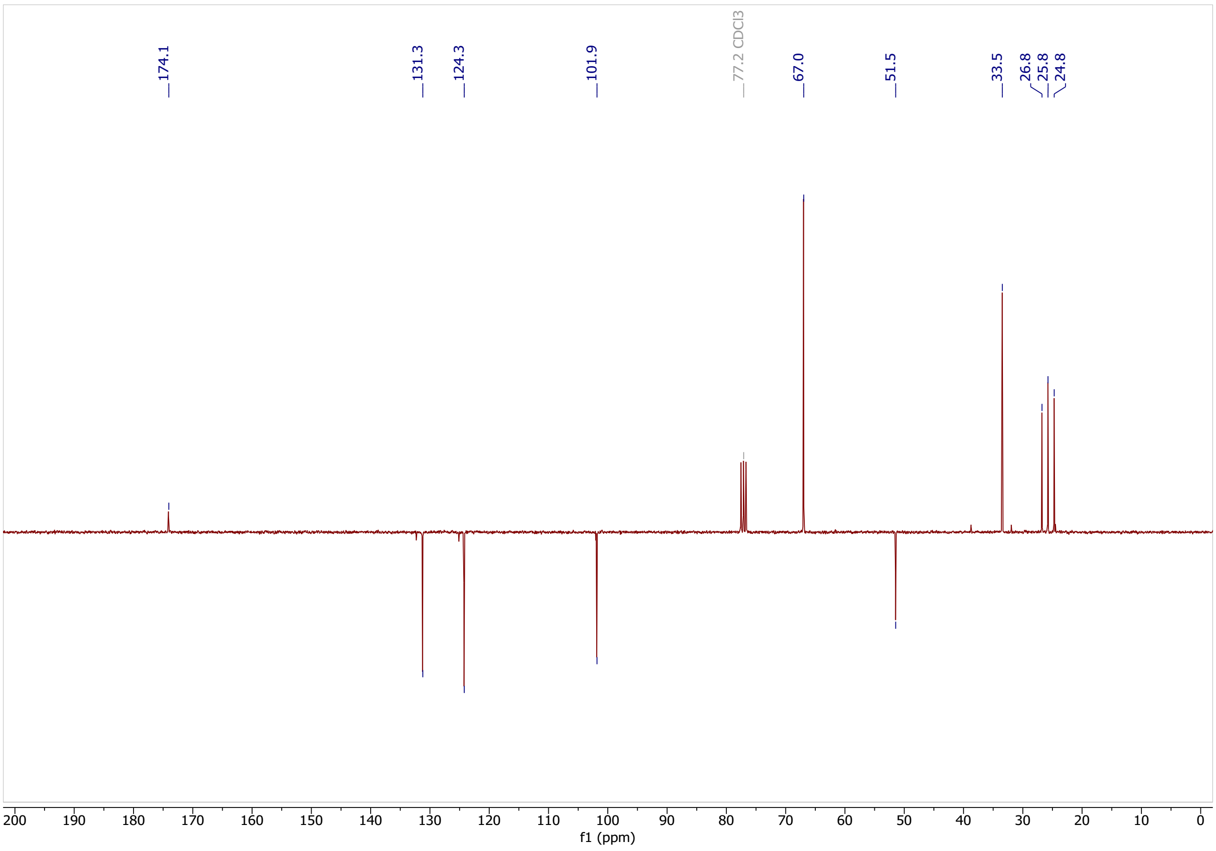

## hexadec-9-yn-1-ol (S10)

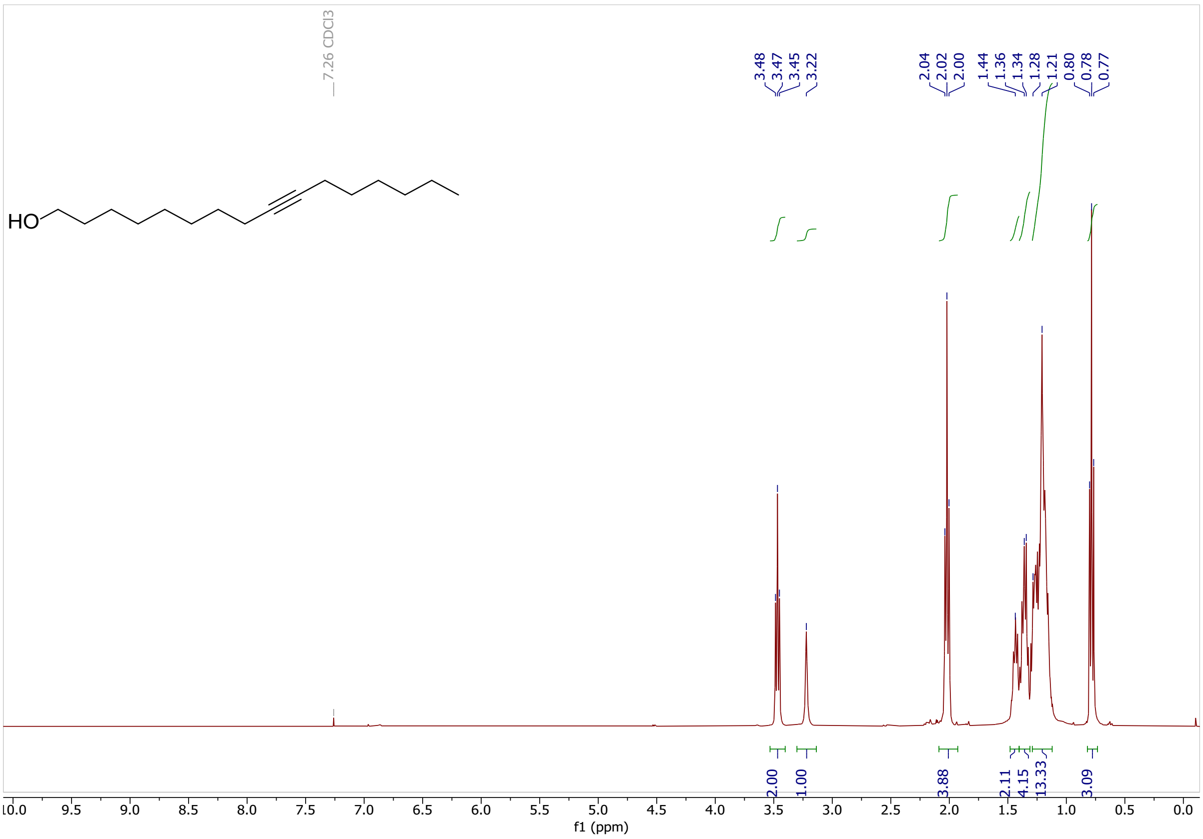


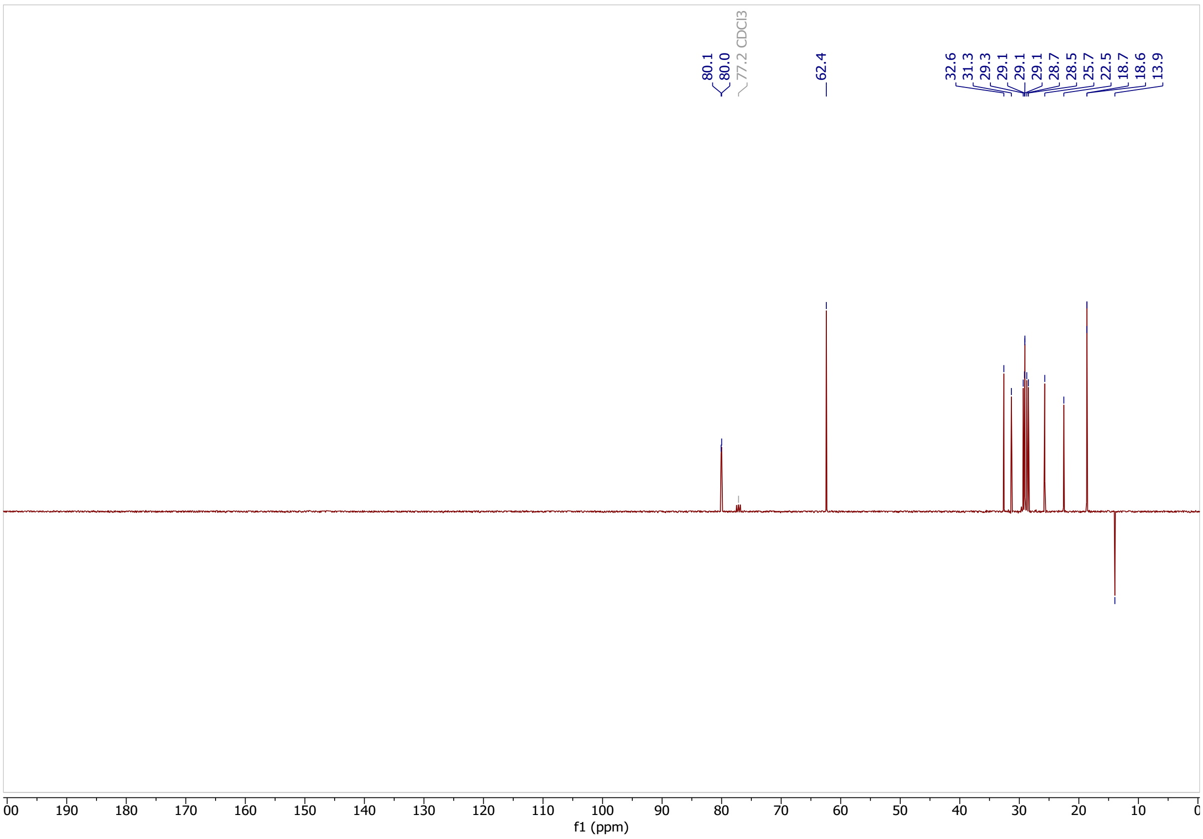

## hexadec-9-ynoic acid (S11)

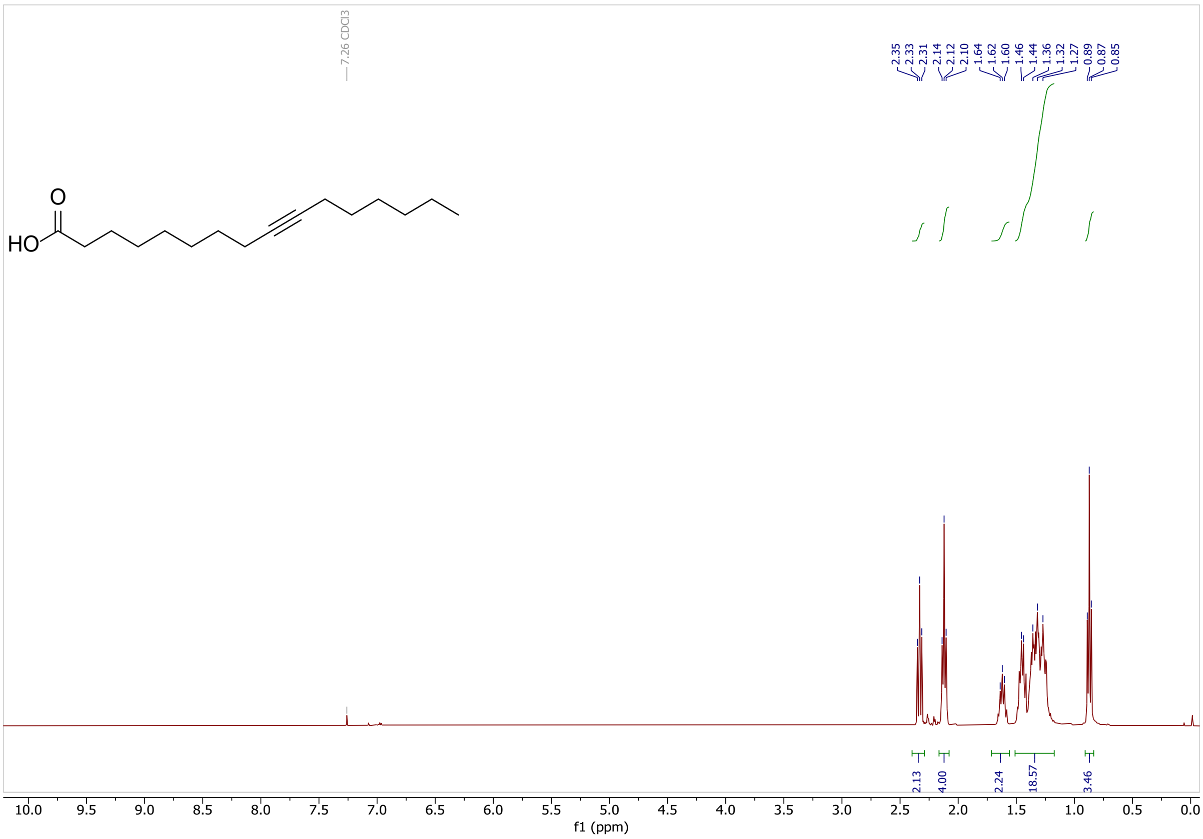


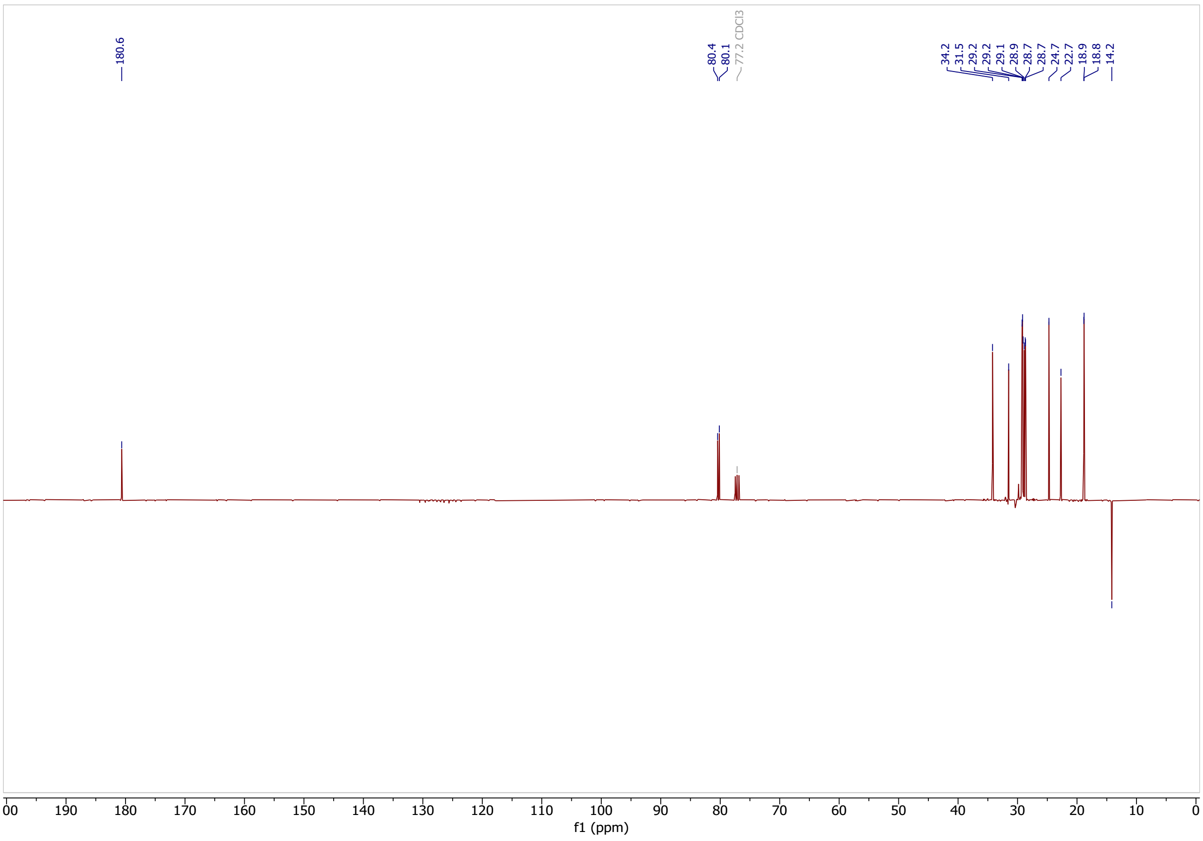

**methyl hexadec-9-ynoate (S12)**

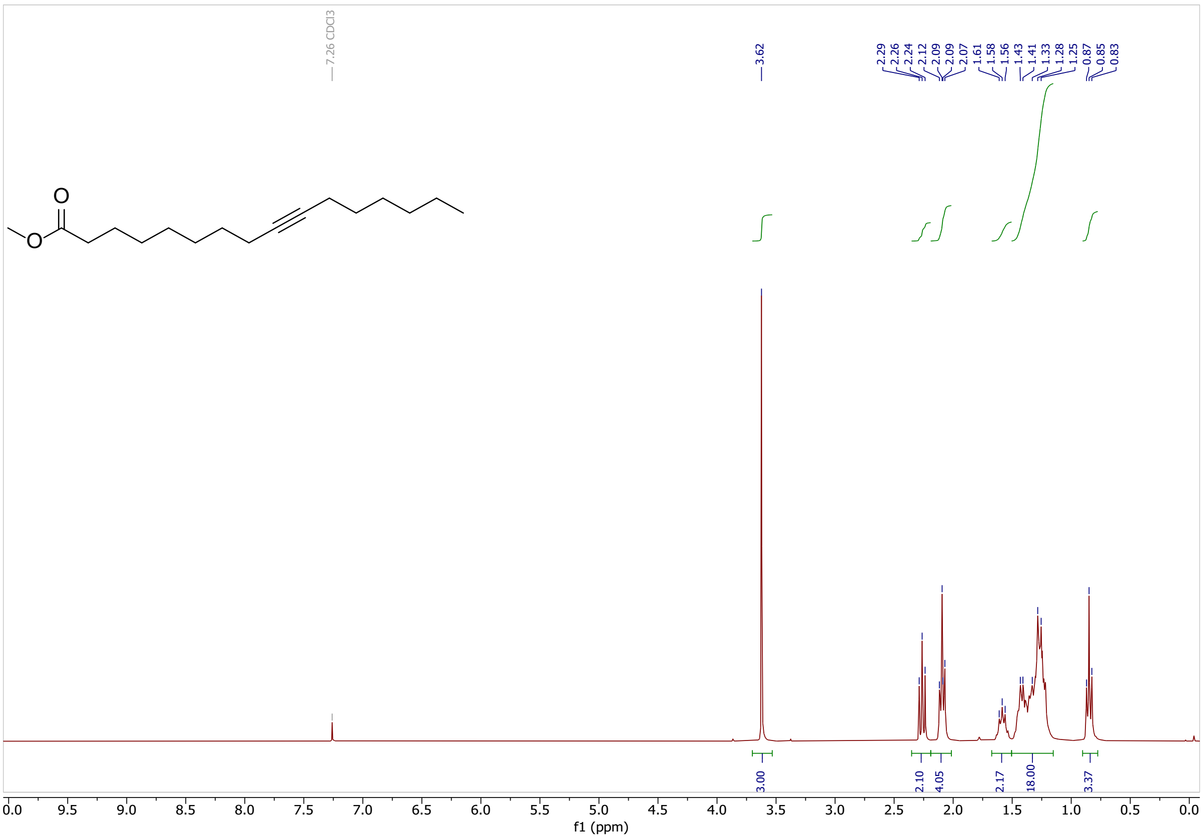


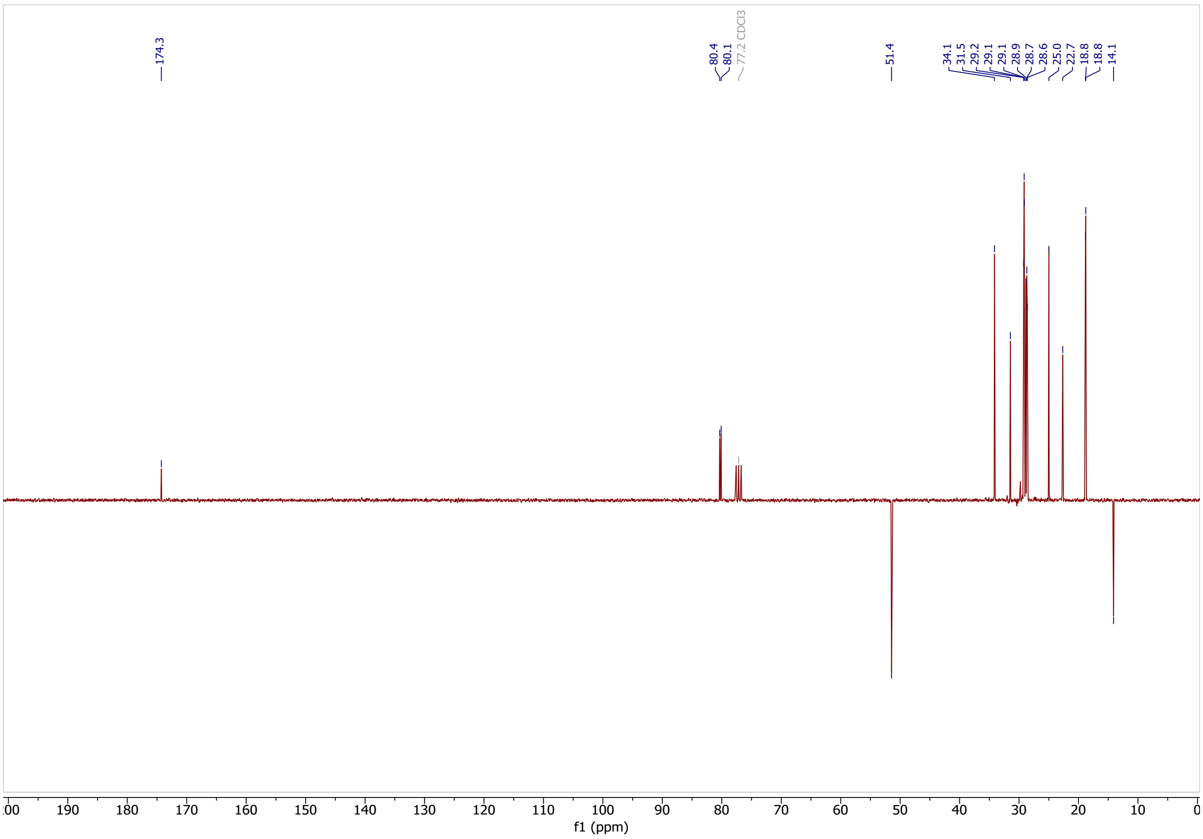

**ethyl 2-hexyl-3-(8-methoxy-8-oxooctyl)cycloprop-2-ene-1-carboxylate (S13)**

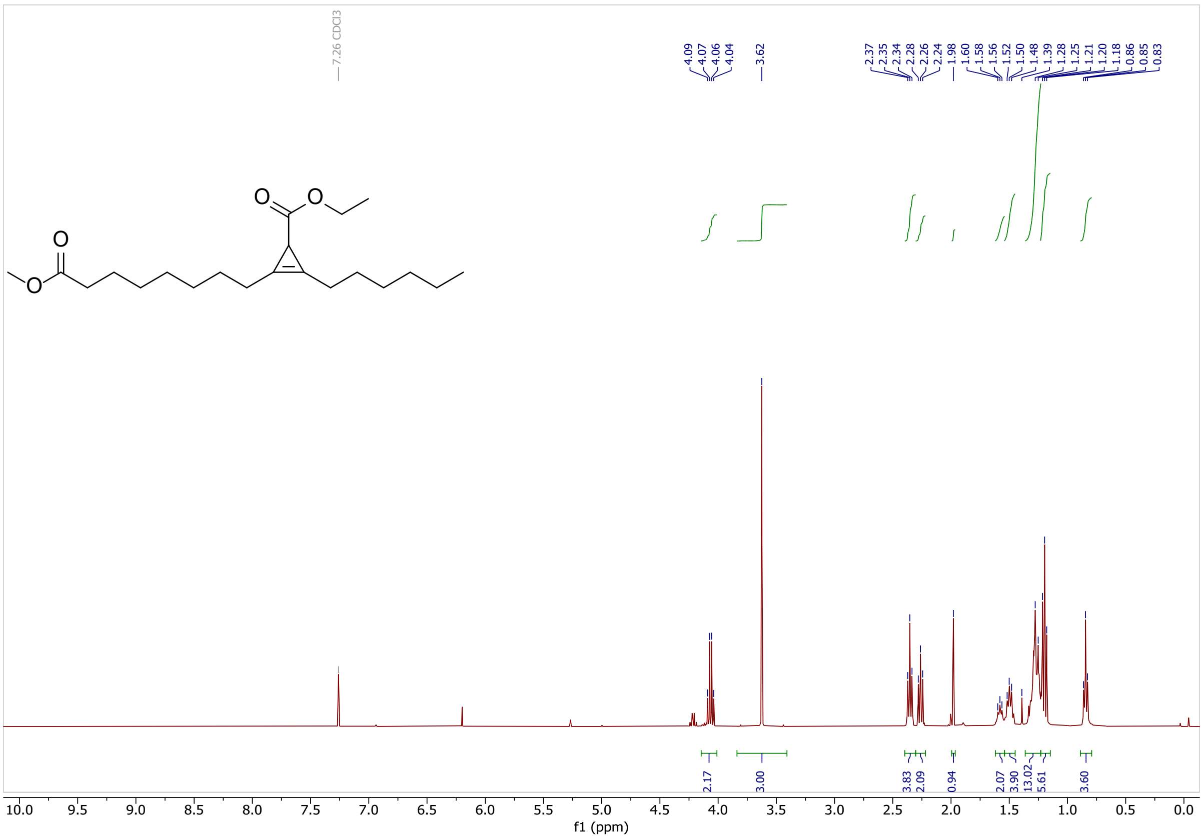


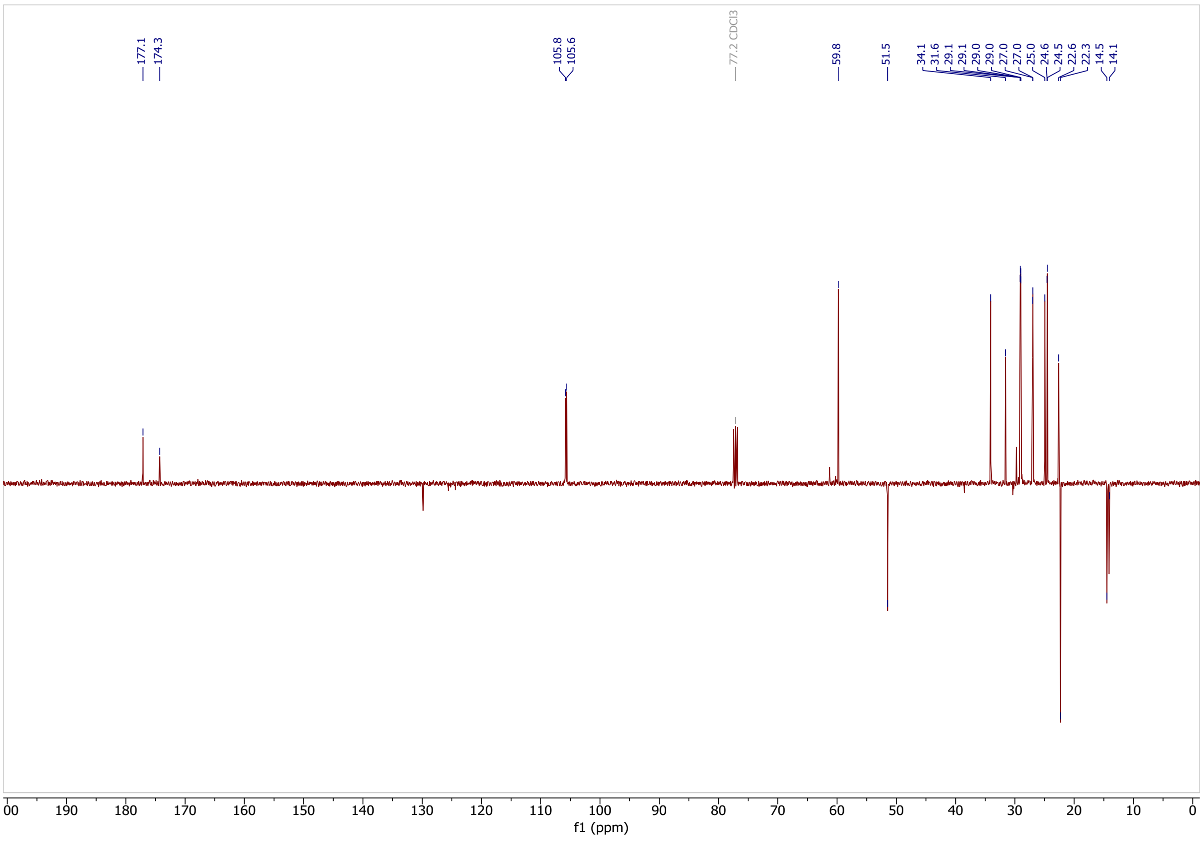

**2-(7-carboxyheptyl)-3-hexylcycloprop-2-ene-1-carboxylic acid (S14)**

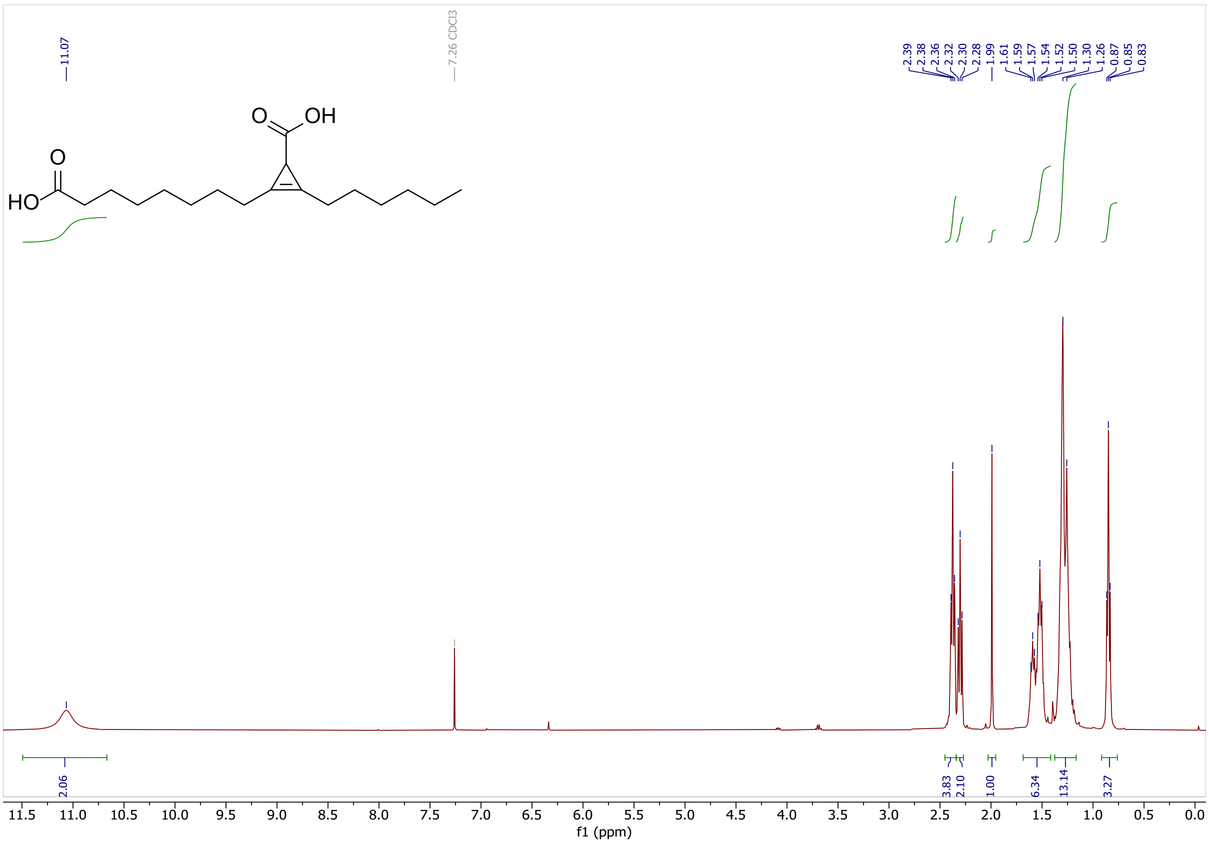

# 8-(2-hexylcycloprop-1-en-1-yl)octanoic acid (3)

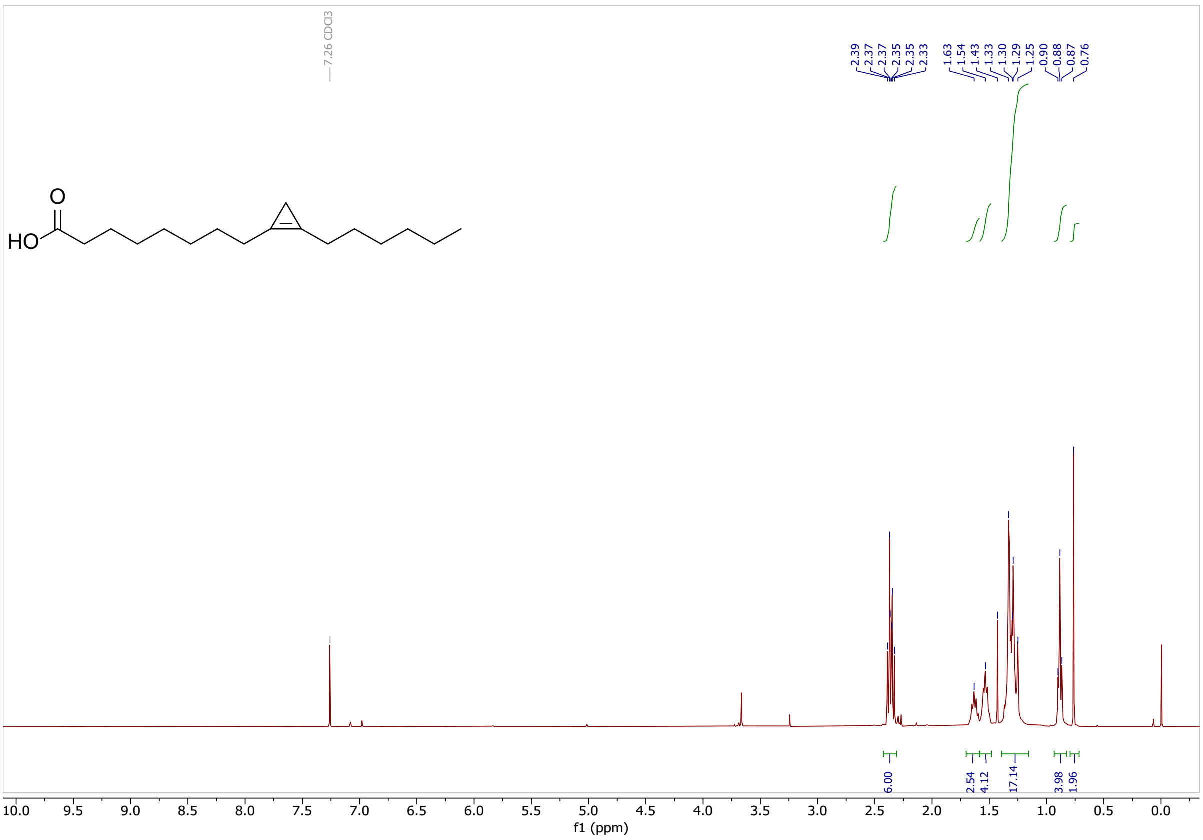


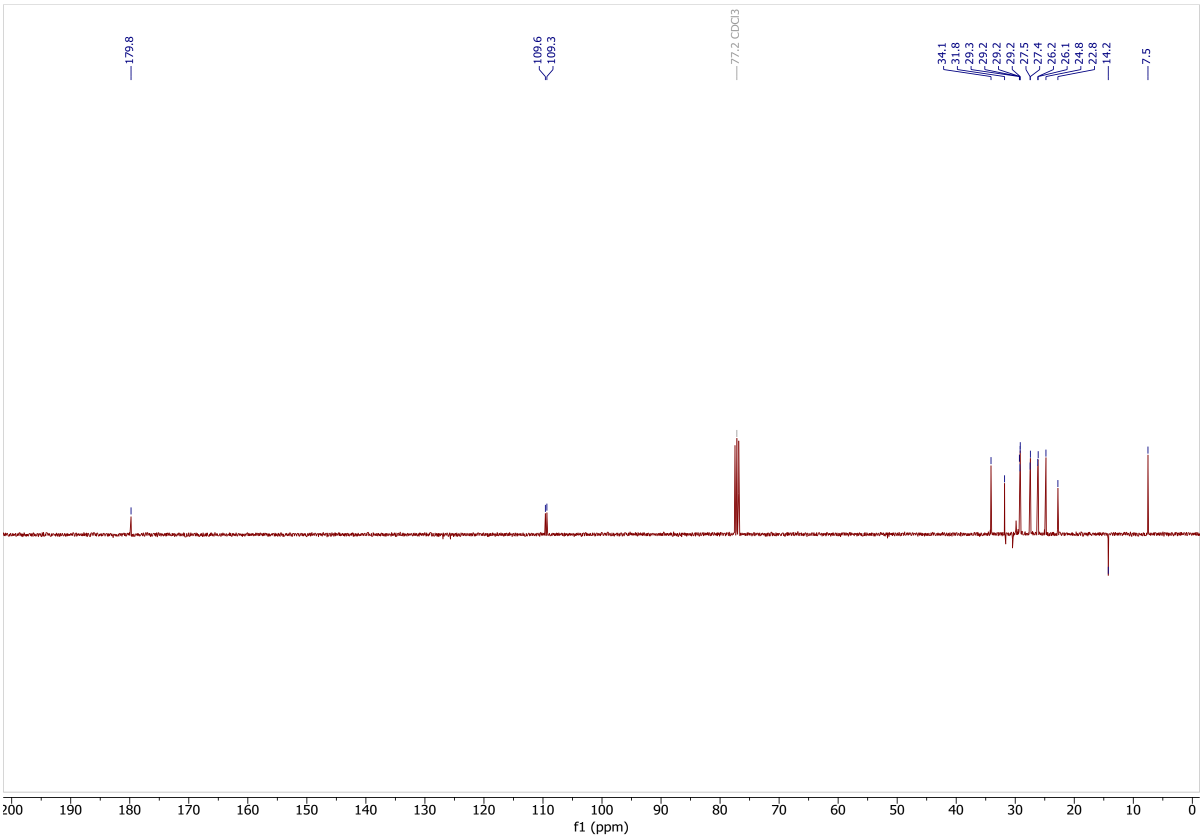

Supplement: Supplementary file 1 — Supporting Figure S1, Tables S1 and S2, and Schemes S1–S4 and experimental information are associated with this manuscript. The authors have cited additional references within the Supporting Information [97, 98, 99, 100, 101, 102, 103, 104, 105, 106, 107, 108, 109, 110, 111, 112, 113]. Supporting File 1: anie72785‐sup‐0001‐SuppMat.pdf. [file ANIE-65-e25040-s001.pdf]
